# Transcript Expression Data from Human Islets Links Regulatory Signals from Genome-Wide Association Studies for Type 2 Diabetes and Glycemic Traits to Their Downstream Effectors

**DOI:** 10.1371/journal.pgen.1005694

**Published:** 2015-12-01

**Authors:** Martijn van de Bunt, Jocelyn E. Manning Fox, Xiaoqing Dai, Amy Barrett, Caleb Grey, Lei Li, Amanda J. Bennett, Paul R. Johnson, Raymond V. Rajotte, Kyle J. Gaulton, Emmanouil T. Dermitzakis, Patrick E. MacDonald, Mark I. McCarthy, Anna L. Gloyn

**Affiliations:** 1 Oxford Centre for Diabetes, Endocrinology & Metabolism, University of Oxford, Oxford, United Kingdom; 2 Wellcome Trust Centre for Human Genetics, University of Oxford, Oxford, United Kingdom; 3 Alberta Diabetes Institute, University of Alberta, Edmonton, Alberta, Canada; 4 Department of Pharmacology, University of Alberta, Edmonton, Alberta, Canada; 5 Nuffield Department of Surgical Sciences, University of Oxford, Oxford, United Kingdom; 6 Department of Surgery, University of Alberta, Edmonton, Alberta, Canada; 7 Department of Genetics, Stanford University, Stanford, California, United States of America; 8 Department of Genetic Medicine and Development, University of Geneva, Geneva, Switzerland; 9 Institute for Genetics and Genomics in Geneva (iG3), University of Geneva, Geneva, Switzerland; 10 Swiss Institute of Bioinformatics, Geneva, Switzerland; 11 Oxford NIHR Biomedical Research Centre, Churchill Hospital, Oxford, United Kingdom; University of Chicago, UNITED STATES

## Abstract

The intersection of genome-wide association analyses with physiological and functional data indicates that variants regulating islet gene transcription influence type 2 diabetes (T2D) predisposition and glucose homeostasis. However, the specific genes through which these regulatory variants act remain poorly characterized. We generated expression quantitative trait locus (eQTL) data in 118 human islet samples using RNA-sequencing and high-density genotyping. We identified fourteen loci at which *cis*-exon-eQTL signals overlapped active islet chromatin signatures and were coincident with established T2D and/or glycemic trait associations. ‎At some, these data provide an experimental link between GWAS signals and biological candidates, such as *DGKB* and *ADCY5*. At others, the *cis*-signals implicate genes with no prior connection to islet biology, including *WARS* and *ZMIZ1*. At the *ZMIZ1* locus, we show that perturbation of *ZMIZ1* expression in human islets and beta-cells influences exocytosis and insulin secretion, highlighting a novel role for *ZMIZ1* in the maintenance of glucose homeostasis. Together, these findings provide a significant advance in the mechanistic insights of T2D and glycemic trait association loci.

## Author Summary

Genetic studies have uncovered many different parts of the genome playing a role in the risk of developing diabetes, or affecting blood sugar levels in the normal population. However, it has so far been difficult to tie these parts of the genome to genes that are responsible for the observed changes in risk and/or blood sugar levels (“effector transcripts”). It is clear from the genetic data that one of the key tissues in these phenotypes is the human pancreatic islet of Langerhans, but the limited availability of this tissue has been a major hurdle in translating the genetics into biology. Here, we present a study linking genetic variation to gene expression changes in 118 islet preparations. Using these *cis*-eQTLs, we provide candidate effector transcripts at 14 regions of the genome previously associated with glucose phenotypes. Many of the genes implicated through this approach have no known role in the islet. By experimentally changing the expression levels of one of these novel genes, *ZMIZ1*, in human islets and beta-cells, we uncovered a novel role for *ZMIZ1* in exocytosis and insulin secretion. These findings therefore significantly improve the discovery of biology underlying type 2 diabetes and glucose trait association.

## Introduction

Genome-wide association studies (GWAS) have identified approximately 80 loci robustly associated with predisposition to type 2 diabetes (T2D) [1–3] and a further 70 influencing a range of continuous glycemic traits [4–10] in non-diabetic subjects. There is substantial, though far from complete, overlap between these two sets of loci. Physiological studies in non-diabetic individuals indicate that most of these loci primarily influence insulin secretion rather than insulin sensitivity, highlighting a key role for the pancreatic islets of Langerhans in the mechanistic underpinnings of these association signals [11,12]. These findings have motivated efforts to catalogue the epigenomic and transcriptional landscape of human islets and to apply these findings to deliver biological insights into disease pathogenesis. Recently, it has been shown, for example, that GWAS signals for T2D and fasting glucose show significant co-localization with islet enhancers [13,14].

The identification of variant associations mapping to islet regulatory elements raises the question of which downstream (or “effector”) transcripts are responsible for mediating those regulatory effects. Relatively few of the T2D GWAS regions feature compelling biological candidates. The identification of *cis*-eQTL (expression quantitative trait locus) signals, especially in disease-relevant conditions and tissues, has, in other contexts, proven a powerful approach for connecting regulatory association signals to their effector transcripts [15–17]. Another major advantage of *cis*-eQTL data is that, by providing a direction of effect at the transcript level, they can help clarify whether genetic associations affect their phenotype through gain or loss of function–crucial information for translating the genetic findings into therapeutic options. Until now, difficulties in amassing adequate numbers of purified human islet samples have been a barrier to applying this approach at scale in this key tissue. Human islet material is not, for example, available through resources such as the Genotype-Tissue Expression (GTEx) project [18]. In this study, we set out to generate eQTL data from human islet samples, and to establish the extent to which this allowed us to identify candidate effector transcripts at GWAS loci for T2D and glycemic traits.

## Results

### Characteristics of cis-exon-eQTLs in human islets

We performed eQTL mapping in islet preparations from 118 human cadaveric donors of Northern European descent (isolated in Oxford, UK [n = 40], and Edmonton, Canada [n = 78]) to elucidate molecular mechanisms underlying both physiological and pathological variation in glucose homeostasis. Expression levels were profiled using RNA sequencing with 100 nucleotide paired-end reads on the Illumina HiSeq2000 platform. This generated an average of 72 million reads per sample uniquely mapping to exons (range 29–165 million). These were aligned to the GENCODE [19] v18 transcriptome reference. Genotypes were obtained using the Illumina HumanOmni2.5-Exome array (2,567,513 genotyped SNPs) with imputation from the 1000 Genomes Phase 1v3 cosmopolitan panel [20] providing data on up to 38,089,605 autosomal variants.

The islet consists of multiple cell types of which the insulin-secreting beta-cells are the most abundant. In line with this, the beta-cell secreted hormone insulin (*INS*) had, on average, 5-fold higher expression across all samples (an average RPKM [reads per kilobase of transcript per million reads mapped] of 58846) than the next most abundantly expressed protein-coding gene (S1 Fig). There was also high RNA expression of other canonical islet cell hormones including glucagon (*GCG*; average RPKM 4030), somatostatin (*SST*; average RPKM 1708) and pancreatic polypeptide (*PPY*; average RPKM 1452) (**S1 Fig**).

Islet eQTL analysis was performed using an additive linear model implemented in the R package MatrixEQTL [21]. For known common T2D and glycemic trait association loci, these data were integrated with genetic information (that is, patterns of association seen in large GWAS meta-analysis for T2D and continuous glycemic traits) and islet regulatory state maps [13,14]. We chose to focus on eQTL analyses at the level of the exon (as opposed to overall gene-level eQTLs), given that the former additionally captures variants that influence exon splicing. To account for variance attributable to factors such as donor characteristics, islet isolation center, purity, and storage (e.g. 55% of the samples had been cryopreserved for an extended period [22], see Methods), exon counts were normalized using gender and 15 PEER [23] factors derived from the normalized expression profile (these capture hidden covariates present in the data using Bayesian factor analysis methods). This normalization procedure successfully eliminated much of the structure observed in the raw data, most of which we attribute to experimental and technical factors

For each transcript, all variants within 1Mb flanking regions of the transcriptional start site (TSS) were tested for association. To correct for multiple testing (i.e. the many different *cis*-variants considered for each exon expression value), an empirical p-value was calculated from the most significant eQTL *p*-value per exon by permuting expression values between 1,000 and 10,000 times, while retaining the relation between expression value and covariates (see Methods). From this empirical *p*-value distribution, we calculated a false discovery rate (*q*-value) for each exon using the Storey method [24], imposing a study-wide false-discovery rate threshold of *q*<0.05. Across the 27,772 protein-coding and long non-coding (lncRNA) transcripts expressed in the human islet samples (expression was taken to be non-zero exon counts in at least 10% of individuals), we identified 2,341 genes that included at least one exon meeting this criterion (**S1 Table**).

The majority (90%) of significant islet exon-eQTLs was located within 250kb of the transcriptional start site, in line with observations in other tissues [17]. Even considering only the index variant for each of the significant islet exon-eQTLs, there is clear consistency with published islet chromatin maps: 735/2,341 (31%) variants overlapped enhancer or promoter signatures in at least one of the datasets [13,14] (**S1 Table**). When we discarded variants that had no chromatin annotation in either published map [13,14], the overlap with enhancers and promoters was even greater (59%; 735/1,252). The overlap of the 2,341 significant islet exon-eQTL variants with active islet chromatin signatures is significantly higher than that observed with 10,000 random samplings of 2,341 variants with no significant eQTL (2-fold enrichment, Fisher’s *p* = 1.7x10^-23^ with all variants; 1.7-fold enrichment, Fisher’s *p* = 5.7x10^-9^ when excluding non-overlapping variants).

We could also compare islet expression with RNA-Seq data for nine additional tissues analyzed, in approximately the same numbers of samples, as part of the GTEx project pilot study [18]. Since GTEx eQTLs are generated at the gene level, we reprocessed the data to generate exon-eQTLs. There was substantial sharing of islet exon-eQTLs across the full range of GTEx *p*-values with a mean estimated replication rate (*π*_*1*_[25]) of 70% (ranging from 66% [heart–left ventricle] to 73% [tibial artery]). There were, however, a total of 309 exons with an islet exon-eQTL that were expressed in at least one of the GTEx tissues (out of 1,659 such exons; 19%), but showed no association (*p*> = 0.05) in the GTEx data. These are likely to represent islet-specific regulatory regions.

### Identifying putative effector transcripts at GWAS loci likely to act through islets

Next, we focused on further analysis of the subset of *cis*-exon-eQTLs that mapped to the 82 known common variant T2D loci [1–3] and 49 loci for glycemic traits for which altered beta-cell function has been shown to be the main driver [4–10]. The latter included fasting glucose, fasting proinsulin, 2-hour glucose, HOMA-B, insulinogenic index, disposition index, corrected insulin response (insulin response to glucose after the first 30 minutes) and AUC_Insulin_/AUC_Glucose_ [4–10]. Seventeen of the glycemic trait loci overlap with T2D signals, whereas the other thirty-two are independent. To identify putative *cis-*effector transcripts for lead regulatory variants in these regions, we considered, for each of the regions, all genes with transcriptional start sites within 1Mb of any reported genome-wide significant lead variant (n = 218 variants). We adapted the genome-wide eQTL detection strategy describe above to identify, for each *cis*-region of interest, the single exon with the strongest *cis-*eQTL association. To minimize the possibility that co-localizing *cis*-eQTL and GWAS variants were tagging different functional variants (incidental overlaps are frequent given the abundance of *cis-*eQTL*s* in the genome), we required that the exon-eQTL index variant was in strong LD (1000 Genomes project CEU *r* ^*2*^>0.8) with the lead T2D or glycemic trait variant. We further verified the co-incidence of eQTL and GWAS variants by performing conditional analyses: specifically, we confirmed whether regressing out the variance explained by the T2D or glycemic trait lead GWAS variant eliminated, or at least, seriously depleted the *cis-*eQTL association signal. Within the GWAS regions, there were a total of 232 transcripts that met the study-wide significance criteria (i.e. *q*<0.05). Over 90% of the exon-eQTLs for these genes were statistically independent of the GWAS signal, but nine (marked by eleven GWAS index variants) met the LD criterion of *r* ^*2*^>0.8 and evidence for co-localization from the conditional analysis (**S2 Table**).

Since GWAS regions have a higher biological prior expectation of harboring an islet regulatory eQTL [13,14], we also considered an additional ten *cis-*eQTLs at which the statistical evidence did not reach study-wide significance, but which nonetheless displayed nominal significance (permuted *p*<0.05, corresponding to *q*<0.44; **S2 Table**) as well as meeting the other criteria related to GWAS signal overlap and conditional analysis. The combined set of twenty one variants was distributed over sixteen loci. With the exception of *AP3S2*, all showed a consistent direction of effect across the other exons of the implicated transcript (**S2 Table**). At two loci (*ABO* and *ZFAND6*), none of the variants in the set in strong LD (*r* ^*2*^>0.8) with the GWAS and exon-eQTL lead variants overlapped an islet-active regulatory state annotation in published datasets [13,14]. Whilst this does not necessarily exclude an effect on islet gene expression or relevance to the maintenance of glucose homeostasis, we did not consider these loci further.

We compared the islet eQTL data generated by the present study to that from a recent analysis of an entirely independent set of 89 human islets by colleagues in Sweden [26]. Though there were substantial experimental and processing differences between the two analyses, the present study replicated overlap of islet eQTL and GWAS signals at 80% (4/5) of the GWAS-related islet eQTLs reported in that study (*ABO*, *AP3S2*, *ERAP2*, and *MTNR1B*). Only two of these make it into our final list: at *ABO* there was no overlap with active islet chromatin, whilst at *ERAP2* conditional analysis could not confirm co-localization of eQTL and GWAS signal. There is also substantial replication of the genome-wide set of 616 eQTL signals described by Fadista *et al*. Of these 616, 503 had gene identifiers that could be mapped to the data described in this manuscript, with 43% (216/503) also having a significant (*q*<0.05) islet exon-eQTL (**S3 Table**). The observed gene-level replication rate is substantially higher than, for example, the 32% reported in a recent study [27] comparing two independent *cis*-eQTL mapping experiments in blood. The data reported by Fadista and colleagues uses gene-level rather than exon-level analyses. Nonetheless, we found that, amongst the 216 genes that had a cis-eQTL in both datasets, the same variant was associated in the majority of instances (56%—**S3 Table**).The vast majority (94%) of the 122 shared *cis*-eQTL signals are directionally consistent (**S3 Table**). This overlap provides reassurance that, despite technical and other challenges, and modest sample size, a high proportion of the *cis*-eQTL signals detected in these studies are robust.

The various filters described above left us with a set of nineteen variants, at fourteen loci, where multiple lines of evidence supported the candidacy of the exon-eQTL transcript as the effector for the relevant GWAS signal (**Table 1**; **S2 Table**). At four of these loci, the islet exon-eQTL overlapped GWAS variants that are genome-wide significant for both T2D and glycemic trait variation (*ADCY5*, *ARAP1*, *DGKB*, *MTNR1B*). At four others (*AP3S2*, *CDC123/CAMK1D*, *TMEM163*, *ZMIZ1*) the GWAS signal was for T2D alone. For the remaining six (*AMT*, *ANK1*, *FADS1*, *MADD*, *PCSK1*, *WARS*), the co-incident GWAS data implicated a range of continuous glycemic phenotypes (**Table 1**; **S2 Table**).

**Table 1:** Fourteen loci with co-localizing islet exon-eQTL and GWAS signals at loci for T2D and glycemic traits. Information on each of the fourteen loci for type 2 diabetes and/or glycemic traits where islet eQTL data provided putative effector transcripts. *Effect on gene expression is given for the allele associated with the trait effect directions in the column “Associated trait effects of eQTL allele”.

	Locus	Associated trait effects of eQTL allele	Implicated gene(s)	Full gene name(s)	Exon *q*-value	effect on *cis*-eQTL gene expression of risk allele	Directional consistency across exons
**Study-wide significant findings**	*ANK1*	Reduced corrected insulin response	*NKX6-3*	NK6 homeobox 3	4.03E-02	↓	3/3
	*AP3S2*	Increased T2D risk	*AP3S2*	Adaptor-related protein complex 3, sigma 2 subunit	5.73E-03	↓	3/7
	*ARAP1*	Increased T2D risk and fasting glucose, reduced fasting proinsulin	*STARD10*	StAR-related lipid transfer (START) domain containing 10	1.92E-02	↓	8/9
	*CDC123/CAMK1D*	Increased T2D risk	*CAMK1D*	Calcium/calmodulin-dependent protein kinase ID	1.06E-02	↑	11/11
	*DGKB/TMEM195*	Increased T2D risk and fasting glucose, reduced HOMA-B	*DGKB*	Diacylglycerol kinase, beta 90kDa	4.0E-02	↑	28/28
	*MADD*	Increased fasting glucose and fasting proinsulin	*MADD; ACP2*	MAP-kinase activating death domain; Acid phosphatase 2, lysosomal	5.73E-03	↑; ↑	39/39; 8/9
	*WARS*	Increased fasting glucose	*WARS*	Tryptophanyl-tRNA synthetase	5.73E-03	↓	17/19
**Additional findings with permuted *p*<0.05**	*ADCY5*	Increased T2D risk, fasting glucose and 2-hour glucose, reduced HOMA-B	*ADCY5*	Adenylate cyclase 5	1.83E-01	↓	26/26
	*AMT*	Increased fasting glucose	*RBM6*	RNA binding motif protein 6	1.47E-01	↓	23/24
	*FADS1*	Increased fasting glucose, reduced HOMA-B	*FADS1*	Fatty acid desaturase 1	2.62E-01	↑	13/14
	*MTNR1B*	Increased T2D risk and fasting glucose, reduced HOMA-B and corrected insulin response	*MTNR1B*	Melatonin receptor 1B	2.52E-01	↑	4/4
	*PCSK1*	Increased fasting glucose	*CTD-2260A17*.*2*	-	3.31E-01	↑	5/5
	*TMEM163*	Increased T2D risk	*MGAT5*	Mannosyl (alpha-1,6-)-glycoprotein beta-1,6-N-acetyl-glucosaminyltransferase	3.20E-01	↑	18/18
	*ZMIZ1*	Increased T2D risk	*ZMIZ1*	Zinc finger, MIZ-type containing 1	3.92E-01	↑	23/24

### Support for positional biological candidates

At three of the loci (*ADCY5*, *DGKB*, *FADS1*), the exon-eQTL data provide an independent empirical link between the GWAS signals and transcripts that already have strong biological candidacy with respect to glucose homeostasis. At *ADCY5*, where the GWAS variant influences T2D [3,4], fasting glucose [4], 2-hour glucose [10], HOMA-B [4] and birth weight [28], the rs11708067 A T2D-risk allele was associated with lower transcript expression levels (exon permuted *p* = 8.4x10^-3^, *q =* 0.183, *ß* = -0.44). This is consistent with a previous report, from a small candidate gene study [29], of a negative correlation between risk allele count and *ADCY5* expression levels. In human islets, ADCY5, a member of the adenylate cyclase family, is thought to couple glucose stimulation to insulin secretion, and this coupling is disrupted upon gene knockdown [29].

There are two independent T2D GWAS signals at the *DGKB* locus (lead variants rs2191349 and rs17168486) [3,4], separated by about 160 kilobases. At both, the T2D-risk allele is also associated with raised fasting glucose and reduced HOMA-B in non-diabetic individuals [3,4]. In the exon-eQTL data, both T2D-risk alleles independently drove higher expression levels of *DGKB* (rs2191349 signal, exon permuted *p* = 1.0x10^-3^, *q =* 0.040, *ß* = 0.41; rs17168486 signal, exon permuted *p* = 9.3x10^-3^, *q =* 0.194, *ß* = 0.52). Variant sets for both the 5’ of *DGKB* (rs17168486) and the more distal signal at rs2191349 overlapped islet chromatin signatures denoting either active promoters or enhancers [13,14]. DGKB is a subunit of diacylglycerol kinase, a regulator of the glucose-responsive secondary messenger diacylglycerol [30].

At *FADS1*, the GWAS allele associated with raised fasting glucose (in non-diabetic individuals) was implicated in increased islet expression of *FADS1* (exon permuted *p* = 1.6x10^-2^, *q =* 0.262, *ß* = 0.31). *FADS1* encodes the delta-5 fatty acid desaturase, which plays a role in the biosynthesis of highly unsaturated fatty acids. Variants in the same LD block as the fasting glucose GWAS variant are associated with altered blood levels of the substrate/product pair for the enzyme [31]. The lipid-related function of *FADS1* might appear, at first thought, to connect this locus to insulin sensitivity: however, the fasting glucose-raising allele [4] at this locus has also been associated with a lower HOMA-B [4] and insulinogenic index [12], consistent with an islet-mediated effect. The hypothesis that FADS1 might modulate insulin secretion through altered insulin sensitivity in the islet itself is supported by studies demonstrating the effects of fatty acid composition on insulin secretion both *in vitro* [32] and *in vivo* [33].

At two further *cis-*eQTL loci, our findings replicate previous studies. At the *MTNR1B* locus, the T2D-risk allele [1,3] also has a substantial impact on continuous glycemic traits (higher fasting glucose [4], lower HOMA-B [4] and corrected insulin response [8]). In the present study, as in two previous analyses of human islet expression [26,34], the same allele was associated with increased expression of the melatonin receptor 1B (exon permuted *p* = 1.5x10^-2^, *q =* 0.252, *ß* = 0.40). At the T2D-associated *CDC123/CAMK1D* locus [1,3], the islet *cis-*eQTL for *CAMK1D* (calcium/calmodulin-dependent protein kinase ID; exon permuted *p* = 2.0x10^-4^, *q =* 0.011, *ß* = 0.61) endorsed the designation of *CAMK1D* as the likely effector emanating from previous studies conducted in other tissues [18,35]. Recent work has demonstrated that the T2D-risk allele is associated with increased transcriptional activity in a luciferase reporter system [36], again consistent with the islet eQTL data.

### Multiple putative effector transcripts implicated

Whilst a single effector transcript was involved in the examples above, at certain other loci, the expression data are less conclusive. At the *ARAP1* locus, the islet exon-eQTL data link the T2D-risk allele [3] (also fasting glucose-raising [5,9], and fasting proinsulin-reducing [6]) to lower expression of *STARD10* (exon permuted *p* = 4.0x10^-4^, *q =* 0.019, *ß* = -0.39). This exon-eQTL is one of the 309 potentially islet-specific eQTLs based on comparison with data from nine GTEx tissues (see above). *STARD10*, which encodes StAR-related lipid transfer (START) domain containing 10, is thought to be involved in the regulation of bile acid metabolism [37], and has no reported role in the islets. At this locus, there have been reports, from human islet studies, of allele-specific expression of an alternative regional gene, *ARAP1*, encoding Arf-GAP with Rho-GAP domain, ANK repeat and PH domain-containing protein 1 [38]. The variants found to exhibit allele-specific expression were shown to affect promoter activity of the *ARAP1* P1 promoter in a dual luciferase system [38].

However, the published data on allelic imbalance in *ARAP1* are inconsistent [38,39], and we found no evidence of allelic imbalance for the relevant variant (rs11603334; Wilcoxon signed rank test *p*>0.1; **S2A Fig**) in our data. Neither was there any significant islet *cis*-eQTL signal for *ARAP1*. Therefore the data from this much larger islet cohort suggest *STARD10* rather than *ARAP1* as the likely effector transcript. Additional studies (e.g. conformational capture, CRISPR–Cas9 genome editing) will be instrumental in definitively assigning this locus to its effector transcript.

At the *AP3S2* locus, the T2D GWAS signal[3] coincided with an islet eQTL for *AP3S2*, encoding adaptor-related protein complex 3, sigma 2 subunit (exon permuted *p* = 1.0x10^-4^, *q =* 0.006, *ß* = -0.55). The identical signal was also detected in the recent report from an independent islet eQTL analysis [26]. However, in non-islet tissues, variants in strong LD with the T2D index variant have been reported as significant eQTLs for both *AP3S2* and *ANPEP*, a second regional gene which encodes alanyl (membrane) aminopeptidase [35,40]. Variants in *ANPEP*, although not in strong LD with the T2D signal, also showed allelic imbalance in human islets in both our data (**S2B and S2C Fig**) and a previous study by Locke *et al* [39]. Islet expression data for this locus, therefore, implicates both genes.

Variants at the *MADD* locus are associated with fasting glucose [4] and insulin processing defects [6]. At this locus, the islet exon-eQTL data implicated two regional transcripts: *MADD*, encoding MAP-kinase activating death domain (exon permuted *p* = 1.0x10^-4^, *q =* 0.006, *ß* = 0.25); and *ACP2*, encoding lysosomal acid phosphatase 2 (exon permuted *p* = 1.0x10^-4^, *q =* 0.006, *ß* = 0.31). Analysis of a beta-cell specific knockout mouse recently demonstrated that *Madd* plays a key role in glucose-stimulated insulin secretion, but the marked abnormalities of insulin processing that characterize the human GWAS signal were not observed [41], indicating that *MADD* might not mediate all the phenotypes associated with this signal. ACP2 is a lysosomal enzyme: disruption of the homolog in mice impacts lysosome function and causes cerebellar and skin abnormalities [42]. The known role of lysosomes in the degradation of aging insulin granules [43] provides a potential link between this gene and altered composition of the insulin secretory pool, which might explain the observed effects of the human association signal on fasting glucose and proinsulin levels.

These examples act as reminders of the importance of the independent validation of expression findings. They also highlight the potential for non-coding variants of interest to influence multiple transcripts, although this does not necessarily mean that all affected transcripts are involved in T2D pathogenesis.

### Identification of effector transcripts without a known role in islet biology

The mechanisms through which the other six implicated transcripts (*CTD-2260A17*.*2*, *MGAT5*, *NKX6-3*, *RBM6*, *WARS* and *ZMIZ1* at the *PCSK1*, *TMEM163*, *ANK1*, *AMT*, *WARS* and *ZMIZ1* loci, respectively) influence islet physiology are less clear.

The fasting glucose-raising allele at the *PCSK1* locus [5,9] was associated with increased expression of the uncharacterized protein *CTD-2260A17*.*2* (exon permuted *p* = 2.6x10^-2^, *q =* 0.331, *ß* = 0.58). However, at this locus there is strong biological candidacy of *PCSK1* [44], with coding variants in this gene thought to be causal for the association signal [9,45]. Loci where the underlying molecular mechanism affects protein function rather than regulation of transcript levels (also for example *SLC30A8*) are unlikely to be detected in eQTL studies. Therefore this raises doubts about the biological relevance of the association with *CTD-2260A17*.*2* expression at the *PCSK1* locus.

The gene implicated at the *TMEM163* locus was *MGAT5*, for which the T2D risk-increasing allele was associated with higher islet expression of the gene (exon permuted *p* = 2.4x10^-2^, *q =* 0.320, *ß* = 0.26). *MGAT5* encodes the protein *N*-glycosylation enzyme mannosyl (alpha-1,6-)-glycoprotein beta-1,6-N-acetyl-glucosaminyltransferase. The properties of cell surface receptors and transporters can be modulated through *N*-glycosylation; in beta-cells expression of the glucose transporter GLUT2 [46] and the incretin receptors [47] at the cell surface is, for example, altered by this process. Whole-body *Mgat5* knockout mice had improved insulin sensitivity and decreased gluconeogenesis [48], although effects on the beta-cell have not been studied. This direction of effect would be consistent with higher expression levels of *MGAT5* increasing risk of developing T2D.

At the *AMT* fasting glucose locus [5], the islet exon-eQTL implicated *RBM6* (exon permuted *p* = 5.9x10^-3^, *q =* 0.147, *ß* = -0.23). *RBM6* encodes RNA Binding Motif Protein 6, but neither the gene nor the protein has any defined phenotypic links. *NKX6-3*, which encodes NK6 homeobox 3, was implicated as the effector transcript for the *ANK1* locus variants influencing insulin secretion [8] (exon permuted *p* = 1.0x10^-3^, *q =* 0.040, *ß* = -0.36). The same region is also associated with T2D [3]. However, the T2D-risk variants are in comparatively low LD (*r* ^*2*^ = 0.14) with the corrected insulin secretion association signal, and no exon-eQTL signal was observed for these. NKX6.3 has a known role in the development of the gastrin-producing (G) and somatostatin producing (D) cells of the gastric endocrine system [49]. It is also active in the developing central nervous system [50]. There is no literature on the role of NKX6.3 in the islet, but, given the key role of other NKX6 transcription factors in the development of the endocrine pancreas [51], further follow-up of the islet consequences of altered *NKX6*.*3* expression is clearly warranted. The fasting glucose-raising allele [5] at the *WARS* locus was associated with markedly reduced *WARS* expression in human islets (exon permuted *p* = 1.0x10^-4^, *q =* 0.006, *ß* = -1.58). *WARS* encodes a tryptophanyl-tRNA synthetase involved in protein synthesis, regulated by cytokines and involved in cellular growth pathways such as angiogenesis [52]. It has, until now, not been allocated a role in the regulation of pancreatic islet function.

The final gene implicated by our data was *ZMIZ1*, encoding zinc finger, MIZ-type containing 1. *ZMIZ1* maps to a locus implicated in T2D-risk [3]. The *ZMIZ1* islet eQTL (exon permuted *p* = 3.8x10^-2^, *q =* 0.392, *ß* = 0.13) showed a consistent direction of effect across 23/24 *ZMIZ1* exons. The same *cis-*eQTL had a directionally consistent, although not significant, signal in the recently published independent islet expression [26]. It has not been detected in any other available *cis*-eQTL dataset, suggesting an islet-specific effect. To establish whether the putative effector transcripts identified by the exon-eQTL data provide novel biological inference, functional validation is essential. We used *ZMIZ1* as our exemplar for this purpose.

### The role of *ZMIZ1* in insulin secretion from human islets

**Fig 1:**
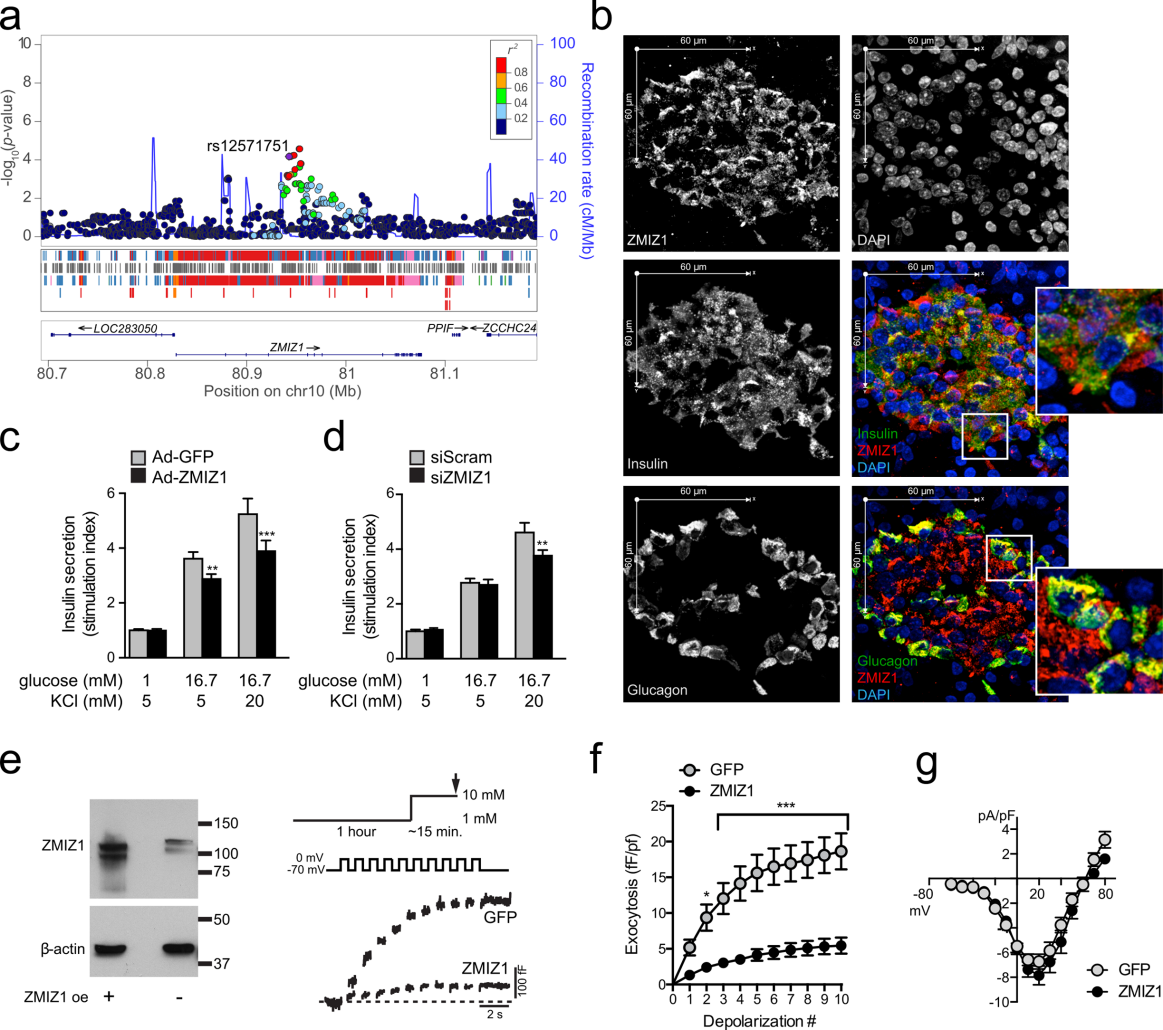
Islet eQTL data identifies ZMIZ1 as a novel gene involved in maintenance of glucose homeostasis in the human islet. (a) Regional plot showing the T2D-associated variant rs12571751 is in strong LD with the lead eQTL variant for ZMIZ1, and overlaps a long stretch of islet enhancer chromatin (denoted as red and blue in the tracks underneath the plot). (b) Immunofluorescence shows ZMIZ1 localizes to the islet within human pancreas sections, with staining in both alpha- and beta-cells. Effect of ZMIZ1 over-expression (c) and knockdown (d) on insulin secretion in human islets, showing significant (p<0.05) reduction in glucose- and KCl-stimulated insulin secretion during over-expression, and KCl-stimulated insulin secretion only during knockdown. (e) Western blot analysis confirms higher levels of ZMIZ1 after ZMIZ1 over-expression (left). Exocytosis was measured from single human beta-cells, expressing GFP alone or together with ZMIZ1, as increases in membrane capacitance during a train of membrane depolarizations. Representative traces (right) and (f) averaged data from 6 human donors (41–44 beta-cells) are show the significant (p<0.05) reduction in exocytosis in ZMIZ1-transfected beta-cells compared to GFP-controls. (g) Voltage-dependent Ca2+ currents were measured from human beta-cells expressing GFP alone or together with ZMIZ1. The average total Ca2+ charge entry during the depolarization (24–27 beta-cells from 3 individuals) was unchanged by ZMIZ1 over-expression.

At the *ZMIZ1* locus, the exon-eQTL index variant was in near complete linkage disequilibrium (*r* ^*2*^ = 0.98) with the T2D GWAS variant rs12571751, and overlapped an extended region of active islet enhancer chromatin (**Fig 1A**). Stretch enhancers such as this have been linked to cell-specific gene regulation [13] and, in human islets, to T2D [14]. Current understanding of ZMIZ1 function is limited, but it has been shown to act as a transcriptional co-regulator, playing a regulatory role in the p53 [53], Notch [54] and Smad [55] signaling cascades, and as a PIAS-like E3 SUMO-ligase [56]. Several variants in the wider region, independent of the T2D and islet eQTL signal (*r* ^*2*^<0.04), have been associated with a variety of autoimmune and inflammatory disorders (including inflammatory bowel disease and multiple sclerosis) [57,58], in addition to *ZMIZ1* expression in immune-relevant monocytes [15]. Our exon-eQTL approach has therefore highlighted a previously-unsuspected role for ZMIZ1 in pancreatic islet function, independent of the regional association to immune phenotypes.

Within human pancreas sections, ZMIZ1 was preferentially expressed in the islet and co-localized with both insulin and glucagon (n = 4 individuals; **Fig 1B**). Since *ZMIZ1* expression is higher in carriers of the T2D-associated rs12571751 A allele, we first determined the effects of *ZMIZ1* over-expression in dispersed human islet cells. We infected dispersed human beta-cells (n = 5 donors, 8 replicates for each condition in each donor) with a control adenovirus (Ad-GFP) or adenovirus expressing *ZMIZ1* (Ad-ZMIZ1). Increasing *ZMIZ1* (to 4520% of control expression levels, as confirmed by qPCR) impaired both glucose- and KCl-induced insulin secretion (20.5% and 25.8% reduction in stimulation index, *p*<0.01 and <0.001, respectively; **Fig 1C**). Knockdown of *ZMIZ1* in dispersed human islet cells (to 39.6% of control, confirmed by qPCR) had no significant effect on glucose-stimulated insulin secretion (also n = 5 donors, 8 replicates for each condition in each donor; **Fig 1D**), although KCl-induced insulin secretion was, paradoxically, reduced (*p*<0.05; **Fig 1D**).

To further explore the potential impact of *ZMIZ1* up-regulation, we measured exocytosis in human beta-cells directly. Upon membrane depolarization, fusion of insulin granule-containing secretory vesicles with the plasma membrane results in an increase in membrane surface area that can be detected by whole cell patch clamp as an increase in membrane capacitance. Over-expression of *ZMIZ1* reduced insulin exocytosis in individual human beta-cells to 29% of that in GFP-transfected controls (41–44 beta-cells from 6 individuals, *p*<0.001; **Fig 1E and 1F**). This represents a true impairment in exocytosis, rather than a reduction in the Ca^2+^ influx needed to trigger exocytosis, since voltage-dependent Ca^2+^ channel activity was unchanged by *ZMIZ1* over-expression (24–27 beta-cells from 3 individuals; **Fig 1G**). Together these data indicate a novel role for ZMIZ1 in the regulation of insulin secretion in human islets.

## Discussion

One of the key challenges faced in the biological interpretation of common variant GWAS signals lies in establishing the functional connections between causal variants within regulatory sequence and the downstream (or “effector”) genes through which they mediate their phenotypic effects. This is an essential step if we are to be effective in using human genetics to define pathways and networks central to the pathogenesis of common complex disease, and in identifying targets that may lead to novel preventative and therapeutic strategies. A range of complementary, bioinformatic and experimental, approaches are available to address this challenge. These include mapping the correlations between assays of chromatin state and cis-promoter activity [59], direct interrogation of local DNA interactions [60], and the search for coding variants in regional genes that recapitulate the disease phenotype [61].

In the present study, we demonstrate, through integration of human genetic disease association signals with information on patterns of exon-eQTLs and chromatin state in human islets, the potential for studies of human islet mRNA expression to implicate genes that play a previously unsuspected role in the maintenance of normal glucose homeostasis and the development of T2D. The focus on human islets was motivated by compelling evidence, from a variety of sources [1,11,13,14], which places islet dysfunction center-stage with respect to T2D pathogenesis. Despite this, and for understandable reasons to do with tissue accessibility and purity, human islets are largely absent from major eQTL and transcriptome cataloguing efforts such as GTEx [18], necessitating parallel efforts to define the interplay between DNA sequence variation and transcript expression in this key tissue.

As expected [17,62], the *cis*-exon-eQTL signals we detected in islets were a mixture of those shared across multiple tissues, and those that are islet specific. For example, 20% of the islet exon-eQTLs were not significant in any of the tissues studied in the GTEx pilot (though this may change as the GTEx sample size increases). Of the *cis*-eQTLs identified at GWAS loci for T2D and/or glycemic traits, only those involving *AP3S2* and *CAMK1D* had been identified as significant eQTLs in other tissues [18,35,40]. The *STARD10* islet exon-eQTL, for example, was not even nominally significant in any of nine GTEx tissues. These data emphasize the importance of extending such expression studies to the tissues most directly implicated in disease pathogenesis.

The identification of candidate effector transcripts through this and other routes motivates efforts to characterize the functional role of these genes in relevant cellular and animal systems. In the present study, we focused on one such gene, *ZMIZ1*, on the basis that the strength of the evidence for the *cis*-exon-eQTL was intermediate (it did not attain study-wide significance), and because it had no previous documented relationship to islet biology, other than localization within a T2D GWAS signal. We were able to show that *ZMIZ1* expression is localized to the endocrine pancreas (ruling out the possibility that the eQTL signal emanated from contaminating exocrine tissue), and that perturbation of *ZMIZ1* within the islet has a marked effect on exocytosis and insulin secretion, data that are clearly consistent with the designation of this gene as the likely mediator of the T2D association signal at this locus. Having said that, further work is required to fully enumerate the role of ZMIZ1 in the islet, to explain, for example, the apparently paradoxical reduction in KCl-stimulated insulin secretion observed in the knockdown experiment. This observation may be a consequence of the exaggerated attenuation of *ZMIZ1* expression in these experiments, when compared to the more subtle perturbation associated with the *cis*-eQTL.

As well as providing insights into transcript candidacy, these human eQTL studies are also informative with respect to the question of the directional impact of T2D-risk alleles on those genes. Recent studies of protein-truncating variants in *SLC30A8* [63] have demonstrated how crucial such information can be for guiding the design of potential pharmacological agents. Two examples are worth highlighting.

The islet exon-eQTL data presented here indicates that the T2D-risk allele at the *ADCY5* locus is associated with reduced expression of *ADCY5* and that reduced ADCY5 activity contributes to T2D pathogenesis. However, rare coding variants in *ADCY5* have been shown to be causal for a Mendelian disease phenotype characterized by neuromuscular features [64]. These rare Mendelian alleles act through gain of *ADCY5* function, and this is presumably why the phenotype of this condition (familial dyskinesia with facial myokymia) does not feature diabetes. This pattern of directional effects also diminishes the attraction of ADCY5 as a potential drug target for T2D.

In contrast, at *MTNR1B* the islet eQTL data presented here, along with several previous studies [26,34], tie the T2D-risk allele to increased expression of the cognate transcript. This replicated observation runs counter to a combined genetic and functional analysis of rare coding variants in *MTNR1B*, which reported that T2D risk was conveyed by alleles that reduced MTNR1B function [65]. Though increased MTNR1B transcript levels and reduced MTNR1B function could both be implicated in T2D susceptibility if reduced MTNR1B function was accompanied by changes in MTNR1B subcellular localization or a secondary increase of protein levels, the data by Bonnefond and colleagues [65] is not consistent with this explanation. It has also been proposed that these apparently contradictory findings could be explained by an absence of a negative feedback loop on *MTNR1B* expression in conditions of seriously impaired melatonin receptor function [65]. However, this appears inconsistent with the observation that islet expression of *MTNR1B* was entirely absent (below background, RPKM < 0.1) in 69% of individuals homozygous for the non-risk allele (and 37% of homozygous risk-allele carriers). These contrasting data hint at a complexity in the relationship between genetic variation and MTNR1B function that may only be resolved by direct assessment of the effects of melatonin on glucose homeostasis in human studies.

The present study represents the largest sample of human islet gene expression reported to date, but the sample size remains modest compared to those available for many other tissues. However, whereas association studies typically need effective sample sizes in the tens of thousands, the current islet eQTL study of 118 samples already identified putative effector transcripts at eight T2D loci. Physiological data had previously implicated a role for the islet at the majority of these loci, showing they affected beta-cell function [11]. This, combined with the extensive, but incomplete, overlap with the signals detected in a recent report of human islet expression [26], indicates that there is much to be gained by combining available data sets. Such efforts will likely generate many additional signals, at GWAS loci and beyond, as well as supporting additional analyses (e.g. of allele-specific expression). Similar studies in other T2D-relevant tissues will shed light on effector transcripts for loci that do not directly modulate insulin secretion–an example of this can be found at the *KLF14* locus, where eQTL studies in adipose tissue uncovered a large KLF14-regulated trans-eQTL network underlying the T2D association signal [16]. Data for non-islet tissues will also help answer whether loci that have been associated with changes in beta-cell function by *in vivo* studies in humans act directly on the islet or affect insulin secretion indirectly by altering, for example, expression in brain or gut.

As a more complete picture of the islet *cis*-eQTL landscape emerges, it will be highly informative to integrate these data with those obtained from the implementation of orthogonal, informatic and experimental, approaches for linking regulatory variants of interest to their transcriptional targets. Recent advances that enable scale up of conformational capture across multiple genomic regions are likely to be particularly relevant here [60]. Additionally, dense genomic annotations have become available for key T2D-relevant tissues, and similar data is being generated on islets at different developmental stages and after application of metabolic stimuli (e.g. comparing high versus low glucose culturing). This provides a rich framework for deriving functional inference from human genetics, and for identifying translational opportunities with respect to target identification and biomarker discovery.

## Methods

### Human tissue

Human islets were collected in two locations. Forty samples were freshly isolated at the Oxford Centre for Islet Transplantation (OXCIT) in Oxford, UK, as described [66], and processed for RNA and DNA extraction after 1–3 days in culture in CMRL media. In Edmonton, Canada, 65 samples were extracted from the long-term cryopreserved biobank and thawed as described [22], or were freshly isolated (n = 13) from donor pancreas as described previously [67]. For functional studies islets from a total of 12 donors were used (age = 52.4 +/- 3.9 years, 50% male, BMI 27.8+/-1.7). Pancreas biopsies were taken, fixed in Z-fix, and paraffin embedded prior to sectioning and immunostaining (described below). Isolated or thawed islets were cultured in CMRL media for 1–3 days prior to storage for RNA extraction or *in vitro* experimentation. Only freshly isolated islets were used for electrophysiology and insulin secretion studies. All studies were approved by the Human Research Ethics Board at the University of Alberta (Pro00001754), the University of Oxford's Oxford Tropical Research Ethics Committee (OxTREC Reference: 2–15), or the Oxfordshire Regional Ethics Committee B (REC reference: 09/H0605/2). All organ donors provided informed consent for use of pancreatic tissue in research.

### RNA extraction from human islets

RNA was extracted from human islets using Trizol (Ambion, UK or Sigma Aldrich, Canada). To clean remaining media from the islets, samples were washed three times with phosphate buffered saline (Sigma Aldrich, UK). After the final cleaning step 1 mL Trizol was added to the cells. The cells were lysed by pipetting immediately to ensure rapid inhibition of RNase activity and incubated at room temperature for ten minutes. Lysates were then transferred to clean 1.5 mL RNase-free centrifuge tubes (Applied Biosystems, UK). For islet preparations isolated in Edmonton, Trizol fractions were shipped to Oxford before further processing.

For the phase separation, 200μL chloroform (Fisher Scientific, UK) was added to each tube. Samples were vigorously shaken to begin organic and aqueous phase separation. This was followed by a 5 minute incubation room temperature and 30 minute-spin at 12,000 x g and 4°C to complete phase separation. The aqueous phase containing the RNA was transferred to a clean 1.5ml RNase-free tube by pipette, and 500μl isopropanol (Fisher Scientific, Loughborough, UK) was added to precipitate the RNA. The remaining organic and DNA phases were used for DNA extraction (see below). The RNA solution was incubated for 5 minutes at room temperature and stored overnight at -20°C. The following day, RNA was pelleted by centrifugation at 12,000 x g for 50 minutes (4°C) and supernatant was carefully removed. The pellet was washed twice in 1 ml 75% ethanol (Sigma Aldrich, UK) before centrifugation at 12,000 x g for 30minutes. After the final ethanol wash was removed, the RNA pellet was allowed to air-dry for 10 minutes. To re-suspend the RNA, a minimum of 20μl RNase-free water (more as necessary for complete re-suspension) was added to each sample. RNA quality (RIN score) was determined using an Agilent 2100 Bioanalyser (Agilent, UK), with a RIN score > 6 deemed acceptable for inclusion in the study. Samples were stored at -80°C prior to sequencing.

### DNA extraction for genotyping

For the majority of samples, DNA was extracted from either spleen or the exocrine fraction of the islet isolation using the Tissue DNA Purification Kit according to manufacturer’s instructions on an automated Maxwell 16 system (both Promega, USA). When no other tissue was available, DNA was extracted from human islets using the Trizol fraction remaining after extraction of RNA (see above). To precipitate the DNA, 300μl 100% ethanol was added to the thawed solution. This mixture was incubated at room temperature for a minimum of 30 minutes. DNA was then pelleted by centrifugation at 4,000 x g for 5 minutes at 4°C. After removing the supernatant, the pellet was twice washed with 0.1M trisodium citrate (Sigma Aldrich, UK) in 10% ethanol and left at room temperature for 30 minutes, followed by another wash step with 75% ethanol. After the final wash step, pellets were air-dried for 10 minutes to remove residual ethanol and re-suspended in a minimum of 100 μL 8mM NaOH (Sigma Aldrich). Extracted DNA was stored at -20°C before further use.

### Genotyping and imputation

In total, 118 samples were genotyped on the Illumina Omni2.5+Exome genotyping array. Samples were prepared according to the Illumina Infinium protocol and run on the Illumina iScan platform at the Oxford Genomics Centre (Wellcome Trust Centre for Human Genetics, University of Oxford, Oxford, UK). Genotypes were called with Illumina GenCall software using the standard Illumina cluster file and default genotype calling cut-offs. The direct genotypes were then used for imputation. Principal component analysis was performed to confirm European ancestry of all samples (**S3 Fig**). Variants with a call rate < 99% and minor allele frequency (MAF) < 0.01, as well as those deviating from Hardy–Weinberg equilibrium (*p*<0.0001), were filtered out before imputation–leaving 1,323,351 variants. Haplotypes were inferred from these genotype data using SHAPEIT [68]. Genotypes were imputed into the phased haplotypes using IMPUTE2 [69] with the entire 1000 Genomes Phase 1 v3 release [20] as the reference panel. For the QTL analysis, we used 5.8 million imputed autosomal single nucleotide variants with an INFO score > 0.4 and MAF > 0.05.

### RNA sequencing and expression quantification

Poly-A selected libraries were prepared from total RNA at the Oxford Genomics Centre using NEBNext ultra directional RNA library prep kit for Illumina with custom 8bp indexes [70]. Libraries were multiplexed (3 samples per lane), clustered using TruSeq PE Cluster Kit v3, and paired-end sequenced (100nt) using Illumina TruSeq v3 chemistry on the Illumina HiSeq2000 platform. Samples were mapped with TopHat2 [71] on default settings with GENCODE v18 [19] as transcriptome and GRCh37 as genome reference. Exon level reads counts for all protein-coding and long non-coding transcripts present in GENCODE v18 were quantified with RNA-SeQC [72] with the “*strictMode*” flag set. Transcript level counts were compiled by adding up the counts for all exons. The sequenced data was required to contain at least 10M mapped and properly paired reads after applying the quality filters.

### Expression normalization and eQTL analysis

First, exons with no expression in 10 or more samples were removed. To normalize for variation in read depth across samples, exon counts were scaled to the median number of exon-mapping reads per sample. The scaled exon counts were *log*_*2*_-normalized followed by per exon transformation to a standard normal (to minimize the effects of outliers in the linear regression). *Even considering only the index variant* variation in the QTL analysis, we derived 15 synthetic covariates from the normalized exon profile using PEER with default settings [23]. Since none of the 15 PEER factors were significantly correlated (*q*-value < 0.05) with gender, we added this as an additional covariate. The QTL analysis was performed on all SNP-exon pairs within 1Mb flanking regions of the transcripts transcriptional start site (TSS) using linear regression assuming an additive model as implemented in MatrixEQTL [21]. To correct for multiple testing per gene expression phenotype, we permuted the expression labels per samples (while maintaining the relation between PEER factors and expression labels) and compared the minimum *p*-value for each permutation against the minimum observed *p*-value until at least 15 more extreme *p*-values were observed (with a minimum of 1,000 and maximum 10,000 of permutations). From these data we calculated a permuted *p*-value for each exon. False-discovery rate across the permuted *p*-values for all exons estimated using the *q*-value method [24], with a *q*<0.05 threshold used for identifying study-wide significant islet exon-eQTL genes. For the overlap between GWAS loci and islet eQTLs we additionally considered all exons with a permuted *p*<0.05, with the best exon used per locus.

### Exon eQTL calls from GTEx pilot data

To determine the islet exon-eQTLs sharing across tissues, we generated exon-eQTL calls for the GTEx pilot dataset [18]. We used reference files and exon count from the GTEx portal (http://www.gtexportal.org/home/datasets2, last accessed on 30 August 2015), and genotype files available through dbGaP. Exon counts were processed as described above. We replaced the 15 GTEx-supplied gene-level PEER factors with those derived from the normalized exon counts, while retaining the other GTEx covariates. Finally, exon-eQTL mapping was performed as described above.

### Immunohistochemistry

Human pancreatic biopsies were fixed in Z-fix (Anatech, USA), paraffin embedded, and sliced into 5μm sections. Sections were rehydrated and antigen unmasking performed. Immunostaining was performed for insulin (Santa Cruz Biotechnology Inc., USA), glucagon (EMD Millipore, USA) as previously described. The antibody targeting ZIMZ1 (ZIMPZ10; sc-82438 Santa Cruz Biotechnology Inc. 1:50, overnight incubation) recognizes an N-terminal epitope. All slides were coverslipped with prolong gold antifade and visualized on a WaveFX spinning disk confocal (Quorum Technologies, Canada) using a 40X/1.3 NA lens and 405,491,561, and 642nm excitation lasers coupled with matched filter sets. Images were captured on a Hamamatsu EMC9100-13 camera (Hamamatsu Corp, USA) using Volocity imaging software (Perkin Elmer, Canada). Analysis of images was performed using Volocity and ImageJ (NIH).

### Electrophysiology studies

Human islets were hand-picked to purity and dispersed using enzyme-free cell dissociation buffer (Life Technologies, Canada). Cells were plated on 35mm dishes and transfected with control (pEGFP-N1, Clontech, Mountain View, CA, USA) or ZMIZ1 over-expression (ZMIZ1 pCMV6- AC-GFP, Origene, Rockville, MD, USA) plasmids via lipid transfection (Lipofectamine 2000, Life Technologies, Canada). Following 48hrs post-transfection culture we used the standard whole-cell techniques with the sine+DC lockin function of an EPC10 USB amplifier and Patchmaster software (HEKA Electronics, Germany) to measure capacitance during a series of ten depolarizations of 500ms each from -70 to 0mV. Experiments were performed at 32–35°C. Extracellular bath solution for depolarization trains contained (in mM): 118 NaCl, 20 TEA, 5.6 KCl, 1.2 MgCl_2_, 2.6 CaCl_2_, 10 glucose and 5 HEPES (pH7.4 with NaOH). Dishes were preincubated for one hour in culture media with 1mM glucose before capacitance measurements. Pipette solution for depolarization trains contained (in mM): 125 Cs-glutamate, 10 CsCl, 10 NaCl, 1 MgCl_2_, 0.05 EGTA, 5 HEPES, 0.1 cAMP and 3 MgATP (pH 7.15 with CsOH). To measure voltage-dependent Ca^2+^ channel activity, using Ba^2+^ as a charge carrier, the pipette solution contained (in mM): 140 Cs-glutamate, 1 MgCl_2_, 20 tetraethylammonium chloride, 5 EGTA, 20 HEPES and 3 MgATP (pH 7.3 with CsOH). The bath contained (in mM): 20 BaCl_2_, 100 NaCl, 5 CsCl, 1 MgCl_2_, 5 glucose, 10 HEPES, and 0.5 μM tetrodotoxin (pH 7.35 with CsOH). Patch pipettes, pulled from borosilicate glass and coated with Sylgard, had resistances of 3-4megaohm (MΩ) when filled with pipette solution. Whole-cell capacitance responses were normalized to initial cell size and expressed as femtofarad per picofarad (fF/pF) or picoampere per picofarad (pA/pF).

### Insulin secretion

Human islets were hand-picked to purity and dispersed using Accutase (Life Technologies, Canada) and plated in a 96 V-well plate at a density of 5000 cells/well. ZMIZ1 over-expression (AdZMIZ1 or AdGFP, Welgen Inc., USA) or siRNA knockdown (siZMIZ1 or siScrambled, Life Technologies) was performed at the time of plating. Cells were cultured in CMRL 1066 (Corning, USA) supplemented with 0.5% bovine serum albumin (Equitech-Bio Inc., USA), 1% insulin transferrin selenium (Corning), 100 U/mL penicillin/streptomycin (Life Technologies) and L-glutamine (Sigma-Aldrich) at 37°C, 5% CO_2_. Insulin secretion experiments were performed after 24 hours (over-expression) or 48 hours (siRNA knockdown) culture in incubation buffer containing (in mM): 115 NaCl, 5.0 KCl, 24 NaHCO_3_, 2.2 CaCl_2_, 1 MgCl_2_, 0.25% BSA, 24 HEPES (pH7.3 with NaOH). Cells were pre-incubated for 45 minutes at 1mM glucose, followed by 1hour stimulation with 1mM glucose, 16.7mM glucose or 16.7mM glucose plus 20mM KCl. Samples were collected at stored at -80°C prior to assay by electrochemiluminescence (Meso Scale Diagnostics, USA). To account for the normal variation in secretory responses between donors, data was normalized to the control 1 mM glucose condition and presented as stimulation index (SI; fold increase). Data were analyzed by repeated measures two-way ANOVA and Tukey post-test.

### Data access

Genotype and sequence data have been deposited at the European Genome-phenome Archive (EGA; http://www.ebi.ac.uk/ega/), which is hosted by the European Bioinformatics Institute (EBI), under accession number EGAS00001001265.

## Supporting Information

S1 Table All 2,341 genes with a significant islet exon-eQTL (best exon reported) with direction of effect and overlap of index SNP with published islet chromatin maps. (PDF)

S2 Table Detailed information on the 21 reported index variants for T2D and glycemic traits co-inciding with islet eQTLs. (PDF)

S3 Table Overlap between islet exon-eQTLs and the gene-level eQTLs from Fadista et al. (PDF)

S1 Fig Expression of the twenty five most abundantly expressed genes in human islets used in the study. Expression was quantified as reads per million mapped reads per kilobase of transcript (RPKM). Error bars denote standard error of the mean.
(PDF)

S2 Fig Replication of previous allele-specific expression findings. (a) Previously reported ASE variant in *ARAP1* (rs11603334) associated with T2D and glycemic traits showed no significant (*p*>0.1) allelic imbalance in the human islet data. (b,c) Both previously reported ASE variants in *ANPEP* (rs17240240 and rs41276922), which are in very weak LD with the T2D signal at the *AP3S2* locus, also show significant (p<0.01) ASE in this study.
(PDF)

S3 Fig Principal component analysis confirms European ancestry of islet samples. Principal component analysis of the 118 islet samples with the 1000 Genomes Northern European ancestry populations, computed using independent common (MAF > 1%) variants on chromosome 1.
(PDF)
