# Supplementary material for: Transcript Expression Data from Human Islets Links Regulatory Signals from Genome-Wide Association Studies for Type 2 Diabetes and Glycemic Traits to Their Downstream Effectors
Source: PLoS Genet. 2015 Dec 1;11(12):e1005694. doi: 10.1371/journal.pgen.1005694 (PMC4666611; doi:10.1371/journal.pgen.1005694)
Supplement: S1 Table — (PDF) [file pgen.1005694.s001.pdf]

Supplementary Table 1. All 2,341 genes with a significant islet exon-eQTL (best exon reported) with direction of effect and overlap of index SNP with published islet chromatin maps

Gene (exon)	SNP	Observed *p* -value	Permuted *p* -value	Permuted *q* -value	Direction of effect	Islet annotation - Pasquali	Islet annotation - Parker
RIBC2 (1)	rs71328696	2.40E-10	0.0001	0.0057	+	None	None
PRPF40A (3)	rs12693689	2.30E-08	0.0001	0.0057	+	None	None
LDHC (0)	rs6486421	9.30E-19	0.0001	0.0057	-	Repressed	Repressed
DGCR2 (11)	rs807758	3.60E-25	0.0001	0.0057	-	None	None
HLA-DQA1 (3)	rs17612669	1.10E-30	0.0001	0.0057	-	None	None
SPSB2 (0)	rs2071066	1.10E-09	0.0001	0.0057	+	Promoter	Promoter
HNRNPM (12)	rs59034702	5.10E-17	0.0001	0.0057	+	None	Transcription
RP11-326C3.2 (1)	rs760060	1.10E-08	0.0001	0.0057	+	Promoter	Promoter
ESYT2 (2)	rs6944346	5.20E-12	0.0001	0.0057	-	Promoter	Promoter
RP11-656D10.3 (0)	rs6600339	3.30E-16	0.0001	0.0057	-	None	None
IQGAP1 (10)	rs36158120	2.30E-08	0.0001	0.0057	-	None	None
LSMD1 (2)	rs7406902	1.80E-09	0.0001	0.0057	+	Enhancer	Enhancer
AP000345.1 (0)	rs2154589	6.00E-09	0.0001	0.0057	+	None	None
NPPA-AS1 (2)	rs198402	3.60E-09	0.0001	0.0057	-	Enhancer	Enhancer
CCDC163P (0)	rs12729974	9.20E-23	0.0001	0.0057	-	None	None
AMPD3 (7)	rs11605232	1.30E-07	0.0001	0.0057	+	None	None
TBC1D17 (12)	rs3745486	1.50E-12	0.0001	0.0057	-	Transcription	Transcription
CREB3L2 (11)	rs273975	3.00E-10	0.0001	0.0057	-	Enhancer	Enhancer
NOMO3 (13)	rs145526770	3.80E-09	0.0001	0.0057	-	None	None
MCC (19)	rs9326891	9.00E-13	0.0001	0.0057	+	Transcription	Transcription
AKAP9 (14)	rs6980247	3.70E-10	0.0001	0.0057	-	Transcription	None
RAI14 (2)	rs463001	3.60E-13	0.0001	0.0057	-	Promoter	Promoter
RP11-678G14.3 (0)	rs11881813	1.30E-10	0.0001	0.0057	+	None	None
NUTM2D (0)	rs79231017	3.10E-08	0.0001	0.0057	+	None	None
CAST (22)	rs34782647	7.10E-31	0.0001	0.0057	-	None	Transcription
CLK4 (12)	rs262023	3.40E-09	0.0001	0.0057	-	Transcription	Transcription
RP11-439A17.7 (0)	rs149241713	1.80E-21	0.0001	0.0057	-	None	None
ARHGEF3 (1)	rs1844474	7.80E-18	0.0001	0.0057	+	None	None
ARPC3 (1)	rs1426374	2.20E-07	0.0001	0.0057	-	None	None
USP36 (5)	rs55779573	3.80E-24	0.0001	0.0057	-	Transcription	Transcription
FAM21B (16)	rs55972166	1.80E-08	0.0001	0.0057	-	None	None
ENPP2 (6)	rs745383	3.80E-07	0.0001	0.0057	-	Transcription	None
UBE2D3 (11)	rs223435	3.00E-13	0.0001	0.0057	-	Transcription	Transcription
CTD-2192J16.21 (0)	rs4804718	2.10E-08	0.0001	0.0057	-	Transcription	None
SNHG9 (0)	rs30254	8.60E-13	0.0001	0.0057	+	None	None
ELP3 (21)	rs59148819	1.70E-17	0.0001	0.0057	+	None	Transcription
RP11-500G22.2 (1)	rs11200264	6.70E-10	0.0001	0.0057	-	Enhancer	Enhancer
FLVCR1-AS1 (1)	rs10779589	8.60E-20	0.0001	0.0057	+	None	None
RP11-344E13.3 (14)	rs77997617	3.40E-32	0.0001	0.0057	-	Transcription	Transcription
AC034243.1 (0)	rs6596449	9.40E-20	0.0001	0.0057	-	Transcription	None
DRAM2 (0)	rs12142849	5.80E-08	0.0001	0.0057	+	None	None
FAM214A (12)	rs4774636	4.30E-13	0.0001	0.0057	+	Enhancer	Promoter
PDLIM4 (3)	rs10463891	7.20E-08	0.0001	0.0057	-	Enhancer	Enhancer
LGALS9 (4)	rs113815962	7.30E-08	0.0001	0.0057	-	None	None
GSDMB (5)	rs907092	3.10E-08	0.0001	0.0057	-	Repressed	Repressed
LINC00359 (0)	rs1331274	2.40E-08	0.0001	0.0057	-	None	None
UGP2 (4)	rs6546032	1.40E-08	0.0001	0.0057	+	None	Enhancer
LDOC1L (0)	rs135623	5.30E-10	0.0001	0.0057	-	Enhancer	None
SLC2A1-AS1 (0)	rs114530232	6.10E-11	0.0001	0.0057	+	Promoter	Promoter
RP13-977J11.2 (0)	rs11246977	2.90E-10	0.0001	0.0057	+	Enhancer	Promoter
PIGC (7)	rs1052256	4.90E-12	0.0001	0.0057	+	Enhancer	Enhancer
RP11-977G19.11 (3)	rs60542959	2.60E-07	0.0001	0.0057	-	Promoter	Promoter
TRIM4 (0)	rs2060452	2.40E-10	0.0001	0.0057	+	None	None
PSCA (1)	rs11786721	4.90E-09	0.0001	0.0057	+	None	None
FIS1 (0)	rs6947016	1.30E-18	0.0001	0.0057	-	Promoter	Promoter
NAALAD2 (11)	rs12293182	2.80E-09	0.0001	0.0057	+	None	None
AP3D1 (28)	rs2240658	1.70E-13	0.0001	0.0057	-	Transcription	Transcription
AC012309.5 (0)	rs502546	8.30E-14	0.0001	0.0057	-	Transcription	Transcription
CABIN1 (17)	rs365158	1.40E-07	0.0001	0.0057	+	None	None
KLC1 (4)	rs11849259	5.40E-08	0.0001	0.0057	-	None	None
MEIS3 (5)	rs12459410	9.60E-10	0.0001	0.0057	-	None	None
PCDHA9 (0)	rs62624460	2.30E-10	0.0001	0.0057	-	Transcription	Promoter
HLA-DPB1 (2)	rs114988017	3.30E-20	0.0001	0.0057	-	None	Repressed
CCDC93 (0)	rs7588590	3.90E-08	0.0001	0.0057	+	None	None
NDUFS2 (14)	rs10797094	2.60E-09	0.0001	0.0057	-	Transcription	Transcription
LGALS7 (1)	rs2368519	5.90E-10	0.0001	0.0057	-	None	None
ASCC2 (19)	rs131301	2.50E-07	0.0001	0.0057	-	Transcription	Transcription
ATP6AP1L (9)	rs73138787	3.30E-10	0.0001	0.0057	+	None	Transcription
ATG10 (0)	rs3738	1.20E-08	0.0001	0.0057	+	None	Transcription
EP400NL (15)	rs12426136	1.50E-14	0.0001	0.0057	-	Enhancer	None
CAV1 (2)	rs4385407	1.00E-09	0.0001	0.0057	-	Repressed	Repressed
RP1-90J20.8 (0)	rs9378351	4.30E-11	0.0001	0.0057	+	Promoter	Enhancer
POR (15)	rs1057868	1.10E-09	0.0001	0.0057	+	Transcription	Transcription
COL24A1 (1)	rs11806244	1.30E-08	0.0001	0.0057	+	None	None
USP24 (8)	rs74542001	4.50E-08	0.0001	0.0057	-	None	None
CFHR1 (4)	rs12240143	4.00E-09	0.0001	0.0057	-	None	None
TMEM87B (4)	rs11123128	7.50E-23	0.0001	0.0057	-	Transcription	Transcription
GRHPR (2)	rs4878690	1.80E-08	0.0001	0.0057	+	Promoter	Promoter
WDR60 (23)	rs28679564	4.00E-08	0.0001	0.0057	-	Transcription	None
PROSER2 (3)	rs11257358	8.10E-10	0.0001	0.0057	-	None	None
CEP57 (3)	rs11021318	2.90E-18	0.0001	0.0057	-	Promoter	Promoter
CD22 (6)	rs10411704	9.40E-08	0.0001	0.0057	-	None	None
LRRC49 (20)	rs77066401	3.10E-17	0.0001	0.0057	+	None	None
ZNF791 (1)	rs58294682	3.70E-13	0.0001	0.0057	-	None	None
GIGYF2 (41)	rs7573208	1.50E-09	0.0001	0.0057	-	Transcription	Transcription
FAM221A (5)	rs34579167	4.00E-10	0.0001	0.0057	+	Transcription	None
SLU7 (15)	rs2910194	1.00E-09	0.0001	0.0057	-	Enhancer	Enhancer
IL10RB-AS1 (0)	rs13050728	2.30E-08	0.0001	0.0057	+	None	None
RFX7 (2)	rs8033653	1.40E-08	0.0001	0.0057	-	None	None
GLIPR1L2 (0)	rs4565940	1.20E-15	0.0001	0.0057	-	None	None
CYSTM1 (0)	rs35290793	1.90E-10	0.0001	0.0057	-	None	Promoter

ST3GAL1 (9)	rs4736696	2.90E-08	0.0001	0.0057	+	Enhancer	Enhancer
AC016747.3 (2)	rs2698197	1.10E-08	0.0001	0.0057	-	None	None
RP5-1021I20.1 (0)	rs12886003	2.20E-09	0.0001	0.0057	+	Enhancer	Enhancer
TMEM159 (6)	rs2005532	7.00E-22	0.0001	0.0057	-	Transcription	Transcription
RP11-611E13.2 (4)	rs11609337	1.00E-08	0.0001	0.0057	+	None	None
FBLN2 (3)	rs11128643	2.50E-09	0.0001	0.0057	-	Repressed	Repressed
AC073834.3 (1)	rs4893967	2.30E-09	0.0001	0.0057	-	None	None
GS1-251I9.4 (0)	rs12542580	8.90E-11	0.0001	0.0057	+	None	None
THRAP3 (1)	rs9659765	3.00E-09	0.0001	0.0057	-	Enhancer	Promoter
CBR3 (0)	rs13046907	9.70E-08	0.0001	0.0057	-	Enhancer	Enhancer
C21orf91-OT1 (6)	rs7277763	1.50E-12	0.0001	0.0057	+	Enhancer	None
C11orf91 (0)	rs4755893	3.50E-08	0.0001	0.0057	-	None	Enhancer
CTTNBP2NL (2)	rs2990988	3.30E-08	0.0001	0.0057	-	None	None
AC058791.2 (4)	rs1877727	7.70E-15	0.0001	0.0057	-	Transcription	Transcription
YWHAH (2)	rs4820059	5.90E-13	0.0001	0.0057	+	Enhancer	Enhancer
DUS3L (2)	rs2290777	1.20E-09	0.0001	0.0057	-	Promoter	Promoter
ATG14 (0)	rs68083077	8.30E-11	0.0001	0.0057	-	Enhancer	Promoter
CCDC180 (44)	rs6478014	8.00E-08	0.0001	0.0057	+	None	None
TMOD3 (1)	rs2959294	1.20E-09	0.0001	0.0057	+	Transcription	Transcription
RAD52 (0)	rs4388955	8.90E-10	0.0001	0.0057	-	Enhancer	None
HLA-B (0)	rs1055849	1.50E-15	0.0001	0.0057	-	Transcription	Promoter
ANKMY1 (11)	rs3821348	3.80E-11	0.0001	0.0057	-	Transcription	None
TSPAN12 (0)	rs200234817	1.90E-10	0.0001	0.0057	+	None	None
CNOT2 (0)	rs11609337	8.80E-08	0.0001	0.0057	+	None	None
CTD-2619J13.17 (0)	rs4801587	1.80E-08	0.0001	0.0057	-	None	None
PM20D1 (0)	rs823082	8.70E-08	0.0001	0.0057	-	None	None
HLA-C (0)	rs116108880	3.40E-31	0.0001	0.0057	-	Promoter	Promoter
PEPD (0)	rs17834067	1.70E-08	0.0001	0.0057	+	Enhancer	Enhancer
ZDHHC6 (7)	rs4918752	3.30E-10	0.0001	0.0057	-	Enhancer	Promoter
RP11-419C5.2 (0)	rs28659838	1.50E-25	0.0001	0.0057	-	None	None
ALDH7A1 (5)	rs73343315	1.30E-07	0.0001	0.0057	+	Transcription	Transcription
HAUS4 (5)	rs12880925	3.20E-09	0.0001	0.0057	-	Promoter	Promoter
FOXRED1 (0)	rs659551	2.80E-08	0.0001	0.0057	+	Transcription	Transcription
HDDC2 (1)	rs3799744	1.10E-07	0.0001	0.0057	-	None	None
INTS1 (18)	rs10224038	1.10E-07	0.0001	0.0057	-	Transcription	None
NMRK1 (1)	rs11144225	4.30E-10	0.0001	0.0057	-	Enhancer	Enhancer
MZT2A (2)	rs10179938	7.40E-13	0.0001	0.0057	+	Promoter	Promoter
AC000032.2 (0)	MERGED_DEL_2_4670_-	9.50E-19	0.0001	0.0057	-	None	None
SLC35A3 (2)	rs6701353	9.00E-09	0.0001	0.0057	-	None	None
C17orf97 (1)	rs7502594	6.10E-14	0.0001	0.0057	+	Promoter	Promoter
PARVA (5)	rs7935861	2.20E-15	0.0001	0.0057	+	Transcription	Transcription
RP11-96H19.1 (2)	rs4768697	4.50E-10	0.0001	0.0057	+	None	None
AC004562.1 (0)	rs8077036	2.10E-09	0.0001	0.0057	+	Transcription	None
ZBTB1 (1)	rs10131155	2.40E-09	0.0001	0.0057	+	Enhancer	None
NSF (11)	rs117120492	4.80E-11	0.0001	0.0057	+	None	Enhancer
LINC00202-1 (2)	rs11015399	1.30E-11	0.0001	0.0057	-	None	None
TRIM5 (14)	rs7940165	2.20E-10	0.0001	0.0057	+	None	None
ADAL (7)	rs28446815	1.80E-07	0.0001	0.0057	-	Repressed	None
ITGA7 (9)	rs113584176	2.20E-07	0.0001	0.0057	-	None	None
UNC79 (39)	rs72691017	3.70E-08	0.0001	0.0057	-	None	None
PLCB2 (1)	rs2229691	1.20E-08	0.0001	0.0057	+	None	Repressed
RP11-399E6.1 (1)	rs36084778	7.70E-09	0.0001	0.0057	+	None	None
DZIP3 (3)	rs7648331	1.80E-13	0.0001	0.0057	+	None	None
C2orf27A (0)	rs139905983	2.40E-08	0.0001	0.0057	-	None	None
ZNF83 (0)	rs11878446	4.50E-20	0.0001	0.0057	-	Transcription	None
SS18 (15)	rs2959360	8.90E-08	0.0001	0.0057	+	None	None
OSBPL2 (6)	rs6089702	8.00E-08	0.0001	0.0057	-	None	None
TBC1D5 (31)	rs13095510	7.10E-08	0.0001	0.0057	-	None	None
GTF2H2C (7)	rs34221525	1.20E-08	0.0001	0.0057	+	Enhancer	Promoter
SAYSD1 (1)	rs10305516	9.80E-12	0.0001	0.0057	-	Enhancer	None
AC104135.2 (0)	rs12366	3.60E-23	0.0001	0.0057	+	Promoter	Promoter
SLC35E2B (0)	rs79113395	1.80E-19	0.0001	0.0057	-	Promoter	Promoter
YBEY (1)	rs11909164	4.20E-16	0.0001	0.0057	-	Transcription	Enhancer
LRRC27 (12)	rs7090409	1.90E-11	0.0001	0.0057	+	Enhancer	None
MEGF8 (29)	rs75996891	3.00E-11	0.0001	0.0057	-	Promoter	Enhancer
AC093323.3 (0)	rs4689507	3.40E-10	0.0001	0.0057	-	Enhancer	Enhancer
RP11-217B7.2 (0)	rs2422493	6.10E-11	0.0001	0.0057	-	Enhancer	Enhancer
CAPG (2)	rs11689248	9.40E-09	0.0001	0.0057	+	None	None
CCDC57 (20)	rs62078307	2.50E-17	0.0001	0.0057	+	None	None
MAPT-AS1 (2)	rs113586173	9.20E-21	0.0001	0.0057	+	Enhancer	Enhancer
CHURC1 (1)	rs2296327	3.40E-23	0.0001	0.0057	-	Enhancer	Enhancer
TMEM8A (10)	rs3743888	1.20E-13	0.0001	0.0057	-	Enhancer	None
AC108004.3 (2)	rs7501541	5.60E-15	0.0001	0.0057	+	None	None
BTN3A1 (1)	rs3799378	7.80E-10	0.0001	0.0057	+	None	Enhancer
MKKS (6)	rs114558714	7.50E-09	0.0001	0.0057	-	Enhancer	Enhancer
AC006369.2 (0)	rs11893807	1.40E-09	0.0001	0.0057	-	Enhancer	Enhancer
DENND4B (10)	rs59340135	1.90E-12	0.0001	0.0057	-	None	None
SURF6 (1)	rs10751505	1.50E-09	0.0001	0.0057	-	None	None
ZFP57 (0)	rs374317	6.20E-19	0.0001	0.0057	+	None	None
KIAA1841 (29)	rs2441467	7.70E-11	0.0001	0.0057	-	None	None
CPNE1 (1)	rs7267759	2.20E-09	0.0001	0.0057	-	Transcription	Transcription
TCL6 (7)	rs6575528	3.50E-09	0.0001	0.0057	-	None	None
CTC-281B15.1 (5)	rs11739833	1.80E-10	0.0001	0.0057	-	None	None
DCUN1D4 (3)	rs225172	5.20E-08	0.0001	0.0057	-	Enhancer	Transcription
RPSA (0)	rs36082176	3.80E-15	0.0001	0.0057	-	None	None
RP11-495P10.2 (2)	rs543163	3.30E-09	0.0001	0.0057	-	Promoter	Repressed
LMO7 (2)	rs1951832	1.60E-14	0.0001	0.0057	-	None	None
NUTM2B (6)	rs1617662	6.70E-10	0.0001	0.0057	-	None	None
TMEM14A (4)	rs144733774	9.20E-08	0.0001	0.0057	-	None	None
PDCD6 (0)	rs7719171	6.70E-09	0.0001	0.0057	-	Promoter	Enhancer
PMM2 (3)	rs2304471	1.10E-28	0.0001	0.0057	+	Enhancer	Transcription
CPSF2 (15)	rs61976561	4.80E-10	0.0001	0.0057	-	Promoter	Promoter
VEZF1 (1)	rs8066251	7.60E-12	0.0001	0.0057	-	Transcription	Transcription
GTF2I (26)	rs236671	3.60E-08	0.0001	0.0057	+	Transcription	Transcription

MSMB (4)	rs10826181	1.00E-08	0.0001	0.0057	+	None	None
NBPF12 (77)	rs12070653	7.20E-11	0.0001	0.0057	+	None	None
HLA-DMA (5)	rs115334078	8.00E-16	0.0001	0.0057	+	Enhancer	Transcription
TRAF2 (3)	rs28453782	8.10E-08	0.0001	0.0057	+	None	None
NIN (0)	rs2984268	3.60E-07	0.0001	0.0057	-	None	Transcription
RP1-90J20.12 (0)	rs13193229	5.60E-22	0.0001	0.0057	+	None	Promoter
CRIPAK (0)	rs113727613	3.00E-14	0.0001	0.0057	+	None	Enhancer
MTG2 (2)	rs2151511	7.50E-15	0.0001	0.0057	+	Transcription	Transcription
NPIPA5 (0)	rs3198697	1.80E-15	0.0001	0.0057	+	Transcription	Transcription
NDUFA12 (4)	rs10777662	3.20E-08	0.0001	0.0057	+	None	Enhancer
SNAP29 (1)	rs113543516	2.10E-08	0.0001	0.0057	+	Enhancer	Enhancer
RP11-345P4.10 (1)	rs79113395	1.00E-18	0.0001	0.0057	-	Promoter	Promoter
RP11-195E2.4 (0)	rs10058698	1.00E-10	0.0001	0.0057	-	None	None
RPL9 (2)	rs1458255	1.60E-26	0.0001	0.0057	-	Enhancer	Enhancer
ZNF419 (0)	rs10853897	5.60E-10	0.0001	0.0057	-	None	None
RP11-624D11.2 (0)	rs12578061	3.50E-10	0.0001	0.0057	+	None	None
SEPT14 (0)	rs138032833	5.90E-09	0.0001	0.0057	+	None	None
CTD-3157E16.1 (1)	rs73272187	8.50E-14	0.0001	0.0057	-	None	None
ITGAV (5)	rs7575516	4.50E-07	0.0001	0.0057	-	Transcription	None
RP11-152H18.3 (0)	rs10769942	6.40E-09	0.0001	0.0057	-	None	None
SLC6A1 (15)	rs2675156	2.20E-09	0.0001	0.0057	-	Promoter	Promoter
CTA-29F11.1 (0)	rs5769138	2.80E-13	0.0001	0.0057	-	Enhancer	Enhancer
WDR18 (10)	rs34402804	2.50E-09	0.0001	0.0057	-	Promoter	Promoter
EFCAB2 (0)	rs199596521	4.80E-09	0.0001	0.0057	+	None	None
RP11-166B2.1 (0)	rs393329	1.40E-11	0.0001	0.0057	-	Promoter	Promoter
RP11-692N5.1 (2)	rs9807368	5.50E-10	0.0001	0.0057	-	None	None
ACTR8 (0)	rs3733075	8.30E-15	0.0001	0.0057	-	None	Transcription
ULBP1 (3)	rs17748069	1.40E-07	0.0001	0.0057	+	None	None
EI24 (13)	rs501064	3.50E-09	0.0001	0.0057	+	None	None
CTD-2651B20.3 (0)	rs12899942	1.40E-17	0.0001	0.0057	+	Enhancer	Enhancer
TMEM220 (2)	rs11078858	2.20E-12	0.0001	0.0057	+	None	None
TRIP10 (15)	rs62106961	2.20E-09	0.0001	0.0057	-	None	None
TMEM61 (0)	rs2495517	2.50E-19	0.0001	0.0057	-	Enhancer	Enhancer
CRHR1 (1)	rs117149993	2.60E-13	0.0001	0.0057	+	None	None
RBM23 (1)	rs3751498	2.00E-11	0.0001	0.0057	+	Promoter	Promoter
THAP9-AS1 (3)	rs55818134	1.20E-07	0.0001	0.0057	-	Transcription	Transcription
RP11-738E22.3 (0)	rs34937594	1.50E-08	0.0001	0.0057	-	None	None
RPS2 (2)	rs397435	7.80E-16	0.0001	0.0057	-	Promoter	Promoter
RPL30 (8)	rs56137923	3.10E-10	0.0001	0.0057	+	Enhancer	None
PDCD6IP (16)	rs7622118	2.80E-11	0.0001	0.0057	-	Transcription	None
CERS5 (1)	rs7968119	9.50E-10	0.0001	0.0057	-	Enhancer	Enhancer
METTL4 (8)	rs11080959	1.30E-09	0.0001	0.0057	+	None	None
USH2A (67)	rs111917618	8.50E-08	0.0001	0.0057	+	None	None
SERHL (3)	rs136979	9.40E-08	0.0001	0.0057	+	None	None
VAMP4 (7)	rs11801283	1.20E-13	0.0001	0.0057	-	None	None
RP1-40E16.12 (0)	rs12663589	2.40E-11	0.0001	0.0057	-	Promoter	Promoter
MAP2K3 (3)	rs17772089	3.60E-19	0.0001	0.0057	+	Enhancer	Enhancer
OLFM4 (0)	rs7999275	1.50E-09	0.0001	0.0057	-	Repressed	Repressed
AP000688.8 (0)	rs6517325	3.00E-10	0.0001	0.0057	+	None	None
NARS2 (0)	rs10751297	3.20E-11	0.0001	0.0057	+	Enhancer	None
SNHG17 (0)	rs1739653	7.00E-13	0.0001	0.0057	-	Transcription	Transcription
RP11-798G7.6 (1)	rs117561912	1.50E-15	0.0001	0.0057	-	Enhancer	Enhancer
RP9 (0)	rs17469596	8.10E-09	0.0001	0.0057	+	None	None
TMX4 (8)	rs6140505	9.20E-08	0.0001	0.0057	-	None	None
HAGH (6)	rs391546	2.40E-07	0.0001	0.0057	-	None	None
HLA-DRB1 (0)	rs9270838	3.20E-37	0.0001	0.0057	-	None	None
DAP (2)	rs267962	4.70E-08	0.0001	0.0057	-	Enhancer	Enhancer
MGST3 (2)	rs9333378	2.30E-22	0.0001	0.0057	+	Enhancer	Enhancer
PMS2 (0)	rs7804542	9.20E-10	0.0001	0.0057	+	Transcription	None
DAGLA (8)	rs10792317	1.80E-07	0.0001	0.0057	+	Enhancer	None
ZNF568 (11)	rs7254245	3.70E-14	0.0001	0.0057	+	Enhancer	None
PIEZO1 (13)	rs35159887	1.50E-21	0.0001	0.0057	-	None	None
PRDX1 (5)	rs11211138	1.00E-10	0.0001	0.0057	-	None	None
SERPINB1 (7)	rs316346	1.70E-10	0.0001	0.0057	-	Promoter	Promoter
DDX11 (0)	rs2543246	1.60E-17	0.0001	0.0057	-	None	None
CTD-2571L23.6 (0)	rs76897340	6.30E-12	0.0001	0.0057	+	Promoter	Promoter
HLA-A (2)	rs116387429	1.80E-30	0.0001	0.0057	-	Repressed	Repressed
PBX3 (9)	rs2149992	1.80E-10	0.0001	0.0057	-	None	None
CCT7 (1)	rs34960845	3.90E-15	0.0001	0.0057	+	None	None
CACNB2 (14)	rs117959874	1.50E-08	0.0001	0.0057	-	None	None
TMEM229B (9)	rs3818242	5.60E-09	0.0001	0.0057	-	None	None
RP11-867G23.8 (4)	rs10896111	1.20E-09	0.0001	0.0057	-	Promoter	Promoter
SCMH1 (4)	rs2268681	9.60E-09	0.0001	0.0057	-	Transcription	Transcription
AGAP7 (0)	rs10761618	8.80E-13	0.0001	0.0057	+	Enhancer	Enhancer
TDRD5 (15)	rs1928003	1.50E-08	0.0001	0.0057	-	None	None
NSUN2 (4)	rs13164691	2.80E-09	0.0001	0.0057	+	Transcription	Transcription
RP1-63G5.5 (0)	rs9619674	1.20E-12	0.0001	0.0057	-	Enhancer	Enhancer
RP11-535M15.1 (1)	rs10122404	8.80E-12	0.0001	0.0057	-	None	None
NFYC (8)	rs593634	5.40E-10	0.0001	0.0057	+	Repressed	None
PTTG1IP (6)	rs189287	1.40E-13	0.0001	0.0057	+	Enhancer	Enhancer
CTD-3080P12.3 (0)	rs13180948	3.20E-08	0.0001	0.0057	+	Enhancer	Enhancer
CSNK1G1 (0)	rs55953000	8.00E-12	0.0001	0.0057	-	Enhancer	Enhancer
HERC2 (57)	rs6497293	9.80E-08	0.0001	0.0057	-	Transcription	Transcription
CBS (15)	rs234709	4.10E-10	0.0001	0.0057	-	None	None
STAT6 (0)	rs703817	7.60E-08	0.0001	0.0057	+	Enhancer	None
C15orf57 (1)	rs62019879	2.70E-17	0.0001	0.0057	-	None	None
MRC1 (26)	rs690928	2.80E-21	0.0001	0.0057	-	None	None
MMD (6)	rs28414540	7.30E-16	0.0001	0.0057	+	Enhancer	Promoter
C14orf79 (0)	rs2582574	7.40E-09	0.0001	0.0057	+	None	Enhancer
RP11-98I9.4 (1)	rs4144163	9.70E-22	0.0001	0.0057	+	None	None
EXOSC6 (0)	rs12102803	6.50E-14	0.0001	0.0057	-	Transcription	Transcription
RP4-564F22.5 (0)	rs1780636	6.20E-08	0.0001	0.0057	-	Transcription	Transcription
POLR1D (0)	rs577504	1.40E-09	0.0001	0.0057	-	Enhancer	None
ITGB3BP (0)	rs11804843	1.70E-21	0.0001	0.0057	+	None	None

AC007966.1 (0)	rs60341479	2.00E-10	0.0001	0.0057	+	None	None
LYSMD4 (0)	rs2603221	3.80E-13	0.0001	0.0057	-	None	None
RP11-156P1.3 (13)	rs139544269	1.80E-20	0.0001	0.0057	+	None	None
PSMD5-AS1 (1)	rs13290413	1.40E-14	0.0001	0.0057	-	None	None
POLD3 (2)	rs61901537	2.80E-08	0.0001	0.0057	+	Transcription	None
DDX42 (0)	rs8075277	2.20E-12	0.0001	0.0057	-	None	None
ERICH1 (3)	rs11775746	4.40E-11	0.0001	0.0057	-	None	None
C6orf141 (5)	rs13220374	2.30E-10	0.0001	0.0057	-	None	None
SIRPA (3)	rs68178619	2.60E-32	0.0001	0.0057	-	None	None
SPHKAP (5)	rs2141334	4.10E-08	0.0001	0.0057	+	None	None
KHDC1 (3)	rs12154185	3.50E-15	0.0001	0.0057	-	None	None
SRP9 (2)	rs61849057	3.20E-08	0.0001	0.0057	-	Transcription	Transcription
HDHD2 (2)	rs74346439	1.70E-10	0.0001	0.0057	+	Promoter	Promoter
CDK7 (2)	rs2069433	5.80E-23	0.0001	0.0057	+	None	None
RP4-625H18.2 (0)	rs1155102	4.90E-11	0.0001	0.0057	-	Enhancer	Enhancer
IFITM2 (1)	rs1059091	8.30E-11	0.0001	0.0057	-	Enhancer	Promoter
ST20 (1)	rs8024713	4.50E-12	0.0001	0.0057	-	Transcription	Promoter
WDYHV1 (4)	rs4006521	1.40E-08	0.0001	0.0057	-	None	None
RP11-69E11.4 (0)	rs1727098	3.30E-13	0.0001	0.0057	-	Transcription	None
XKR8 (2)	rs4908384	2.00E-13	0.0001	0.0057	+	None	None
OSGIN2 (3)	rs2735376	1.30E-07	0.0001	0.0057	+	Enhancer	Enhancer
SLC44A3 (10)	rs1782396	2.90E-10	0.0001	0.0057	+	None	None
RP13-262C2.3 (1)	rs75822939	1.30E-09	0.0001	0.0057	+	None	None
RP11-277L2.4 (0)	rs55803744	3.20E-10	0.0001	0.0057	-	None	None
PTCHD3P1 (4)	rs3740005	1.90E-08	0.0001	0.0057	-	Transcription	None
HAPLN3 (5)	rs7178493	7.60E-14	0.0001	0.0057	-	Repressed	Repressed
PLD1 (31)	rs4243418	9.40E-08	0.0001	0.0057	-	None	Enhancer
EMC9 (1)	rs2236352	4.20E-09	0.0001	0.0057	-	Promoter	Promoter
C15orf40 (1)	rs57779835	2.00E-11	0.0001	0.0057	+	Transcription	None
MPHOSPH6 (4)	rs2911390	1.30E-09	0.0001	0.0057	-	Repressed	None
AREGB (0)	rs79077380	1.20E-16	0.0001	0.0057	+	None	None
COL9A2 (2)	rs55674553	4.90E-08	0.0001	0.0057	+	Transcription	None
TCTN2 (9)	rs786430	6.90E-10	0.0001	0.0057	-	Enhancer	Promoter
PRKCD (5)	rs11130347	3.10E-22	0.0001	0.0057	-	Enhancer	Enhancer
GOLGA8A (1)	rs4923983	8.10E-11	0.0001	0.0057	+	None	None
CBWD1 (12)	rs116619175	5.30E-12	0.0001	0.0057	-	None	None
OR2L13 (2)	rs61857507	1.70E-07	0.0001	0.0057	+	Promoter	Promoter
BRI3 (3)	rs11761206	2.70E-17	0.0001	0.0057	-	Enhancer	None
RP11-111A22.1 (4)	rs11632061	2.30E-10	0.0001	0.0057	-	Transcription	None
PHF2 (20)	rs7848336	7.70E-13	0.0001	0.0057	+	None	None
IL1R1 (8)	rs1558642	3.20E-18	0.0001	0.0057	+	Transcription	Promoter
WIPI1 (11)	rs2909210	1.20E-09	0.0001	0.0057	-	Enhancer	Enhancer
EFCAB1 (4)	rs3750259	4.40E-11	0.0001	0.0057	-	Promoter	Promoter
DCAKD (0)	rs112106588	5.90E-09	0.0001	0.0057	+	None	None
ACCS (13)	rs11037854	2.20E-08	0.0001	0.0057	+	None	None
LRRC37A (0)	rs17698176	9.70E-10	0.0001	0.0057	+	None	None
CPSF1 (0)	rs34247131	3.60E-14	0.0001	0.0057	-	None	None
ERGIC1 (3)	rs564109	5.60E-10	0.0001	0.0057	-	Enhancer	Enhancer
ATXN1 (3)	rs79906303	4.40E-13	0.0001	0.0057	-	Enhancer	Enhancer
NCOA3 (21)	rs387249	1.20E-10	0.0001	0.0057	-	Transcription	Transcription
IRX4 (0)	rs7448071	1.00E-19	0.0001	0.0057	-	None	None
LRRC61 (1)	rs59211922	1.50E-14	0.0001	0.0057	+	Transcription	Transcription
RBM39 (24)	rs2425183	2.80E-18	0.0001	0.0057	-	None	None
RP11-63P12.7 (0)	rs2208744	1.40E-21	0.0001	0.0057	+	Transcription	Transcription
NT5DC2 (13)	rs62256903	1.30E-08	0.0001	0.0057	-	Enhancer	None
AP001625.6 (1)	rs1878069	1.20E-08	0.0001	0.0057	-	Enhancer	Enhancer
FAM66D (0)	rs140730217	4.30E-08	0.0001	0.0057	-	None	None
ERAP2 (1)	rs2927608	9.60E-26	0.0001	0.0057	+	None	None
ZNF155 (3)	rs391267	1.50E-10	0.0001	0.0057	+	Transcription	Transcription
CDC16 (10)	rs9590507	5.10E-08	0.0001	0.0057	+	Transcription	Transcription
RTCA (12)	rs4364902	2.10E-11	0.0001	0.0057	-	None	Enhancer
HPS1 (17)	rs11599112	3.00E-12	0.0001	0.0057	-	Transcription	Transcription
ASPH (34)	rs3829051	1.40E-08	0.0001	0.0057	-	Promoter	Promoter
C17orf100 (0)	rs9913016	2.90E-13	0.0001	0.0057	-	Enhancer	Promoter
KIAA1586 (0)	rs9885757	3.90E-07	0.0001	0.0057	-	Transcription	None
C3orf20 (0)	rs3773489	4.00E-08	0.0001	0.0057	-	Transcription	Transcription
C4BPA (1)	rs8942	3.70E-08	0.0001	0.0057	+	None	None
RFWD3 (12)	rs9931225	7.50E-12	0.0001	0.0057	-	Enhancer	Enhancer
NBPF10 (14)	rs833399	5.10E-09	0.0001	0.0057	-	None	None
RP11-453E17.1 (1)	rs9991141	6.50E-12	0.0001	0.0057	+	Transcription	Transcription
KLF5 (5)	rs73220641	3.50E-20	0.0001	0.0057	+	Promoter	Promoter
CATSPER2 (3)	rs78282429	2.80E-07	0.0001	0.0057	+	None	None
TNKS2-AS1 (1)	rs4403720	1.20E-09	0.0001	0.0057	-	None	None
C6orf57 (3)	rs1775338	7.20E-14	0.0001	0.0057	-	Transcription	None
CHMP2B (5)	rs12492392	2.70E-07	0.0001	0.0057	+	None	None
AC009299.3 (0)	rs11678980	6.90E-33	0.0001	0.0057	-	Promoter	Promoter
RP11-392P7.6 (11)	rs735689	2.30E-13	0.0001	0.0057	+	Insulator	Insulator
ZNF124 (0)	rs10924924	2.30E-23	0.0001	0.0057	-	None	None
ZFAS1 (0)	rs238170	5.50E-12	0.0001	0.0057	-	Enhancer	Transcription
RP5-935K16.1 (0)	rs9630959	3.30E-09	0.0001	0.0057	-	Transcription	Transcription
SP140L (15)	rs141046703	1.40E-10	0.0001	0.0057	-	None	None
SEC14L6 (6)	rs35340872	6.10E-09	0.0001	0.0057	-	Repressed	Repressed
LARGE (0)	rs7287612	9.60E-08	0.0001	0.0057	+	None	None
ARHGEF5 (0)	rs2091645	2.20E-13	0.0001	0.0057	+	None	None
DHRS4-AS1 (2)	rs10148823	4.20E-10	0.0001	0.0057	-	Enhancer	Enhancer
DUSP5 (3)	rs17127481	1.10E-10	0.0001	0.0057	-	Transcription	Transcription
MDGA1 (0)	rs6909255	9.00E-08	0.0001	0.0057	-	None	None
FSTL5 (10)	rs58191625	5.90E-09	0.0001	0.0057	+	None	None
AMH (0)	rs733846	5.10E-08	0.0001	0.0057	+	Enhancer	Enhancer
MPZL1 (9)	rs11807657	1.90E-07	0.0001	0.0057	+	Transcription	Transcription
ETFA (16)	rs2456049	3.90E-13	0.0001	0.0057	+	Repressed	Repressed
MAP1LC3A (5)	rs57654090	4.30E-11	0.0001	0.0057	-	None	None
TCEB3C (0)	rs2956975	3.40E-12	0.0001	0.0057	+	None	None
PARP11 (3)	rs7975273	4.10E-08	0.0001	0.0057	-	Transcription	Transcription

FAM118A (10)	rs738177	6.20E-11	0.0001	0.0057	-	Transcription	Enhancer
ANP32E (2)	rs7532164	1.90E-17	0.0001	0.0057	-	None	None
ACSL3 (3)	rs10469670	1.20E-08	0.0001	0.0057	-	Enhancer	Enhancer
ARAP1 (37)	rs59400415	1.10E-08	0.0001	0.0057	-	Enhancer	Enhancer
TMEM242 (3)	rs151041972	4.90E-08	0.0001	0.0057	-	None	None
YEATS4 (0)	rs11177615	4.80E-08	0.0001	0.0057	-	Promoter	Promoter
MAP6D1 (0)	rs2255015	3.80E-14	0.0001	0.0057	-	Transcription	Transcription
FKBP10 (11)	rs60588087	5.10E-14	0.0001	0.0057	-	Transcription	None
RARRES2 (0)	rs3800780	8.00E-08	0.0001	0.0057	-	Transcription	Transcription
RALY (10)	rs6088361	1.30E-10	0.0001	0.0057	-	None	None
PRRC2A (11)	rs114736343	5.60E-09	0.0001	0.0057	-	Promoter	Promoter
TRMT61B (4)	rs6718662	1.90E-09	0.0001	0.0057	-	None	None
FAM3B (1)	rs66817580	3.60E-08	0.0001	0.0057	+	Promoter	Promoter
PKD1 (20)	rs9938566	5.00E-10	0.0001	0.0057	-	None	None
SURF1 (5)	rs3124767	2.80E-10	0.0001	0.0057	-	None	None
HCG17 (4)	rs2516698	5.90E-10	0.0001	0.0057	+	None	None
SFXN4 (10)	rs3740561	1.30E-15	0.0001	0.0057	-	None	None
BAG6 (0)	rs139213951	5.40E-33	0.0001	0.0057	-	None	None
UVSSA (0)	rs113727613	6.80E-10	0.0001	0.0057	+	None	Enhancer
RP11-420G6.4 (0)	rs318429	2.70E-23	0.0001	0.0057	-	Enhancer	Promoter
WDR70 (11)	rs7703775	1.30E-08	0.0001	0.0057	-	None	None
HSP90B1 (17)	rs3794239	5.10E-09	0.0001	0.0057	-	Enhancer	Promoter
MPPE1 (14)	rs8097892	4.20E-26	0.0001	0.0057	-	Enhancer	Enhancer
SNHG8 (1)	rs788646	1.10E-16	0.0001	0.0057	+	None	None
WNT4 (3)	rs10917152	1.00E-07	0.0001	0.0057	-	Enhancer	Transcription
RP11-328N19.1 (1)	rs11931505	8.20E-11	0.0001	0.0057	+	None	None
MED4 (3)	rs112665619	1.10E-09	0.0001	0.0057	-	Enhancer	None
FCGBP (14)	rs141933551	1.40E-13	0.0001	0.0057	+	None	None
NAAA (0)	rs11939003	6.10E-11	0.0001	0.0057	+	None	None
THAP7-AS1 (0)	rs370907	6.60E-13	0.0001	0.0057	-	Enhancer	None
GOLGA8Q (1)	rs28528811	2.30E-09	0.0001	0.0057	-	None	None
GOLGA8B (1)	rs148940709	8.40E-24	0.0001	0.0057	-	None	None
ECHDC3 (5)	rs718641	1.20E-12	0.0001	0.0057	+	Enhancer	None
C1QTNF4 (1)	rs10128705	5.40E-11	0.0001	0.0057	-	None	None
SERF1A (6)	rs7443740	5.90E-09	0.0001	0.0057	-	None	None
ZNF365 (0)	rs191135931	1.70E-09	0.0001	0.0057	-	None	None
NQO2 (1)	rs138616686	3.10E-11	0.0001	0.0057	-	None	None
PNMAL1 (0)	rs8107491	5.40E-14	0.0001	0.0057	-	Enhancer	Enhancer
RP11-1260E13.4 (0)	rs9905757	4.10E-08	0.0001	0.0057	+	Enhancer	Enhancer
SNX19 (11)	rs7949722	4.40E-11	0.0001	0.0057	-	Transcription	Transcription
KCND3 (0)	rs72694622	6.60E-08	0.0001	0.0057	+	None	None
ENPP5 (0)	rs66752086	1.10E-09	0.0001	0.0057	-	None	None
ACSM1 (3)	rs6497506	1.40E-09	0.0001	0.0057	+	None	None
SPRR3 (2)	rs78426057	3.20E-10	0.0001	0.0057	-	None	None
RP11-334C17.5 (0)	rs9914372	6.50E-10	0.0001	0.0057	-	None	None
LEKR1 (1)	rs382365	9.90E-13	0.0001	0.0057	-	None	None
NT5C3B (1)	rs4796725	2.60E-38	0.0001	0.0057	-	Enhancer	None
NSA2 (0)	rs113347529	2.70E-14	0.0001	0.0057	+	Transcription	Transcription
SAV1 (4)	rs2934679	7.50E-09	0.0001	0.0057	-	Transcription	None
NR1H3 (1)	rs58405523	2.70E-07	0.0001	0.0057	-	None	None
PIAS1 (3)	rs56106454	3.40E-12	0.0001	0.0057	+	Transcription	Enhancer
RP11-678G14.2 (0)	rs34497957	8.50E-12	0.0001	0.0057	-	Transcription	None
PHLDB1 (1)	rs2077173	3.00E-13	0.0001	0.0057	+	Promoter	Enhancer
LINC00680 (0)	rs6936706	3.50E-08	0.0001	0.0057	-	None	None
TRPV3 (12)	rs322960	6.90E-09	0.0001	0.0057	-	Enhancer	None
POLR2J (0)	rs116535101	3.80E-09	0.0001	0.0057	-	None	None
EPHX1 (2)	rs11808973	1.60E-08	0.0001	0.0057	-	None	None
MRPL36 (2)	rs3776143	6.30E-10	0.0001	0.0057	-	Transcription	Transcription
RGS5 (10)	rs12733197	1.10E-10	0.0001	0.0057	-	None	None
MGAM (63)	rs28429916	5.40E-08	0.0001	0.0057	-	None	None
GOLGA8O (10)	rs12591935	7.80E-13	0.0001	0.0057	+	None	None
EMR2 (15)	rs6511971	2.70E-11	0.0001	0.0057	-	None	Repressed
ITGB2 (13)	rs760462	8.30E-20	0.0001	0.0057	-	Promoter	Promoter
RP11-44F21.5 (0)	rs10001788	5.30E-09	0.0001	0.0057	+	None	None
SCUBE2 (6)	rs11042221	1.20E-07	0.0001	0.0057	+	None	None
L1TD1 (2)	rs2457823	4.40E-11	0.0001	0.0057	+	Repressed	Repressed
ZNF266 (0)	rs6512094	4.80E-18	0.0001	0.0057	+	Transcription	Promoter
RTN4 (0)	rs2588510	5.20E-23	0.0001	0.0057	-	Enhancer	Enhancer
CHRFAM7A (3)	rs12906190	1.90E-11	0.0001	0.0057	+	None	None
CST2 (0)	rs6114290	1.00E-07	0.0001	0.0057	-	None	None
COX14 (2)	rs56151073	9.60E-19	0.0001	0.0057	-	None	Transcription
PDXDC1 (12)	rs62036867	2.80E-09	0.0001	0.0057	-	Enhancer	Enhancer
NEK6 (17)	rs7863470	9.80E-07	0.0001	0.0057	-	None	None
RP11-63P12.6 (0)	rs1109586	2.60E-17	0.0001	0.0057	+	Enhancer	Enhancer
BACE2 (11)	rs914187	2.30E-11	0.0001	0.0057	-	None	Promoter
RRP12 (1)	rs7903847	7.70E-10	0.0001	0.0057	+	Transcription	Transcription
RP11-661A12.9 (4)	rs10093709	5.50E-14	0.0001	0.0057	+	None	None
NCL (11)	rs78097717	3.30E-09	0.0001	0.0057	-	Enhancer	Promoter
SMPD1 (0)	chr11:6411935	7.50E-08	0.0001	0.0057	+	Promoter	Promoter
PHYH (6)	rs631496	9.60E-08	0.0001	0.0057	+	None	None
C19orf47 (6)	rs3745195	9.00E-13	0.0001	0.0057	+	Transcription	None
THNSL2 (10)	rs6547757	8.30E-16	0.0001	0.0057	+	Promoter	Promoter
CTD-2001E22.2 (1)	rs422309	3.70E-10	0.0001	0.0057	+	Promoter	Promoter
WDR52 (0)	rs1552433	5.00E-14	0.0001	0.0057	-	None	None
YY1AP1 (9)	rs12034326	3.40E-07	0.0001	0.0057	+	Promoter	Promoter
MAPKAPK5 (13)	rs117399332	2.60E-07	0.0001	0.0057	-	None	None
MICB (0)	rs147232276	4.80E-10	0.0001	0.0057	-	Repressed	Repressed
DUSP14 (0)	rs865483	7.00E-32	0.0001	0.0057	-	Enhancer	Enhancer
SEC22A (6)	rs7638386	2.90E-09	0.0001	0.0057	-	Transcription	Transcription
GTSF1 (6)	rs12307149	4.30E-13	0.0001	0.0057	+	Repressed	Enhancer
LCMT1 (1)	rs12917728	7.90E-17	0.0001	0.0057	+	None	None
SAT2 (2)	rs858520	5.90E-17	0.0001	0.0057	-	Enhancer	Enhancer
ZNF561 (1)	rs34357705	1.30E-09	0.0001	0.0057	-	Transcription	Transcription
CEP70 (0)	rs774008	3.40E-09	0.0001	0.0057	+	None	Repressed

ANKRD40 (0)	rs2240802	2.90E-08	0.0001	0.0057	-	None	None
FLYWCH1 (0)	rs7193548	6.00E-19	0.0001	0.0057	-	None	None
CIB2 (0)	rs11547207	3.00E-08	0.0001	0.0057	+	Promoter	Promoter
NOD1 (14)	rs2529440	3.10E-08	0.0001	0.0057	-	Enhancer	Enhancer
AGAP6 (0)	rs11599257	9.50E-12	0.0001	0.0057	-	None	Transcription
NOL10 (0)	rs56403646	2.90E-09	0.0001	0.0057	-	None	None
MRI1 (1)	rs455093	7.20E-13	0.0001	0.0057	-	Promoter	Promoter
CEP68 (3)	rs2723065	1.60E-09	0.0001	0.0057	-	None	None
PTPN20A (0)	rs56036703	2.80E-15	0.0001	0.0057	+	None	None
RP11-996F15.2 (0)	rs11050175	1.70E-12	0.0001	0.0057	+	None	None
GSDMD (0)	rs12679598	4.80E-21	0.0001	0.0057	-	None	None
RP11-660L16.2 (0)	rs11606612	3.30E-09	0.0001	0.0057	+	Promoter	Promoter
UBE2U (2)	rs11208378	1.20E-07	0.0001	0.0057	+	None	None
SDCCAG8 (21)	rs10927025	9.70E-09	0.0001	0.0057	+	Enhancer	Enhancer
FAM153A (15)	rs62398521	1.20E-08	0.0001	0.0057	+	None	None
PLCG2 (15)	rs62046430	2.80E-10	0.0001	0.0057	+	Promoter	Promoter
ACOT2 (4)	rs8015976	8.80E-22	0.0001	0.0057	-	None	None
GPR156 (0)	rs1700	2.30E-09	0.0001	0.0057	+	Transcription	None
RP11-286H14.6 (0)	rs202243211	3.10E-10	0.0001	0.0057	-	None	None
RAET1G (2)	rs9322225	3.90E-08	0.0001	0.0057	+	Promoter	Promoter
AC005154.6 (1)	rs2270007	2.00E-13	0.0001	0.0057	-	None	None
LIMS1 (15)	rs12476399	5.00E-22	0.0001	0.0057	-	None	None
BTD (1)	rs2174267	3.20E-09	0.0001	0.0057	+	None	None
NAB1 (5)	rs2293765	7.40E-24	0.0001	0.0057	-	Enhancer	Enhancer
CD151 (1)	rs28636471	1.30E-16	0.0001	0.0057	+	Enhancer	Promoter
AC005534.9 (1)	rs2302145	4.50E-08	0.0001	0.0057	-	Transcription	Transcription
C4orf33 (7)	rs139037552	1.20E-10	0.0001	0.0057	-	None	None
AC104695.3 (1)	rs10176110	7.10E-08	0.0001	0.0057	+	None	Enhancer
RP11-826N14.2 (2)	rs7724125	1.10E-08	0.0001	0.0057	+	Repressed	None
APPL2 (22)	rs1196868	1.30E-09	0.0001	0.0057	+	Enhancer	Enhancer
MFHAS1 (1)	rs13254267	4.00E-08	0.0001	0.0057	-	None	None
LINC00665 (0)	rs2972632	1.40E-16	0.0001	0.0057	-	Transcription	None
RP11-17M24.1 (0)	rs57932203	1.70E-08	0.0001	0.0057	+	Enhancer	Enhancer
RP1-60O19.1 (0)	rs3127633	3.80E-10	0.0001	0.0057	+	None	Enhancer
B3GALTL (16)	rs9544974	1.70E-11	0.0001	0.0057	-	None	None
SDF4 (8)	rs190796582	4.20E-10	0.0001	0.0057	-	Enhancer	Promoter
AP000769.1 (0)	rs1111934	9.20E-09	0.0001	0.0057	+	None	None
ATP5A1 (10)	rs11664764	2.40E-15	0.0001	0.0057	+	Enhancer	Transcription
RCN1 (12)	rs223049	9.90E-08	0.0001	0.0057	-	None	None
CALCOCO2 (2)	rs35105713	2.80E-12	0.0001	0.0057	+	Enhancer	None
ZNF584 (1)	rs7260571	1.30E-08	0.0001	0.0057	-	Transcription	None
PRKAR1B (15)	rs4072186	5.70E-09	0.0001	0.0057	+	None	Enhancer
GABPB1-AS1 (1)	rs7179935	2.80E-08	0.0001	0.0057	+	None	None
CCT3 (9)	rs4445457	3.50E-22	0.0001	0.0057	+	Transcription	Transcription
HADHB (1)	rs6761770	1.30E-16	0.0001	0.0057	-	Enhancer	Enhancer
HSP90AB1 (1)	rs12204119	1.20E-08	0.0001	0.0057	-	None	None
SH3YL1 (1)	rs59937473	8.90E-18	0.0001	0.0057	-	None	Enhancer
SYCE3 (0)	rs79948724	4.80E-09	0.0001	0.0057	+	None	None
SRP72 (1)	rs12650611	6.60E-08	0.0001	0.0057	-	None	None
CMC2 (5)	rs11150334	1.60E-23	0.0001	0.0057	+	None	None
TUFM (0)	rs42861	8.10E-07	0.0001	0.0057	+	None	None
ZNF773 (3)	rs4316866	2.70E-10	0.0001	0.0057	-	Transcription	None
SNHG5 (3)	rs1059307	3.60E-28	0.0001	0.0057	+	Promoter	Promoter
TMEM45A (2)	rs7619507	4.20E-10	0.0001	0.0057	+	Promoter	Promoter
ITPA (1)	rs6515776	1.80E-09	0.0001	0.0057	-	Transcription	Transcription
HTT (58)	rs362307	3.00E-09	0.0001	0.0057	-	Enhancer	Enhancer
PXK (7)	rs67990985	1.70E-07	0.0001	0.0057	-	None	None
GTF2B (4)	rs6691335	2.00E-07	0.0001	0.0057	+	Repressed	None
SNRNP25 (4)	rs216599	2.70E-08	0.0001	0.0057	-	Repressed	Repressed
CYP3A5 (10)	rs10238028	1.90E-07	0.0001	0.0057	+	None	None
C1orf63 (11)	rs72660908	2.50E-12	0.0001	0.0057	-	None	None
ADHFE1 (13)	rs2555582	1.60E-09	0.0001	0.0057	+	None	Transcription
NTPCR (2)	rs12743233	4.20E-28	0.0001	0.0057	-	Transcription	Enhancer
RASGRP3 (19)	rs113252370	1.10E-11	0.0001	0.0057	-	None	None
ZNF100 (0)	rs12972593	8.60E-18	0.0001	0.0057	-	None	Transcription
NUP50 (3)	rs2673084	1.50E-11	0.0001	0.0057	+	Transcription	Transcription
ATP5SL (1)	rs3786553	2.50E-15	0.0001	0.0057	-	Promoter	Promoter
BAIAP2L2 (2)	rs132929	9.80E-14	0.0001	0.0057	-	None	None
MOK (16)	rs76141924	9.30E-08	0.0001	0.0057	-	None	None
RP1-90G24.6 (1)	rs9619241	8.20E-08	0.0001	0.0057	+	None	None
TDRD9 (13)	rs1980619	1.30E-08	0.0001	0.0057	-	Enhancer	Enhancer
CTC-297N7.5 (0)	rs202311	6.60E-10	0.0001	0.0057	+	Promoter	Promoter
SNRPC (1)	rs35839188	1.50E-08	0.0001	0.0057	-	None	None
MPRIP (7)	rs11867506	3.60E-17	0.0001	0.0057	-	Transcription	Transcription
FIGNL1 (1)	rs12533940	2.30E-08	0.0001	0.0057	+	Repressed	Repressed
ZNF528 (10)	rs1824245	5.70E-11	0.0001	0.0057	+	Transcription	Promoter
MED24 (0)	rs10852937	3.00E-08	0.0001	0.0057	-	Repressed	Repressed
CANT1 (3)	rs2377301	4.20E-09	0.0001	0.0057	-	Transcription	None
CTB-5E10.3 (0)	rs62111896	1.20E-13	0.0001	0.0057	-	Promoter	Promoter
MTRF1L (6)	rs6917114	1.30E-07	0.0001	0.0057	-	Transcription	None
ZNF550 (0)	rs73060407	8.30E-10	0.0001	0.0057	+	Transcription	Promoter
POMZP3 (0)	rs147601722	3.10E-10	0.0001	0.0057	-	None	None
NDUFS5 (0)	rs10888639	5.30E-27	0.0001	0.0057	-	Transcription	Transcription
IGSF22 (1)	rs2289965	1.10E-12	0.0001	0.0057	+	Enhancer	Promoter
ZNF232 (2)	rs56152541	3.90E-16	0.0001	0.0057	-	Promoter	Promoter
DCTN5 (6)	rs450787	4.00E-08	0.0001	0.0057	+	Transcription	Transcription
CDH15 (11)	rs55694921	7.10E-09	0.0001	0.0057	+	None	None
DHX40 (11)	rs9909087	4.90E-10	0.0001	0.0057	-	Enhancer	None
SENP1 (18)	rs10747529	3.60E-13	0.0001	0.0057	+	Transcription	Transcription
FPGT (1)	rs11210395	5.10E-07	0.0001	0.0057	-	None	None
GSTT2 (0)	rs5751775	7.80E-16	0.0001	0.0057	-	Transcription	Transcription
ISCU (0)	rs10778647	8.70E-09	0.0001	0.0057	-	Promoter	Promoter
CARD10 (18)	rs111533522	1.80E-08	0.0001	0.0057	-	Enhancer	Enhancer
AC016683.6 (10)	rs1139015	1.70E-11	0.0001	0.0057	-	Enhancer	Enhancer

CGNL1 (7)	rs878958	1.60E-23	0.0001	0.0057	+	None	None
DYM (21)	rs140429629	1.70E-11	0.0001	0.0057	-	None	None
ZNF408 (3)	rs112181005	9.20E-09	0.0001	0.0057	-	None	None
NUP85 (14)	rs1478785	1.10E-07	0.0001	0.0057	-	Transcription	Transcription
TPMT (4)	rs9465105	5.50E-08	0.0001	0.0057	-	Enhancer	Transcription
AC104135.3 (0)	rs12366	4.10E-13	0.0001	0.0057	+	Promoter	Promoter
RP11-430H10.1 (0)	rs12418562	1.40E-08	0.0001	0.0057	+	None	None
MEPCE (4)	rs11771419	1.40E-08	0.0001	0.0057	-	None	None
RP11-364B6.1 (0)	rs11185368	2.90E-09	0.0001	0.0057	+	None	None
CTD-2008A1.1 (1)	rs111227851	1.60E-10	0.0001	0.0057	-	None	None
AC009299.2 (0)	rs11678980	4.30E-11	0.0001	0.0057	-	Promoter	Promoter
ZG16B (0)	rs7190558	1.60E-08	0.0001	0.0057	+	Repressed	Repressed
LPP (8)	rs6787621	7.80E-09	0.0001	0.0057	-	Enhancer	Enhancer
ISX (0)	rs117258550	3.20E-08	0.0001	0.0057	+	None	None
PEX6 (0)	rs1053539	1.40E-13	0.0001	0.0057	+	Transcription	Transcription
REEP4 (0)	rs11550100	8.50E-09	0.0001	0.0057	-	Enhancer	Enhancer
APBB2 (20)	rs2880169	1.20E-09	0.0001	0.0057	-	None	None
MOCS2 (6)	rs2052568	6.10E-12	0.0001	0.0057	-	None	None
ELP2 (7)	rs1632169	6.50E-15	0.0001	0.0057	-	Promoter	Promoter
HEPHL1 (4)	rs1138800	3.50E-10	0.0001	0.0057	+	Promoter	Promoter
RP11-95I16.4 (1)	rs12784536	5.60E-09	0.0001	0.0057	-	None	Repressed
AP000350.4 (0)	rs5751771	2.70E-14	0.0001	0.0057	+	None	None
OR7D2 (0)	rs73494602	6.00E-19	0.0001	0.0057	+	Transcription	Transcription
BOP1 (3)	rs4977199	4.60E-09	0.0001	0.0057	-	None	None
STYXL1 (2)	rs150685585	2.60E-08	0.0001	0.0057	+	Transcription	None
NUDT9 (0)	rs9995987	1.20E-10	0.0001	0.0057	-	None	Enhancer
NINL (7)	rs6115118	8.10E-08	0.0001	0.0057	-	None	None
DLEU1 (1)	rs9596240	7.40E-08	0.0001	0.0057	-	None	None
HOTAIRM1 (2)	rs1008410	1.60E-12	0.0001	0.0057	-	Promoter	Promoter
CLPSL1 (2)	rs9380535	2.80E-08	0.0001	0.0057	+	Repressed	Repressed
CYP2D6 (1)	rs5758574	1.20E-08	0.0001	0.0057	+	Transcription	None
ZNF141 (0)	rs10012198	3.40E-16	0.0001	0.0057	-	None	None
INTS4 (12)	rs13377546	9.80E-16	0.0001	0.0057	-	None	Transcription
SLC20A1 (0)	rs17515715	9.10E-16	0.0001	0.0057	-	Promoter	Promoter
GSTA1 (6)	rs6458870	2.90E-11	0.0001	0.0057	-	None	None
RAD51C (0)	rs113510006	3.50E-07	0.0001	0.0057	+	None	None
SDHA (1)	rs10057501	8.30E-15	0.0001	0.0057	+	Transcription	None
MFSD11 (8)	rs2077451	9.60E-08	0.0001	0.0057	+	Repressed	Repressed
NCALD (12)	rs561000	2.30E-13	0.0001	0.0057	+	None	None
SLC9A3 (7)	rs62622783	8.10E-09	0.0001	0.0057	+	None	None
DGCR6 (5)	rs73155293	2.70E-10	0.0001	0.0057	-	Insulator	Insulator
C14orf2 (1)	rs79347800	1.40E-16	0.0001	0.0057	+	None	None
FSHB (2)	rs1222207	1.50E-08	0.0001	0.0057	+	None	None
TMBIM1 (10)	rs10195115	6.20E-10	0.0001	0.0057	+	Enhancer	None
MOB1B (1)	rs11947917	1.60E-08	0.0001	0.0057	-	None	None
RP11-6L6.2 (1)	rs6856647	1.40E-08	0.0001	0.0057	+	None	None
C2orf76 (0)	rs7601849	2.10E-07	0.0001	0.0057	-	Insulator	None
GPR149 (0)	rs74761378	1.00E-10	0.0001	0.0057	+	None	None
LINC00865 (3)	rs12240362	5.10E-11	0.0001	0.0057	+	None	None
AC004012.1 (0)	rs7777014	1.30E-07	0.0001	0.0057	-	None	None
CTB-171A8.1 (2)	rs113670196	1.40E-08	0.0001	0.0057	+	Enhancer	Enhancer
RP11-1060J15.4 (0)	rs144049521	5.70E-08	0.0001	0.0057	+	None	None
FAM86C1 (0)	rs3891415	1.70E-16	0.0001	0.0057	-	None	None
KIF13A (28)	rs10949465	2.90E-10	0.0001	0.0057	-	None	None
POLR1B (19)	rs4849076	5.40E-09	0.0001	0.0057	-	Transcription	Transcription
PTER (5)	rs12265362	1.10E-10	0.0001	0.0057	-	Enhancer	None
TMEM9 (0)	rs1106729	2.80E-10	0.0001	0.0057	+	Promoter	Promoter
LRP2 (58)	rs2601098	3.60E-10	0.0001	0.0057	+	None	None
NBPF9 (8)	rs2798892	1.10E-06	0.0001	0.0057	+	Promoter	Promoter
TFIP11 (13)	rs134136	6.40E-09	0.0001	0.0057	-	Transcription	Transcription
CADPS (31)	rs13096167	2.50E-08	0.0001	0.0057	+	Enhancer	None
RAB11FIP4 (7)	rs2343164	2.60E-08	0.0001	0.0057	-	Enhancer	Enhancer
RP11-845M18.7 (1)	chr12:52685096	5.10E-08	0.0001	0.0057	-	Repressed	None
MAN2A1 (21)	rs11956236	1.80E-26	0.0001	0.0057	-	Enhancer	None
PIGN (0)	rs12607287	3.50E-12	0.0001	0.0057	-	None	None
RP11-498P14.5 (2)	rs10739333	3.40E-08	0.0001	0.0057	+	Repressed	None
TMEM230 (3)	rs6107581	1.10E-08	0.0001	0.0057	-	Enhancer	None
KNSTRN (10)	rs55982218	7.40E-19	0.0001	0.0057	+	Enhancer	Promoter
AC005082.12 (0)	rs1618339	9.60E-13	0.0001	0.0057	+	None	Transcription
TLL2 (13)	rs12570043	8.30E-10	0.0001	0.0057	+	None	None
HAP1 (5)	rs4796601	7.40E-11	0.0001	0.0057	-	None	None
RP11-324I22.4 (0)	rs2474528	8.60E-08	0.0001	0.0057	+	None	Enhancer
DOCK1 (53)	rs2229605	5.60E-09	0.0001	0.0057	-	Transcription	None
FBF1 (22)	rs10852766	4.30E-09	0.0001	0.0057	-	Transcription	Transcription
C2orf54 (0)	rs11680800	5.30E-10	0.0001	0.0057	+	None	None
SIGLEC12 (0)	rs62116417	4.70E-12	0.0001	0.0057	+	Insulator	Insulator
PPM1B (1)	rs7567874	3.10E-13	0.0001	0.0057	-	None	None
SEPT11 (2)	rs2703104	5.40E-08	0.0001	0.0057	+	Enhancer	Enhancer
NT5DC3 (1)	rs2255316	1.60E-08	0.0001	0.0057	-	Enhancer	Enhancer
SPTBN5 (41)	rs1648818	1.30E-07	0.0001	0.0057	-	None	None
UROS (0)	rs10901448	1.60E-08	0.0001	0.0057	-	None	None
XKR9 (0)	rs1993862	5.10E-12	0.0001	0.0057	+	Enhancer	Enhancer
DHRS1 (1)	rs142854562	1.00E-07	0.0001	0.0057	+	None	None
ACOT1 (0)	MERGED_DEL_2_77045_-	1.20E-19	0.0001	0.0057	-	None	None
LINC00476 (3)	rs689860	2.80E-10	0.0001	0.0057	+	Transcription	Enhancer
CTC-308K20.2 (0)	rs28550282	3.80E-10	0.0001	0.0057	-	None	Enhancer
SULT1A1 (2)	rs3986805	5.10E-10	0.0001	0.0057	-	None	None
DR1 (3)	rs3767964	6.90E-08	0.0001	0.0057	-	Enhancer	Promoter
TMEM236 (3)	rs77529815	3.30E-18	0.0001	0.0057	+	None	None
FAM120B (3)	rs9356639	4.30E-09	0.0001	0.0057	-	Enhancer	None
MAN1A1 (13)	rs12205826	1.80E-10	0.0001	0.0057	+	Enhancer	Enhancer
TMEM254-AS1 (2)	rs7080711	2.20E-11	0.0001	0.0057	+	None	None
WNT2 (0)	rs38916	6.10E-08	0.0001	0.0057	+	None	None
WDR11 (14)	rs7904153	1.20E-11	0.0001	0.0057	-	None	None

NCOA6 (2)	rs6088578	1.70E-18	0.0001	0.0057	-	None	None
ZNF502 (3)	rs147882697	1.70E-08	0.0001	0.0057	+	None	None
ABCB9 (2)	rs55742290	4.90E-07	0.0001	0.0057	-	Enhancer	Promoter
TIMM10 (1)	rs2649662	3.00E-12	0.0001	0.0057	-	None	None
DMTN (12)	rs1078363	1.90E-08	0.0001	0.0057	-	None	None
DNAJA3 (3)	rs6500604	6.40E-09	0.0001	0.0057	-	Transcription	Transcription
NDUFB1 (2)	rs61976561	4.90E-15	0.0001	0.0057	-	Promoter	Promoter
TCF25 (19)	rs74542234	4.10E-11	0.0001	0.0057	-	Promoter	Promoter
RBM19 (18)	rs34672079	1.80E-08	0.0001	0.0057	-	Repressed	Repressed
CTD-2020K17.4 (1)	rs1133458	7.40E-18	0.0001	0.0057	-	None	None
CTD-3110H11.1 (1)	rs7179935	1.10E-07	0.0001	0.0057	+	None	None
NEK3 (2)	rs2296351	3.40E-09	0.0001	0.0057	+	Transcription	Transcription
AC007283.5 (0)	rs7582581	8.20E-18	0.0001	0.0057	-	None	Enhancer
VWDE (21)	rs2119141	3.50E-11	0.0001	0.0057	+	Transcription	None
RP11-661P17.1 (5)	rs4420510	3.40E-08	0.0001	0.0057	-	None	None
LINC00662 (4)	rs138217771	9.20E-09	0.0001	0.0057	-	None	None
CEACAM18 (1)	rs62115064	2.00E-14	0.0001	0.0057	+	Repressed	Repressed
TEN1 (4)	rs1135531	4.90E-08	0.0001	0.0057	+	Insulator	Enhancer
GTF2H2 (0)	rs13168712	1.10E-12	0.0001	0.0057	-	None	None
LARS (0)	rs4913061	3.20E-08	0.0001	0.0057	+	Enhancer	Enhancer
C9orf123 (1)	rs72697241	2.40E-10	0.0001	0.0057	-	Promoter	Promoter
ANKRD36 (32)	rs1257050	3.90E-20	0.0001	0.0057	-	None	None
CHAF1A (2)	rs11670503	4.20E-08	0.0001	0.0057	-	Promoter	Promoter
RPS19 (5)	rs930102	1.10E-12	0.0001	0.0057	-	Promoter	Promoter
LHFPL2 (1)	rs2362832	4.90E-14	0.0001	0.0057	-	None	None
IFI27L1 (5)	rs748762	1.40E-15	0.0001	0.0057	+	None	None
RP11-495K9.7 (0)	rs10902428	8.10E-18	0.0001	0.0057	-	None	None
CCDC171 (6)	rs10756745	2.60E-07	0.0001	0.0057	+	None	None
LTBP3 (8)	rs11227227	2.00E-07	0.0001	0.0057	+	Enhancer	Enhancer
SNRPA1 (7)	rs10152348	6.10E-14	0.0001	0.0057	-	Transcription	Transcription
DMRTA2 (0)	rs6682989	6.30E-07	0.0001	0.0057	-	None	None
CYP2C9 (6)	rs4918797	1.50E-09	0.0001	0.0057	+	None	None
TBC1D9B (0)	rs152234	6.10E-09	0.0001	0.0057	+	Promoter	Promoter
ULK4 (0)	rs11708951	3.50E-09	0.0001	0.0057	+	None	None
GBP4 (1)	rs521704	3.40E-10	0.0001	0.0057	-	None	None
PLSCR4 (8)	rs3804649	7.90E-09	0.0001	0.0057	+	Insulator	None
TGOLN2 (0)	rs908304	1.20E-07	0.0001	0.0057	-	Transcription	None
ENDOV (6)	rs34933300	2.20E-08	0.0001	0.0057	-	None	None
SPAG16 (3)	rs13384724	3.20E-11	0.0001	0.0057	-	None	None
NGDN (1)	rs1555413	3.00E-11	0.0001	0.0057	+	Enhancer	None
PDXDC2P (0)	rs28659838	3.80E-26	0.0001	0.0057	-	None	None
MAGI1 (17)	rs73129695	1.70E-09	0.0001	0.0057	-	Transcription	Transcription
WAC-AS1 (0)	rs2807735	7.10E-10	0.0001	0.0057	-	None	Enhancer
PRUNE2 (15)	rs7039528	2.90E-08	0.0001	0.0057	+	Promoter	Enhancer
CTD-3025N20.3 (0)	rs34533611	1.10E-09	0.0001	0.0057	-	None	None
BSND (3)	rs72660515	2.30E-08	0.0001	0.0057	+	None	None
QDPR (1)	rs2597764	1.00E-08	0.0001	0.0057	-	Transcription	Transcription
ZNF793 (1)	rs12979470	4.50E-13	0.0001	0.0057	+	Transcription	Promoter
CD81 (0)	rs2074251	1.40E-10	0.0001	0.0057	-	Enhancer	Enhancer
HSPBAP1 (7)	rs1864382	1.90E-08	0.0001	0.0057	-	Enhancer	Promoter
GPR108 (17)	rs3810222	5.70E-13	0.0001	0.0057	-	Enhancer	Promoter
NUTM2A-AS1 (4)	rs143272997	2.70E-08	0.0001	0.0057	-	None	None
QRSL1 (11)	rs3121445	4.60E-22	0.0001	0.0057	-	None	None
STAU1 (11)	rs6095417	5.00E-09	0.0001	0.0057	+	Transcription	Transcription
MUL1 (0)	rs6700034	1.60E-09	0.0001	0.0057	+	Promoter	Promoter
C18orf25 (4)	rs4574015	2.20E-25	0.0001	0.0057	-	Enhancer	Enhancer
TMED10 (2)	rs10149362	5.20E-27	0.0001	0.0057	+	Transcription	None
PRADC1 (4)	rs1469530	1.60E-26	0.0001	0.0057	-	Enhancer	Enhancer
SAMD3 (18)	rs62431361	2.70E-09	0.0001	0.0057	-	Enhancer	Enhancer
CFLAR (12)	rs10184098	8.20E-11	0.0001	0.0057	-	Transcription	None
FTSJ3 (0)	rs7212196	3.90E-14	0.0001	0.0057	+	Transcription	Transcription
PHF11 (13)	rs7333668	1.40E-10	0.0001	0.0057	-	Transcription	None
ACP2 (0)	rs12222581	4.60E-08	0.0001	0.0057	-	Transcription	Transcription
FLG-AS1 (13)	rs6666097	2.10E-13	0.0001	0.0057	-	None	None
NOTCH2NL (2)	rs7521222	9.80E-08	0.0001	0.0057	-	None	Transcription
SH3BGR (6)	rs2837046	2.20E-19	0.0001	0.0057	-	None	None
KCTD15 (2)	rs3810361	7.70E-18	0.0001	0.0057	-	Enhancer	Enhancer
APIP (0)	rs72930433	2.20E-09	0.0001	0.0057	+	Transcription	Transcription
MUT (0)	rs9473541	3.50E-08	0.0001	0.0057	+	None	None
TSPAN33 (1)	rs202243211	2.80E-09	0.0001	0.0057	-	None	None
ARHGEF12 (8)	rs73004833	1.80E-07	0.0001	0.0057	+	None	None
SETD5-AS1 (1)	rs6443237	1.20E-17	0.0001	0.0057	-	Enhancer	Enhancer
DOCK7 (3)	rs7531579	3.00E-08	0.0001	0.0057	-	None	None
GPR137B (0)	rs76071820	8.90E-08	0.0001	0.0057	+	None	None
RP11-218M22.1 (6)	rs2607920	4.00E-09	0.0001	0.0057	-	None	None
C4orf19 (1)	rs1552021	1.00E-12	0.0001	0.0057	-	Enhancer	Enhancer
WEE2-AS1 (0)	rs12537498	5.50E-12	0.0001	0.0057	+	Transcription	Transcription
ANAPC13 (0)	rs7627726	1.60E-13	0.0001	0.0057	+	Insulator	None
ZP3 (8)	rs79004139	4.20E-11	0.0001	0.0057	+	None	None
PSORS1C2 (0)	rs3094221	3.30E-09	0.0001	0.0057	+	Repressed	Promoter
RPL8 (0)	rs2954682	3.60E-11	0.0001	0.0057	-	Transcription	None
GTPBP10 (5)	rs194505	2.20E-12	0.0001	0.0057	+	Promoter	Promoter
FDFT1 (4)	rs13247997	2.10E-07	0.0001	0.0057	+	Repressed	None
SRP14 (2)	rs1059395	2.60E-12	0.0001	0.0057	+	Promoter	Promoter
NAPRT1 (0)	rs10093709	2.20E-15	0.0001	0.0057	+	None	None
RP11-161I6.2 (0)	rs579336	4.00E-08	0.0001	0.0057	-	Enhancer	None
QSOX2 (1)	rs112770389	1.40E-08	0.0001	0.0057	-	None	None
LSR (10)	rs2267572	7.10E-09	0.0001	0.0057	-	Transcription	Transcription
ST3GAL3 (7)	rs37471	1.70E-07	0.0001	0.0057	-	None	None
DNALI1 (2)	rs11577559	1.60E-13	0.0001	0.0057	-	Transcription	Transcription
FAM106A (0)	rs6502661	1.10E-14	0.0001	0.0057	+	None	None
ZNF85 (4)	rs4809116	2.80E-12	0.0001	0.0057	+	None	None
CTD-3105H18.7 (0)	rs67988101	2.40E-07	0.0001	0.0057	-	Transcription	Transcription
LYSMD3 (1)	rs511393	1.40E-08	0.0001	0.0057	+	None	None

ERAP1 (13)	rs39841	3.10E-08	0.0001	0.0057	-	None	Transcription
SMPDL3B (3)	rs61789695	1.30E-09	0.0001	0.0057	+	None	None
ZNF320 (3)	rs3810115	5.70E-08	0.0001	0.0057	-	Promoter	Promoter
PNP (4)	rs1713427	2.00E-08	0.0001	0.0057	-	None	None
FAM131C (1)	rs9442230	1.60E-10	0.0001	0.0057	-	None	None
MTUS1 (28)	rs7387668	1.00E-16	0.0001	0.0057	-	None	None
ZNF79 (1)	rs7866252	6.30E-12	0.0001	0.0057	-	None	None
ARSA (0)	rs8142033	5.30E-13	0.0001	0.0057	-	None	None
MDH2 (1)	rs113570449	2.80E-11	0.0001	0.0057	+	Transcription	None
NRD1 (30)	rs6691091	1.40E-13	0.0001	0.0057	-	Transcription	Enhancer
ELFN2 (0)	rs7286065	4.70E-28	0.0001	0.0057	-	Enhancer	None
TSPAN3 (7)	rs17466257	1.00E-11	0.0001	0.0057	+	Promoter	Promoter
KRT23 (8)	rs12601944	2.10E-13	0.0001	0.0057	+	None	None
CTNS (12)	rs4790532	3.00E-14	0.0001	0.0057	-	None	None
SLC39A13 (1)	rs12803525	2.50E-34	0.0001	0.0057	-	None	None
MAEL (8)	rs7551881	1.20E-07	0.0001	0.0057	-	None	None
AC074117.10 (2)	rs7586601	6.20E-08	0.0001	0.0057	-	None	None
MADD (7)	rs10501320	4.70E-09	0.0001	0.0057	-	Enhancer	Enhancer
IL1R2 (4)	rs3218848	6.80E-09	0.0001	0.0057	-	None	None
AF131215.9 (0)	rs718427	2.60E-08	0.0001	0.0057	+	Repressed	Repressed
RMDN1 (5)	rs10093729	3.80E-07	0.0001	0.0057	-	None	None
RP13-20L14.6 (3)	rs28561791	1.40E-08	0.0001	0.0057	-	None	None
LRPAP1 (2)	rs9683647	1.70E-08	0.0001	0.0057	+	Repressed	Repressed
NDUFAF6 (0)	rs896851	7.20E-11	0.0001	0.0057	-	Transcription	Transcription
NPNT (2)	rs34712979	3.00E-11	0.0001	0.0057	-	Enhancer	Promoter
LMF2 (3)	rs131824	2.00E-09	0.0001	0.0057	-	Enhancer	Enhancer
C11orf24 (3)	rs3802746	4.50E-26	0.0001	0.0057	+	Transcription	None
RP11-1018N14.5 (2)	rs77338032	1.70E-09	0.0001	0.0057	+	None	None
ATG7 (27)	rs11712897	1.00E-07	0.0001	0.0057	-	None	Repressed
CTA-211A9.5 (0)	rs2032576	2.40E-14	0.0001	0.0057	+	Transcription	None
ARL14EP (0)	rs2065940	3.20E-11	0.0001	0.0057	-	None	Transcription
PUS3 (3)	rs513869	1.50E-08	0.0001	0.0057	-	Enhancer	Promoter
RP11-665C16.6 (0)	rs8023197	6.70E-10	0.0001	0.0057	-	Enhancer	Enhancer
PRR9 (1)	rs10788861	1.70E-07	0.0001	0.0057	+	Repressed	Repressed
GSTT1 (1)	rs5760124	9.00E-12	0.0001	0.0057	+	Enhancer	Enhancer
RP11-267N12.3 (5)	rs12731980	1.20E-10	0.0001	0.0057	-	None	None
AC015933.2 (2)	rs1816825	9.20E-09	0.0001	0.0057	-	Enhancer	Enhancer
KIAA0040 (2)	rs760486	8.00E-10	0.0001	0.0057	-	Enhancer	Enhancer
CYP4F11 (0)	rs3810427	3.50E-12	0.0001	0.0057	-	Repressed	None
C4orf47 (4)	rs4862531	2.90E-08	0.0001	0.0057	-	None	None
ANXA5 (1)	rs10857085	9.90E-09	0.0001	0.0057	-	None	Transcription
FAM83F (2)	rs3021217	1.20E-09	0.0001	0.0057	+	Enhancer	Enhancer
TM2D3 (1)	rs12595154	8.10E-11	0.0001	0.0057	-	Repressed	Repressed
CENPQ (1)	rs2516122	1.20E-07	0.0001	0.0057	+	None	None
CTD-2012J19.3 (2)	rs10061756	5.70E-09	0.0001	0.0057	+	None	None
JAG1 (23)	rs34101498	5.60E-11	0.0001	0.0057	+	None	Enhancer
RP11-23J9.4 (27)	rs6478014	8.00E-08	0.0001	0.0057	+	None	None
RP11-326I11.4 (1)	rs1991229	7.50E-08	0.0001	0.0057	-	Promoter	Promoter
C4A (21)	rs204991	2.60E-11	0.0001	0.0057	-	None	Enhancer
NUTM2E (4)	rs184609337	6.10E-10	0.0001	0.0057	+	None	None
ANXA2 (19)	rs12911320	4.10E-08	0.0001	0.0057	+	Enhancer	Enhancer
HADH (2)	rs763432	2.60E-16	0.0001	0.0057	-	Transcription	Enhancer
PTPN18 (16)	rs7570220	4.20E-07	0.0001	0.0057	-	Promoter	Promoter
XRCC6BP1 (0)	rs4590915	2.30E-10	0.0001	0.0057	-	Promoter	Promoter
KLHL18 (0)	rs59299766	4.60E-11	0.0001	0.0057	-	None	None
DUSP18 (2)	rs5753268	4.30E-11	0.0001	0.0057	-	Enhancer	None
RPS26 (0)	rs10876864	5.20E-22	0.0001	0.0057	-	Promoter	Promoter
AC068282.3 (0)	rs6735779	1.90E-27	0.0001	0.0057	+	Enhancer	Enhancer
SPG20 (13)	rs9547250	3.20E-11	0.0001	0.0057	-	None	None
GTF3A (3)	rs1218824	4.90E-08	0.0001	0.0057	-	Transcription	Transcription
ZMPSTE24 (3)	rs6600324	3.20E-12	0.0001	0.0057	+	None	None
NUDT14 (1)	rs3784236	2.60E-10	0.0001	0.0057	-	None	None
RP4-610C12.3 (0)	rs6059244	7.50E-07	0.0001	0.0057	-	Repressed	None
TAB2 (0)	rs576022	1.30E-08	0.0001	0.0057	-	None	None
RBM25 (0)	rs78831876	5.40E-09	0.0001	0.0057	-	None	None
AGBL3 (0)	rs2252826	1.10E-23	0.0001	0.0057	-	Enhancer	Enhancer
PPT1 (0)	rs3131679	4.30E-10	0.0001	0.0057	-	None	None
SLC25A26 (2)	rs146159281	3.00E-08	0.0001	0.0057	+	None	None
BEND6 (1)	rs720885	3.80E-09	0.0001	0.0057	-	None	None
ZNF107 (6)	rs1596929	1.30E-13	0.0001	0.0057	-	None	None
CTD-2245F17.3 (1)	rs4801968	3.20E-18	0.0001	0.0057	-	Transcription	Promoter
RP11-15A1.7 (0)	rs62116613	2.10E-09	0.0001	0.0057	-	Promoter	Promoter
CDC25A (2)	rs13068288	5.90E-08	0.0001	0.0057	+	Transcription	None
ARHGEF19 (0)	rs61769780	8.90E-09	0.0001	0.0057	+	Promoter	Promoter
RP11-1070N10.5 (3)	rs12892323	2.60E-11	0.0001	0.0057	-	None	Promoter
IP6K3 (0)	rs35964955	2.30E-08	0.0001	0.0057	+	None	None
PTGR1 (11)	rs41279069	1.00E-08	0.0001	0.0057	-	Promoter	Promoter
HLA-DQB1-AS1 (0)	rs9273394	3.00E-21	0.0001	0.0057	-	Repressed	None
NPHP4 (11)	rs1287637	3.10E-13	0.0001	0.0057	-	Transcription	None
KCNMB2 (6)	rs11924270	2.00E-10	0.0001	0.0057	-	Enhancer	Enhancer
FHAD1 (35)	rs10927749	8.50E-08	0.0001	0.0057	+	None	None
TMEM236 (3)	rs184811700	6.20E-13	0.0001	0.0057	-	None	None
DHRS4L2 (9)	rs1885809	9.10E-18	0.0001	0.0057	-	None	None
S100A14 (0)	rs3806234	6.90E-09	0.0001	0.0057	-	Promoter	Promoter
C19orf77 (0)	rs67748282	4.30E-08	0.0001	0.0057	+	Enhancer	Enhancer
SMIM2 (0)	rs12869383	2.30E-16	0.0001	0.0057	-	None	None
RPS20 (0)	rs4360268	3.40E-10	0.0001	0.0057	-	None	None
PACSIN3 (10)	rs4647713	6.40E-10	0.0001	0.0057	+	Enhancer	None
RP11-379B18.5 (5)	rs2979990	1.80E-17	0.0001	0.0057	+	None	None
RP11-7F17.7 (0)	rs6574341	3.00E-10	0.0001	0.0057	-	Enhancer	Enhancer
TPGS2 (0)	rs948636	1.70E-26	0.0001	0.0057	+	None	None
RPL31 (3)	rs78362750	6.90E-24	0.0001	0.0057	-	Enhancer	Enhancer
AP3S2 (6)	rs4932261	5.10E-09	0.0001	0.0057	-	Transcription	None
RP11-661A12.5 (0)	rs9314409	2.20E-14	0.0001	0.0057	-	Enhancer	Enhancer

WARS2 (0)	rs61808892	3.40E-14	0.0001	0.0057	-	None	None
PPP2R2A (11)	rs7007256	1.20E-09	0.0001	0.0057	-	None	None
RP11-7O14.1 (2)	rs1564001	5.90E-09	0.0001	0.0057	-	None	None
C9orf78 (0)	rs3758335	5.50E-13	0.0001	0.0057	-	Promoter	Promoter
GRAMD1A (15)	rs8103441	1.70E-11	0.0001	0.0057	-	Enhancer	Enhancer
YWHAQ (6)	rs10203320	1.30E-10	0.0001	0.0057	-	Enhancer	Promoter
AC005775.2 (0)	rs1574520	4.20E-09	0.0001	0.0057	-	Promoter	Promoter
INTS12 (5)	rs72671853	1.10E-11	0.0001	0.0057	-	Transcription	None
ZNF429 (6)	rs12463354	1.10E-07	0.0001	0.0057	-	None	None
C4B (19)	rs140381857	1.80E-19	0.0001	0.0057	-	None	None
RPS27L (1)	rs28678549	4.20E-12	0.0001	0.0057	-	Transcription	None
HSD17B11 (0)	rs7685308	1.50E-07	0.0001	0.0057	+	Transcription	Transcription
GBP3 (0)	rs2390677	1.30E-17	0.0001	0.0057	-	None	None
HLA-DQA2 (2)	rs9272365	5.00E-19	0.0001	0.0057	+	None	None
TTLL12 (0)	rs9612021	2.40E-09	0.0001	0.0057	-	Promoter	Promoter
VPS28 (0)	rs117275955	3.40E-11	0.0001	0.0057	+	None	None
SLC2A14 (4)	rs140493080	1.10E-10	0.0001	0.0057	+	None	None
ZNF880 (4)	rs10445586	1.30E-26	0.0001	0.0057	-	Transcription	Promoter
RP11-650P15.1 (1)	rs9962333	1.30E-09	0.0001	0.0057	+	None	None
SNHG7 (2)	rs115005960	2.40E-18	0.0001	0.0057	-	None	None
SLC36A1 (1)	rs12655145	1.30E-16	0.0001	0.0057	-	None	None
TAF1B (11)	rs2245344	8.90E-18	0.0001	0.0057	+	None	Enhancer
LINC00698 (11)	rs11130905	1.20E-08	0.0001	0.0057	-	Enhancer	Enhancer
CDK5RAP2 (0)	rs3780674	3.80E-09	0.0001	0.0057	-	None	Transcription
CTRB2 (2)	rs72802342	5.00E-09	0.0001	0.0057	+	None	Enhancer
EIF5 (1)	rs7143221	9.30E-20	0.0001	0.0057	-	None	None
RP11-770G2.4 (0)	rs10896045	1.70E-08	0.0001	0.0057	+	Promoter	Promoter
ZNF544 (10)	rs260495	1.00E-12	0.0001	0.0057	-	None	Transcription
SLC39A14 (0)	rs56670567	7.80E-08	0.0001	0.0057	-	Transcription	Transcription
R3HCC1 (6)	rs73224428	1.00E-13	0.0001	0.0057	-	Transcription	Enhancer
SRR (3)	rs2131700	1.60E-08	0.0001	0.0057	-	None	None
BIN1 (0)	rs78710909	1.10E-09	0.0001	0.0057	-	Promoter	Promoter
CBWD2 (15)	rs2592462	5.80E-19	0.0001	0.0057	-	None	None
AF131216.5 (0)	rs4841525	6.80E-08	0.0001	0.0057	+	None	None
SLC2A8 (10)	rs10987643	2.40E-08	0.0001	0.0057	-	None	None
COBL (4)	rs62448278	3.20E-23	0.0001	0.0057	-	Enhancer	Enhancer
CNGA1 (0)	rs321638	9.00E-12	0.0001	0.0057	+	None	None
AF131215.2 (0)	rs7460507	2.70E-08	0.0001	0.0057	-	None	None
SLC1A6 (0)	rs112816712	2.10E-12	0.0001	0.0057	+	Repressed	None
RP11-316O14.1 (0)	rs12474050	4.40E-09	0.0001	0.0057	+	Enhancer	Enhancer
ZSWIM7 (2)	rs4435297	1.20E-10	0.0001	0.0057	-	Transcription	Transcription
AC003075.4 (6)	rs4719497	6.90E-15	0.0001	0.0057	+	None	None
PSG4 (10)	rs2354278	7.20E-08	0.0001	0.0057	-	None	None
CCZ1 (6)	chr7:5949726	2.20E-12	0.0001	0.0057	-	None	None
PSTPIP1 (22)	rs11630497	1.40E-07	0.0001	0.0057	-	None	Transcription
MANBA (16)	rs223489	2.80E-14	0.0001	0.0057	-	Enhancer	Enhancer
WAC (3)	rs12763015	7.10E-14	0.0001	0.0057	+	Enhancer	Enhancer
MGST1 (12)	rs1024839	3.20E-08	0.0001	0.0057	-	None	None
TRAPPC13 (3)	rs10805414	2.30E-08	0.0001	0.0057	+	None	None
RP11-592B15.3 (0)	rs61847076	1.30E-07	0.0001	0.0057	+	None	Transcription
CPNE4 (23)	rs4854894	1.10E-10	0.0001	0.0057	+	Enhancer	Enhancer
DOPEY1 (6)	rs140499402	7.50E-08	0.0001	0.0057	-	None	None
OSBPL1A (28)	rs7226913	2.30E-08	0.0001	0.0057	-	None	None
ELP5 (0)	rs2654183	2.40E-19	0.0001	0.0057	-	Transcription	Transcription
ST7L (0)	rs351372	4.60E-09	0.0001	0.0057	-	Enhancer	None
SNX16 (0)	rs774474	4.60E-14	0.0001	0.0057	-	None	None
DCST2 (6)	rs822507	4.00E-07	0.0001	0.0057	+	Transcription	None
SOGA2 (18)	rs632423	1.80E-15	0.0001	0.0057	-	Transcription	Transcription
CTC-338M12.3 (0)	rs2112263	2.10E-09	0.0001	0.0057	-	Insulator	None
PRKRIP1 (4)	rs1734769	1.90E-08	0.0001	0.0057	+	None	None
MEA1 (2)	rs76552409	4.30E-08	0.0001	0.0057	+	Transcription	None
ARL6IP6 (3)	rs10185455	1.10E-07	0.0001	0.0057	+	Enhancer	Transcription
CCDC66 (19)	rs56204053	4.50E-16	0.0001	0.0057	+	None	None
SOAT2 (2)	rs2085503	5.70E-08	0.0001	0.0057	+	None	None
MAP4 (12)	rs36033865	4.00E-07	0.0001	0.0057	-	None	None
NARF (3)	rs36168299	8.40E-09	0.0001	0.0057	-	Promoter	Promoter
RAP1GDS1 (2)	rs2034677	4.40E-14	0.0001	0.0057	+	None	None
AC004870.4 (4)	rs4724521	7.60E-10	0.0001	0.0057	+	None	None
ANKRD30B (31)	rs9947578	9.60E-08	0.0001	0.0057	+	None	None
STK32C (17)	rs2474336	3.90E-18	0.0001	0.0057	-	Transcription	None
ST3GAL6-AS1 (0)	rs13065271	8.30E-08	0.0001	0.0057	-	None	None
FAM189B (0)	rs1076556	3.80E-07	0.0001	0.0057	-	Transcription	Transcription
CAT (1)	rs7120960	3.40E-09	0.0001	0.0057	-	Transcription	None
ROBO3 (26)	rs4635093	4.20E-08	0.0001	0.0057	-	Repressed	Repressed
OLA1 (0)	rs10930632	5.40E-14	0.0001	0.0057	+	Transcription	None
FBXO18 (4)	rs188230198	1.50E-08	0.0001	0.0057	-	None	None
RORA (22)	rs4775370	3.30E-12	0.0001	0.0057	-	None	None
HMGN1 (0)	rs914156	2.20E-11	0.0001	0.0057	-	Enhancer	Enhancer
TRIO (1)	rs76204195	5.30E-08	0.0001	0.0057	-	None	Enhancer
FAM153B (28)	rs13169355	5.60E-11	0.0001	0.0057	+	None	None
HLA-DRB5 (0)	rs9271147	4.10E-53	0.0001	0.0057	-	None	Enhancer
AIFM2 (9)	rs884195	4.60E-08	0.0001	0.0057	-	None	Transcription
RPS16 (2)	rs480656	9.70E-08	0.0001	0.0057	-	Enhancer	Enhancer
YWHAB (0)	rs6031849	2.40E-25	0.0001	0.0057	-	Promoter	Promoter
F11R (0)	rs4656975	3.50E-21	0.0001	0.0057	-	Enhancer	Enhancer
ARSB (3)	rs34902218	7.50E-09	0.0001	0.0057	-	None	None
RP5-828H9.1 (2)	rs2423103	1.70E-14	0.0001	0.0057	+	None	None
CSE1L (0)	rs11697370	2.80E-08	0.0001	0.0057	+	Transcription	Transcription
RP11-162G9.1 (5)	rs2378804	3.50E-10	0.0001	0.0057	-	Enhancer	Enhancer
FN3KRP (4)	rs2246577	1.00E-07	0.0001	0.0057	-	Transcription	None
RNF13 (1)	rs13070249	4.00E-10	0.0001	0.0057	+	None	None
FAM115C (5)	rs7811904	4.30E-14	0.0001	0.0057	-	Enhancer	Enhancer
APBB1IP (1)	rs4749125	3.40E-09	0.0001	0.0057	-	Promoter	Promoter
TNNI1 (0)	rs2799680	1.20E-14	0.0001	0.0057	-	Enhancer	Enhancer

KIAA1429 (19)	rs56042558	2.20E-08	0.0001	0.0057	-	None	None
RP4-717I23.3 (10)	rs11164923	1.70E-08	0.0001	0.0057	+	None	None
CHMP4C (0)	rs111683632	1.50E-14	0.0001	0.0057	-	None	None
SNAPC5 (0)	rs77428206	8.90E-09	0.0001	0.0057	-	None	None
SMARCB1 (1)	rs17003930	5.10E-08	0.0001	0.0057	-	Transcription	Transcription
CRYZ (1)	rs28583318	9.70E-10	0.0001	0.0057	-	None	None
RGS3 (36)	rs10817493	1.20E-12	0.0001	0.0057	-	Enhancer	Promoter
CELA3B (1)	rs116802276	2.20E-11	0.0001	0.0057	+	None	Enhancer
SPAG1 (19)	rs2453655	2.80E-08	0.0001	0.0057	-	None	None
ICA1L (14)	rs7568438	3.20E-09	0.0001	0.0057	+	None	None
CDC42EP1 (3)	rs2281097	1.50E-14	0.0001	0.0057	+	Enhancer	Enhancer
RP11-44F14.2 (2)	rs2908795	1.10E-07	0.0001	0.0057	-	None	None
SCAPER (0)	rs932047	3.50E-07	0.0001	0.0057	+	None	None
LRRC69 (11)	rs4551303	3.70E-08	0.0001	0.0057	-	None	None
ZNF749 (0)	rs1858991	5.10E-20	0.0001	0.0057	+	Transcription	Promoter
KRT1 (0)	rs1994516	2.70E-08	0.0001	0.0057	+	None	Repressed
RP11-713P17.5 (1)	rs11606293	5.10E-10	0.0001	0.0057	+	Repressed	Repressed
UQCRH (1)	rs77526109	7.20E-08	0.0001	0.0057	-	Transcription	Transcription
MYO15B (22)	rs117594295	1.40E-07	0.0001	0.0057	+	Promoter	Promoter
IMPA1 (7)	rs10096714	5.60E-08	0.0001	0.0057	-	Transcription	Transcription
UBE2F (4)	rs11678880	6.10E-15	0.0001	0.0057	+	None	Transcription
PDPR (3)	rs4985462	1.10E-07	0.0001	0.0057	-	None	None
RP11-799B12.2 (0)	rs113722141	1.20E-08	0.0001	0.0057	+	None	Enhancer
ZNF814 (0)	rs28374851	1.20E-08	0.0001	0.0057	-	Promoter	Promoter
ADO (0)	rs10995307	1.20E-10	0.0001	0.0057	-	None	None
ZNF468 (4)	rs58476100	3.00E-09	0.0001	0.0057	-	Transcription	Transcription
ALOX5 (0)	rs150423942	6.40E-11	0.0001	0.0057	-	None	None
TMEM106B (1)	rs12666279	1.00E-08	0.0001	0.0057	-	Enhancer	Enhancer
ZNF132 (0)	rs4801587	7.70E-16	0.0001	0.0057	-	None	None
ELL2 (5)	rs3777183	1.20E-08	0.0001	0.0057	-	Enhancer	Enhancer
FOXD4L1 (0)	rs2360232	4.00E-13	0.0001	0.0057	-	None	Promoter
RIOK2 (7)	rs31000	5.70E-07	0.0001	0.0057	-	None	Transcription
SMLR1 (0)	rs2008598	5.50E-13	0.0001	0.0057	-	None	None
NDUFA3 (3)	rs7253859	2.60E-08	0.0001	0.0057	+	None	None
C12orf73 (1)	rs322102	4.80E-08	0.0001	0.0057	+	Promoter	Promoter
RP11-486L19.2 (0)	rs2303243	2.80E-08	0.0001	0.0057	+	Repressed	Repressed
TAS2R43 (0)	rs2597994	2.00E-13	0.0001	0.0057	-	Transcription	None
TMEM51 (2)	rs3753318	1.20E-14	0.0001	0.0057	-	None	Enhancer
CHRNA7 (7)	rs11857238	6.00E-09	0.0001	0.0057	+	Promoter	Promoter
SBF2-AS1 (4)	rs7935673	8.80E-10	0.0001	0.0057	-	Transcription	Transcription
PANX1 (3)	rs1138800	2.20E-07	0.0001	0.0057	+	Promoter	Promoter
RASSF1 (6)	rs6776145	2.20E-09	0.0001	0.0057	+	None	None
CBLN3 (0)	rs2255591	7.60E-10	0.0001	0.0057	+	Enhancer	Enhancer
ASPSCR1 (20)	rs117148068	3.90E-08	0.0001	0.0057	-	Transcription	Transcription
ZNF577 (6)	rs60088244	6.20E-09	0.0001	0.0057	-	Transcription	Promoter
WBSCR27 (0)	rs6460060	9.60E-17	0.0001	0.0057	-	None	None
SPATC1L (0)	rs62215180	3.50E-09	0.0001	0.0057	-	None	None
PPIL3 (0)	rs2136600	2.00E-18	0.0001	0.0057	-	Transcription	Transcription
PCGF6 (9)	rs60388741	1.40E-07	0.0001	0.0057	-	Transcription	None
NIPAL4 (1)	rs10063083	2.70E-09	0.0001	0.0057	-	None	None
HSD17B4 (21)	rs39971	1.00E-13	0.0001	0.0057	+	Transcription	Transcription
FAM228B (10)	rs2042468	5.80E-10	0.0001	0.0057	+	None	Enhancer
ANAPC1 (12)	rs2948019	7.40E-09	0.0001	0.0057	-	None	None
PKHD1 (29)	rs10948693	1.20E-08	0.0001	0.0057	-	None	None
POTEE (14)	rs113400623	2.30E-10	0.0001	0.0057	+	None	None
SMC4 (6)	rs1873076	9.10E-08	0.0001	0.0057	-	Transcription	Transcription
DCBLD2 (0)	rs140020059	2.50E-08	0.0001	0.0057	-	None	None
GCC2 (10)	rs13386343	7.60E-08	0.0001	0.0057	+	None	None
C21orf62 (1)	rs12482132	1.90E-11	0.0001	0.0057	-	None	None
FOPNL (1)	rs74009465	4.70E-12	0.0001	0.0057	-	Repressed	None
EFCAB5 (18)	rs62070565	4.70E-07	0.0001	0.0057	+	Transcription	Transcription
SKIV2L (13)	rs112675983	8.80E-06	0.0001	0.0057	+	None	None
NPIPA1 (10)	rs118059365	1.70E-09	0.0001	0.0057	-	Enhancer	Transcription
EIF3C (10)	rs80275162	6.20E-08	0.0001	0.0057	-	None	None
MMP9 (0)	rs8114050	9.90E-08	0.0001	0.0057	+	Repressed	Repressed
PCDHA10 (0)	rs11741879	1.30E-17	0.0001	0.0057	-	Transcription	Promoter
OXTR (0)	rs62243375	3.10E-11	0.0001	0.0057	-	Promoter	Enhancer
BTN3A2 (0)	rs71276077	1.60E-09	0.0001	0.0057	-	None	None
GPHN (23)	rs72718423	4.10E-08	0.0001	0.0057	-	Enhancer	None
IMMT (3)	rs12475079	5.10E-08	0.0001	0.0057	-	Enhancer	Enhancer
COL18A1 (10)	rs1131100	4.70E-11	0.0001	0.0057	-	Repressed	Repressed
UGT2B7 (0)	rs7660019	1.10E-13	0.0001	0.0057	-	None	None
LEMD2 (0)	rs12190029	1.40E-09	0.0001	0.0057	-	Enhancer	Transcription
NAT16 (0)	rs6465790	6.50E-12	0.0001	0.0057	+	None	None
RP11-385F5.4 (0)	rs10925177	7.70E-09	0.0001	0.0057	-	None	Repressed
PARP4 (8)	rs7322323	8.50E-08	0.0001	0.0057	-	Transcription	Transcription
AC006465.3 (1)	rs886726	1.10E-08	0.0001	0.0057	-	None	Transcription
GOLGA8N (7)	rs114770598	2.10E-24	0.0001	0.0057	-	None	None
USP30-AS1 (0)	rs10774961	6.50E-10	0.0001	0.0057	-	Transcription	Transcription
RP11-379F4.4 (5)	rs73017539	9.00E-09	0.0001	0.0057	-	Transcription	Transcription
BMP8A (3)	rs1053846	4.90E-09	0.0001	0.0057	+	Enhancer	Transcription
GOT2 (8)	rs1595181	2.60E-10	0.0001	0.0057	-	None	None
AC007319.1 (8)	rs144956220	1.40E-13	0.0001	0.0057	+	None	None
ART3 (6)	rs71629035	7.00E-12	0.0001	0.0057	-	None	None
LRRIQ3 (10)	rs11210442	5.30E-08	0.0001	0.0057	-	None	None
MAPK12 (8)	rs2341111	1.30E-10	0.0001	0.0057	-	Enhancer	Enhancer
OR3A2 (0)	rs151174886	8.90E-14	0.0001	0.0057	+	None	None
AGA (0)	rs11131799	2.60E-09	0.0001	0.0057	+	Promoter	Promoter
SKIL (0)	rs67298747	4.90E-09	0.0001	0.0057	-	None	None
PRPH2 (0)	rs3818087	3.60E-16	0.0001	0.0057	+	Enhancer	Promoter
RP11-112L6.4 (0)	rs4283476	1.60E-10	0.0001	0.0057	+	None	Enhancer
APLP2 (8)	rs78299844	2.10E-09	0.0001	0.0057	-	None	None
COX7A2L (1)	rs10178208	9.00E-12	0.0001	0.0057	-	Enhancer	Enhancer
TAPBPL (3)	rs2041387	4.70E-09	0.0001	0.0057	+	Enhancer	Enhancer

NDUFAF4 (1)	rs9400365	3.70E-13	0.0001	0.0057	-	None	None
FBLL1 (0)	rs71589841	4.90E-18	0.0001	0.0057	+	None	None
RP11-182J1.16 (1)	rs12595694	1.40E-07	0.0001	0.0057	+	None	None
C18orf42 (1)	rs11662559	2.70E-09	0.0001	0.0057	-	Enhancer	None
ZNF257 (8)	rs56467296	1.10E-08	0.0001	0.0057	+	None	Promoter
UBAC1 (4)	rs11103237	1.50E-09	0.0001	0.0057	-	Enhancer	Enhancer
RAPGEF3 (0)	rs73302702	1.30E-11	0.0001	0.0057	+	None	None
ZBTB20 (30)	rs11706205	3.90E-10	0.0001	0.0057	+	None	None
VARS2 (2)	rs114969940	1.00E-09	0.0001	0.0057	+	Promoter	Enhancer
RP11-597D13.9 (6)	rs2346775	9.20E-09	0.0001	0.0057	+	None	None
ZNF658 (3)	rs28763593	2.00E-17	0.0001	0.0057	-	None	None
CTR9 (11)	rs3736326	8.20E-09	0.0001	0.0057	+	Transcription	Transcription
GAA (3)	rs2304838	2.30E-15	0.0001	0.0057	-	None	None
HCG22 (2)	rs2523857	3.90E-10	0.0001	0.0057	-	None	Repressed
SYNDIG1L (0)	rs8009761	1.10E-15	0.0001	0.0057	-	Enhancer	Enhancer
CTBP2 (11)	rs1152658	8.80E-09	0.0001	0.0057	+	None	Enhancer
MTRR (22)	rs327588	3.20E-08	0.0001	0.0057	-	Transcription	Promoter
CD276 (12)	rs55868793	1.10E-07	0.0001	0.0057	+	None	None
RMI2 (4)	rs10163410	8.50E-10	0.0001	0.0057	+	None	None
WDR41 (0)	rs17683584	1.30E-11	0.0001	0.0057	-	None	None
LINC00339 (1)	rs11586488	2.80E-13	0.0001	0.0057	+	None	None
ALDH3A2 (14)	rs962800	2.40E-36	0.0001	0.0057	+	Transcription	Transcription
CCDC67 (10)	rs7943372	3.00E-12	0.0001	0.0057	+	Enhancer	Enhancer
COA6 (0)	rs6669190	1.50E-09	0.0001	0.0057	+	None	Enhancer
GSTM4 (3)	rs1010167	5.00E-08	0.0001	0.0057	-	Promoter	Promoter
BIN3 (3)	rs7817772	3.00E-11	0.0001	0.0057	-	None	None
HIBCH (3)	rs2582770	5.40E-07	0.0001	0.0057	+	None	None
RP11-421F16.3 (0)	rs146970669	4.00E-12	0.0001	0.0057	-	None	None
SARDH (13)	rs476835	2.70E-08	0.0001	0.0057	-	Enhancer	Promoter
FRK (1)	rs12201275	1.40E-08	0.0001	0.0057	-	None	None
TBC1D3P1-DHX40P1 (4)	rs56155082	2.10E-23	0.0001	0.0057	+	Enhancer	Enhancer
ASAH1 (12)	rs3753115	6.10E-10	0.0001	0.0057	-	Enhancer	Enhancer
GLB1 (8)	rs6780220	4.10E-09	0.0001	0.0057	+	Transcription	None
CD44 (19)	rs10128586	9.80E-08	0.0001	0.0057	-	Enhancer	Transcription
RPL3L (1)	rs758335	7.80E-12	0.0001	0.0057	-	Transcription	Enhancer
TMED6 (0)	rs153060	1.90E-20	0.0001	0.0057	-	Transcription	Enhancer
PODXL (7)	rs1733877	1.20E-07	0.0001	0.0057	-	Repressed	None
RP11-66B24.2 (3)	rs7178773	4.60E-09	0.0001	0.0057	-	Promoter	Promoter
C6orf15 (0)	rs3094197	2.70E-10	0.0001	0.0057	-	None	Enhancer
SUMF1 (10)	rs11915920	4.20E-12	0.0001	0.0057	-	Enhancer	None
RP4-735C1.4 (2)	rs4970777	1.10E-16	0.0001	0.0057	+	Insulator	None
QTRTD1 (9)	rs1386478	2.10E-09	0.0001	0.0057	-	Enhancer	Transcription
ENGASE (3)	rs12452651	3.50E-08	0.0001	0.0057	-	Transcription	None
DNAJC12 (3)	rs112586929	4.10E-14	0.0001	0.0057	-	None	None
TAMM41 (11)	rs303857	2.10E-32	0.0001	0.0057	+	Transcription	Transcription
ATP12A (1)	rs61948108	5.20E-12	0.0001	0.0057	+	None	None
RP5-1021I20.4 (0)	rs8008427	1.20E-09	0.0001	0.0057	+	None	None
HLA-DPA1 (0)	rs114841687	2.30E-19	0.0001	0.0057	-	None	None
CTC-360G5.9 (0)	rs12609975	3.40E-09	0.0001	0.0057	-	None	None
IRAK1BP1 (4)	rs6454092	2.80E-16	0.0001	0.0057	+	Transcription	Transcription
ERV3-1 (2)	rs188662478	4.20E-10	0.0001	0.0057	+	None	None
IQGAP2 (17)	rs2455221	5.20E-14	0.0001	0.0057	-	None	None
RP11-316M1.12 (0)	rs6693120	4.00E-25	0.0001	0.0057	-	None	None
CRAT (16)	rs7861663	1.00E-11	0.0001	0.0057	+	None	None
UCA1 (1)	rs59539750	9.70E-09	0.0001	0.0057	+	None	None
CTSH (8)	rs2289702	2.70E-07	0.0001	0.0057	-	Enhancer	Promoter
RNH1 (10)	rs11821448	2.00E-22	0.0001	0.0057	-	None	None
CCDC77 (12)	rs2369277	8.10E-09	0.0001	0.0057	+	None	None
F11R (0)	rs4656975	3.00E-09	0.0001	0.0057	-	Enhancer	Enhancer
KCTD21-AS1 (1)	rs12273235	2.50E-09	0.0001	0.0057	-	None	None
CCDC9 (13)	rs73061392	3.60E-11	0.0001	0.0057	-	None	None
RP11-568A7.3 (0)	rs2092092	5.50E-10	0.0001	0.0057	+	Repressed	None
RAB5A (0)	rs12714980	3.60E-19	0.0001	0.0057	-	None	None
USP40 (0)	rs201182024	7.30E-09	0.0001	0.0057	-	None	Transcription
RP11-426C22.6 (0)	rs3891915	4.80E-13	0.0001	0.0057	-	None	Enhancer
RP11-423H2.3 (4)	rs62399486	1.20E-09	0.0001	0.0057	+	None	None
PON1 (1)	rs705381	6.10E-12	0.0001	0.0057	-	Enhancer	Promoter
LGALS8 (4)	rs2564734	3.40E-09	0.0001	0.0057	-	Transcription	Transcription
TMC4 (14)	rs4806498	2.60E-09	0.0001	0.0057	-	None	None
GOLGA6L9 (8)	rs146795433	3.40E-13	0.0001	0.0057	+	None	None
DEPDC5 (22)	rs5998175	2.20E-08	0.0001	0.0057	-	None	None
DHRS4 (0)	rs7156226	1.30E-08	0.0001	0.0057	-	None	None
RNPEP (11)	rs10920302	4.50E-09	0.0001	0.0057	-	Transcription	Enhancer
SMIM2-AS1 (1)	rs9525917	1.90E-19	0.0001	0.0057	-	None	None
ARIH2 (3)	rs7372149	7.10E-12	0.0001	0.0057	+	None	None
ATP2C2 (0)	rs41367549	1.30E-09	0.0001	0.0057	+	None	None
ATHL1 (11)	rs760060	1.90E-08	0.0001	0.0057	+	Promoter	Promoter
KRTAP5-AS1 (4)	rs60210378	3.60E-13	0.0001	0.0057	-	Repressed	Repressed
RP11-467C18.1 (0)	rs835367	5.00E-08	0.0001	0.0057	-	Promoter	Promoter
RP11-745O10.2 (1)	rs7966213	3.50E-18	0.0001	0.0057	-	None	None
TMEM180 (2)	rs3961455	3.60E-08	0.0001	0.0057	-	Enhancer	Enhancer
PRPF18 (1)	rs10906419	3.70E-12	0.0001	0.0057	-	None	None
C7 (18)	rs1450665	3.50E-11	0.0001	0.0057	-	None	None
RNF144B (1)	rs1886248	8.50E-12	0.0001	0.0057	-	None	None
NARS (2)	rs34027711	3.20E-08	0.0001	0.0057	+	Enhancer	Enhancer
AC021218.2 (1)	rs11764203	1.60E-09	0.0001	0.0057	+	None	None
GTF2H1 (7)	rs11024586	1.00E-07	0.0001	0.0057	+	None	None
TBKBP1 (10)	rs6503796	6.60E-10	0.0001	0.0057	-	Enhancer	Enhancer
UGDH (13)	rs62308020	1.60E-25	0.0001	0.0057	-	Transcription	Transcription
CAP1 (2)	rs12406090	6.60E-11	0.0001	0.0057	-	None	None
TMEM18 (4)	rs12990777	1.40E-12	0.0001	0.0057	+	None	None
FAM134B (6)	rs332811	1.10E-08	0.0001	0.0057	+	Enhancer	Promoter
TMEM63C (1)	rs1861420	1.60E-15	0.0001	0.0057	-	Enhancer	Enhancer
PLGRKT (3)	rs2104175	9.60E-11	0.0001	0.0057	+	None	None

LRRC16B (3)	rs12888965	2.90E-08	0.0001	0.0057	+	None	Enhancer
NDUFAF1 (1)	rs28463309	1.40E-08	0.0001	0.0057	+	None	None
PCMT1 (4)	rs9479808	2.60E-09	0.0001	0.0057	+	Enhancer	Promoter
LYPD6B (2)	rs4399686	1.20E-16	0.0001	0.0057	+	Repressed	None
ACYP2 (3)	rs805324	1.70E-13	0.0001	0.0057	-	Transcription	Transcription
RP11-798G7.7 (0)	rs117794064	1.30E-11	0.0001	0.0057	-	None	None
DOCK8 (56)	rs593179	3.10E-09	0.0001	0.0057	-	None	None
SIRPB1 (9)	rs6136375	2.80E-14	0.0001	0.0057	+	None	None
REXO1 (13)	rs75443592	2.80E-21	0.0001	0.0057	-	Enhancer	Enhancer
MRPL18 (1)	rs9347340	4.80E-28	0.0001	0.0057	-	Transcription	Transcription
STARD3NL (3)	rs56255857	3.40E-10	0.0001	0.0057	-	Enhancer	Enhancer
AC116609.2 (1)	rs6725467	2.00E-11	0.0001	0.0057	+	None	None
ADPRM (2)	rs4792016	1.40E-10	0.0001	0.0057	+	None	None
RP1-184J9.2 (1)	rs10917212	1.10E-07	0.0001	0.0057	-	Enhancer	Enhancer
WNT3 (6)	rs9904865	2.10E-10	0.0001	0.0057	-	None	None
MRPL23 (8)	rs217240	6.30E-09	0.0001	0.0057	+	None	None
RABEP1 (18)	rs2107151	2.40E-18	0.0001	0.0057	-	Transcription	Transcription
XXbac-BPG254F23.6 (0)	rs114856834	1.30E-14	0.0001	0.0057	-	None	None
GGCX (3)	rs6705839	2.40E-09	0.0001	0.0057	+	None	None
LINC00471 (2)	rs7582474	2.80E-09	0.0001	0.0057	-	None	None
CARKD (8)	rs330558	8.80E-12	0.0001	0.0057	-	Enhancer	Enhancer
NPHP3 (25)	rs66564593	5.90E-12	0.0001	0.0057	-	None	None
PLEKHM1 (6)	rs149133346	1.00E-09	0.0001	0.0057	-	None	Enhancer
AHSA2 (6)	rs7588959	3.80E-08	0.0001	0.0057	-	None	Enhancer
MERTK (18)	rs10187656	5.50E-09	0.0001	0.0057	+	None	None
ENOSF1 (11)	rs2741168	1.30E-10	0.0001	0.0057	+	Transcription	Enhancer
LLGL2 (1)	rs8079362	8.80E-14	0.0001	0.0057	-	Enhancer	None
AFF4 (16)	rs811683	1.10E-19	0.0001	0.0057	-	Transcription	Transcription
THAP3 (5)	rs6678681	4.00E-08	0.0001	0.0057	-	Enhancer	Promoter
ABO (0)	rs494242	6.60E-14	0.0001	0.0057	+	None	None
TGM3 (10)	rs2422681	5.80E-10	0.0001	0.0057	+	None	None
USMG5 (2)	rs1163073	7.00E-33	0.0001	0.0057	+	None	None
ZNF134 (1)	rs2074206	1.90E-09	0.0001	0.0057	-	Transcription	Transcription
TAS2R14 (0)	rs7976610	1.20E-08	0.0001	0.0057	+	None	None
PHIP (1)	rs9361497	4.30E-08	0.0001	0.0057	-	None	None
TTC12 (1)	rs7118188	3.60E-10	0.0001	0.0057	-	None	None
SEC24D (25)	rs13112913	2.10E-09	0.0001	0.0057	-	Transcription	Transcription
ITGA2B (9)	rs12601410	1.70E-08	0.0001	0.0057	+	Enhancer	Enhancer
KANSL1 (13)	rs117275691	1.10E-31	0.0001	0.0057	+	Promoter	Enhancer
RNF212 (10)	rs2290409	1.80E-12	0.0001	0.0057	-	None	Repressed
PLA2G16 (4)	rs7929406	4.00E-07	0.0001	0.0057	+	None	None
AGAP8 (0)	rs7909927	2.10E-11	0.0001	0.0057	+	Promoter	Promoter
RRP7A (3)	rs3985936	6.30E-11	0.0001	0.0057	-	Enhancer	None
FOXN3 (9)	rs58162188	1.50E-09	0.0001	0.0057	-	None	None
XRCC1 (6)	rs3213282	5.00E-08	0.0001	0.0057	-	Enhancer	Enhancer
FRA10AC1 (0)	rs7909452	8.30E-09	0.0001	0.0057	-	None	None
SPEG (52)	rs2010592	2.70E-10	0.0001	0.0057	+	None	Enhancer
MARCH8 (9)	rs112884766	3.40E-12	0.0001	0.0057	+	None	None
SLC3A2 (1)	rs7941706	6.00E-12	0.0001	0.0057	-	Promoter	Promoter
MPZL3 (5)	rs7949751	1.90E-09	0.0001	0.0057	+	None	None
RP5-1185K9.1 (1)	rs6082544	2.20E-08	0.0001	0.0057	-	None	None
SLC38A11 (13)	rs35862463	2.90E-07	0.0001	0.0057	+	None	None
PIKFYVE (37)	rs10177810	4.50E-08	0.0001	0.0057	+	Transcription	Promoter
RP4-584D14.7 (0)	rs11764936	7.20E-09	0.0001	0.0057	+	Transcription	Transcription
PPP2R3C (0)	rs2415262	2.30E-09	0.0001	0.0057	+	None	None
ZNF583 (0)	rs11883027	7.70E-08	0.0001	0.0057	+	None	None
PRR4 (5)	rs2416549	2.00E-12	0.0001	0.0057	-	Enhancer	Promoter
ACTR3C (2)	rs10263777	5.00E-09	0.0001	0.0057	-	None	None
GSTM1 (0)	MERGED_DEL_2_4670_-	3.40E-19	0.0001	0.0057	-	None	None
SMG1 (37)	rs62047579	3.10E-08	0.0001	0.0057	-	None	None
GRB14 (8)	rs2389947	2.60E-08	0.0001	0.0057	-	None	None
MAST4 (4)	rs187569	4.40E-07	0.0001	0.0057	+	None	None
CSGALNACT1 (5)	rs56298301	7.30E-15	0.0001	0.0057	+	None	None
VRK3 (0)	rs112243188	7.50E-16	0.0001	0.0057	-	None	None
RP11-184E9.2 (0)	rs335596	1.00E-17	0.0001	0.0057	-	None	None
FAM21A (10)	rs10824973	6.30E-19	0.0001	0.0057	-	None	None
C11orf73 (3)	rs11234588	1.50E-14	0.0001	0.0057	-	Promoter	Promoter
NOM1 (3)	rs1833140	2.60E-12	0.0001	0.0057	+	Transcription	Transcription
MRPL39 (8)	rs1783013	4.20E-09	0.0001	0.0057	-	None	None
SLC22A31 (0)	rs12929648	1.90E-14	0.0001	0.0057	+	None	None
CEP44 (12)	rs7659617	2.00E-08	0.0001	0.0057	-	Enhancer	None
ATP5G2 (6)	rs3892761	3.80E-13	0.0001	0.0057	+	Enhancer	Enhancer
NUCB2 (26)	rs10832758	3.80E-27	0.0001	0.0057	-	Transcription	Transcription
TMEM255B (12)	rs7400029	1.50E-07	0.0001	0.0057	-	Repressed	Repressed
MLF1 (3)	rs17629414	7.30E-17	0.0001	0.0057	-	Enhancer	Enhancer
COA1 (8)	rs17724202	9.70E-32	0.0001	0.0057	+	None	None
ERVK13-1 (0)	rs36087603	7.10E-14	0.0001	0.0057	-	Enhancer	Promoter
CARS2 (16)	rs3818496	2.20E-08	0.0001	0.0057	+	Promoter	Promoter
MRPL20 (2)	rs6603787	4.80E-10	0.0001	0.0057	+	None	None
DHRS7 (0)	rs1254028	4.30E-11	0.0001	0.0057	-	Promoter	Promoter
CTNNA1 (33)	rs10076442	3.40E-10	0.0001	0.0057	-	None	None
ST6GAL1 (22)	rs12632862	7.80E-09	0.0001	0.0057	+	None	None
XXbac-BPG181B23.7 (0)	rs116704939	8.10E-19	0.0001	0.0057	-	None	Repressed
TACC1 (19)	rs6998357	5.50E-08	0.0001	0.0057	+	None	Transcription
NUDCD2 (2)	rs177250	1.10E-08	0.0001	0.0057	-	Transcription	Transcription
IGSF21 (6)	rs2027530	2.80E-08	0.0001	0.0057	+	Enhancer	Enhancer
TIPIN (0)	rs34842367	1.00E-07	0.0001	0.0057	+	None	None
NOP10 (0)	rs56057955	1.60E-12	0.0001	0.0057	-	None	Enhancer
BET1L (6)	rs1023430	3.70E-09	0.0001	0.0057	+	None	None
RP11-497H16.9 (0)	rs7378507	5.60E-11	0.0001	0.0057	-	None	None
ADAM20P1 (1)	rs112189666	1.80E-08	0.0001	0.0057	+	None	None
HYI (1)	rs1964952	8.50E-08	0.0001	0.0057	-	None	None
ST7 (3)	rs12706139	2.60E-09	0.0001	0.0057	+	Enhancer	Enhancer
NCBP2-AS2 (0)	rs1147242	1.70E-11	0.0001	0.0057	-	Transcription	Transcription

APBB3 (18)	rs778589	2.40E-08	0.0001	0.0057	+	None	None
ABHD12 (10)	rs6132845	5.60E-08	0.0001	0.0057	+	Enhancer	None
MGC10955 (0)	rs111615715	1.30E-09	0.0001	0.0057	+	None	None
MFGE8 (12)	rs71403963	2.40E-08	0.0001	0.0057	+	Repressed	None
SETD9 (0)	rs33329	5.00E-08	0.0001	0.0057	+	Enhancer	Enhancer
GSTO2 (4)	rs568526	2.50E-17	0.0001	0.0057	+	None	Enhancer
PEX11A (1)	rs72754562	5.80E-08	0.0001	0.0057	+	None	None
SETD4 (4)	rs2835232	1.10E-09	0.0001	0.0057	-	None	Repressed
CTC-459F4.1 (0)	rs1024728	8.60E-08	0.0001	0.0057	-	Transcription	Promoter
CLUAP1 (12)	rs12933120	1.60E-11	0.0001	0.0057	+	Transcription	None
TYW1 (14)	rs13228667	1.20E-18	0.0001	0.0057	-	None	None
GCNT1 (5)	rs55720739	2.00E-18	0.0001	0.0057	+	None	None
MRPL43 (0)	rs11190786	4.00E-16	0.0001	0.0057	+	Promoter	Promoter
RP11-585P4.5 (0)	rs11180535	2.90E-07	0.0001	0.0057	-	None	None
STX8 (11)	rs5017637	6.60E-10	0.0001	0.0057	-	None	None
RGS14 (12)	rs10051765	9.70E-16	0.0001	0.0057	-	None	None
GFM2 (1)	rs4425487	1.80E-14	0.0001	0.0057	-	None	None
CTD-2228K2.7 (2)	rs1053247	5.60E-10	0.0001	0.0057	+	Enhancer	Enhancer
ZSCAN31 (10)	rs2622320	1.90E-08	0.0001	0.0057	+	Transcription	Transcription
GRAMD3 (7)	rs62394179	1.10E-12	0.0001	0.0057	+	Transcription	None
C10orf54 (5)	rs12248205	7.60E-08	0.0001	0.0057	-	None	Enhancer
FTCDNL1 (0)	rs13008446	1.50E-18	0.0001	0.0057	-	None	None
RP11-173B14.5 (1)	rs7995740	2.60E-08	0.0001	0.0057	-	None	None
CDC42BPA (39)	rs7549619	1.30E-07	0.0001	0.0057	-	Enhancer	Enhancer
ITGB1 (0)	rs2484717	8.00E-09	0.0001	0.0057	-	None	None
RHD (10)	rs72660908	2.40E-20	0.0001	0.0057	-	None	None
ELMSAN1 (9)	rs12434080	6.80E-09	0.0001	0.0057	+	None	None
SLC44A5 (2)	rs11804097	6.60E-09	0.0001	0.0057	-	None	None
CTD-2017D11.1 (0)	rs73522440	4.30E-12	0.0001	0.0057	+	None	Transcription
TMEM161B-AS1 (7)	rs113828108	3.50E-08	0.0001	0.0057	+	None	None
ORMDL1 (0)	rs6434361	8.90E-10	0.0001	0.0057	-	Enhancer	Enhancer
FNBP4 (18)	rs74927941	6.30E-09	0.0001	0.0057	+	Transcription	Transcription
ARPC5L (0)	rs12375547	1.10E-17	0.0001	0.0057	+	None	None
DDX24 (0)	rs4905151	1.10E-19	0.0001	0.0057	-	Enhancer	Enhancer
CCDC173 (1)	rs148348800	1.70E-09	0.0001	0.0057	-	Transcription	None
FAM182B (0)	rs6050988	4.10E-08	0.0001	0.0057	+	None	None
FAM72B (0)	rs1890662	1.30E-08	0.0001	0.0057	+	None	None
WBP2 (5)	rs9894244	1.80E-10	0.0001	0.0057	+	None	None
TIMM21 (5)	rs17088884	6.80E-10	0.0001	0.0057	-	Transcription	None
UBXN2B (9)	rs143584397	4.60E-08	0.0001	0.0057	-	None	None
MTHFSD (0)	rs116869463	1.00E-16	0.0001	0.0057	-	Enhancer	Enhancer
RGPD8 (0)	rs138513560	2.50E-22	0.0001	0.0057	-	None	None
RP11-661A12.12 (0)	rs2242085	1.10E-11	0.0001	0.0057	-	Promoter	Promoter
GSTM3 (0)	rs4970777	2.30E-28	0.0001	0.0057	+	Insulator	None
NDUFS7 (3)	rs12983370	2.10E-09	0.0001	0.0057	+	None	None
YBX3 (4)	rs10845208	1.10E-11	0.0001	0.0057	-	None	None
HMGXB3 (18)	rs2228422	1.70E-07	0.0001	0.0057	-	None	None
NBPF3 (21)	rs4654748	8.40E-15	0.0001	0.0057	-	None	None
GSTT2B (0)	rs12483950	2.10E-10	0.0001	0.0057	-	None	None
SLC25A41 (0)	rs1141220	1.90E-13	0.0001	0.0057	-	None	None
RNASEH2B-AS1 (1)	rs2441	8.80E-11	0.0001	0.0057	-	Enhancer	Enhancer
CLPSL2 (1)	rs7744785	7.40E-08	0.0001	0.0057	+	Repressed	Promoter
KCNJ16 (3)	rs4968795	3.60E-20	0.0001	0.0057	+	None	Enhancer
FAM228A (1)	rs78711174	2.90E-08	0.0001	0.0057	+	Transcription	Transcription
KRT40 (1)	rs2271277	1.80E-10	0.0001	0.0057	-	None	None
LNP1 (0)	rs35925258	5.20E-08	0.0001	0.0057	-	Enhancer	None
PRKD2 (18)	rs314672	8.30E-10	0.0001	0.0057	-	Transcription	Transcription
ZNF93 (1)	rs1019280	5.40E-15	0.0001	0.0057	+	None	None
GSKIP (1)	rs930519	4.20E-10	0.0001	0.0057	+	None	None
WFDC3 (3)	rs36051332	1.30E-08	0.0001	0.0057	+	None	None
RSBN1L-AS1 (3)	rs17807185	9.10E-10	0.0001	0.0057	-	None	Enhancer
PSORS1C3 (0)	rs115404146	3.20E-09	0.0001	0.0057	+	Transcription	Transcription
RHCE (6)	rs35176086	2.70E-08	0.0001	0.0057	-	None	None
EDNRA (4)	rs5342	4.90E-10	0.0001	0.0057	+	None	Repressed
LMCD1 (1)	rs9836284	9.10E-09	0.0001	0.0057	-	Transcription	Transcription
AC074286.1 (7)	rs1453376	3.70E-25	0.0001	0.0057	-	None	None
CDK11B (6)	rs1534951	1.80E-07	0.0001	0.0057	+	None	None
AC113607.1 (3)	rs59526608	3.00E-08	0.0001	0.0057	+	None	Enhancer
ERGIC3 (4)	rs7261820	2.50E-09	0.0001	0.0057	+	None	None
SAMD4B (1)	rs62119681	1.70E-09	0.0001	0.0057	-	Enhancer	None
KIAA1919 (3)	rs9487605	2.60E-08	0.0001	0.0057	-	Enhancer	None
SPATA20 (1)	rs8076632	4.30E-09	0.0001	0.0057	-	Enhancer	Enhancer
NUS1 (1)	rs80196932	1.50E-09	0.0001	0.0057	+	Promoter	Promoter
MON1B (2)	rs284936	1.10E-08	0.0001	0.0057	-	None	None
NAMPT (12)	rs11764463	4.10E-11	0.0001	0.0057	-	None	None
FAM21C (12)	rs10751385	2.30E-14	0.0001	0.0057	-	None	Transcription
MRPL21 (0)	rs678904	1.70E-15	0.0001	0.0057	-	None	None
PI4KA (4)	rs5760217	8.50E-31	0.0001	0.0057	-	Transcription	None
EXOSC7 (4)	rs4683031	6.60E-08	0.0001	0.0057	-	None	Enhancer
PSMD13 (0)	rs10902107	1.20E-09	0.0001	0.0057	-	None	None
BANF1 (0)	rs6591195	5.70E-11	0.0001	0.0057	+	Enhancer	Repressed
ASB1 (0)	rs508486	7.10E-08	0.0001	0.0057	-	Transcription	Transcription
LRRC37A2 (1)	rs117692492	3.40E-20	0.0001	0.0057	-	None	None
AC017099.3 (16)	rs2276644	4.10E-10	0.0001	0.0057	+	None	None
DTWD2 (0)	rs7720231	1.20E-07	0.0001	0.0057	-	Enhancer	None
MAMDC2-AS1 (12)	rs140010888	4.10E-11	0.0001	0.0057	+	None	None
ZNF98 (0)	rs2195966	1.00E-07	0.0001	0.0057	-	None	None
SPPL2B (19)	rs757321	3.30E-10	0.0001	0.0057	-	Promoter	Repressed
CBR4 (8)	rs13140828	8.10E-11	0.0001	0.0057	-	Transcription	Enhancer
CDSN (0)	rs3094199	1.90E-23	0.0001	0.0057	-	None	Enhancer
METAP1 (1)	rs9685029	9.90E-15	0.0001	0.0057	+	None	None
ANKLE2 (11)	rs10781644	1.60E-08	0.0001	0.0057	-	Enhancer	None
HLA-DQB1 (0)	rs9274284	2.10E-33	0.0001	0.0057	-	Repressed	None
IRF5 (12)	rs3823536	5.60E-13	0.0001	0.0057	+	Repressed	None

IFNAR2 (5)	rs17860115	1.70E-08	0.0001	0.0057	-	Promoter	Promoter
TVP23B (3)	rs4244595	7.70E-08	0.0001	0.0057	-	None	None
UQCRC2 (6)	rs460473	9.30E-08	0.0001	0.0057	-	Repressed	Repressed
CA8 (9)	rs1904780	1.10E-07	0.0001	0.0057	-	None	None
SRA1 (2)	rs11743782	1.10E-39	0.0001	0.0057	-	None	None
SLC25A1 (0)	rs1780637	7.80E-19	0.0001	0.0057	-	None	None
IKBIP (1)	rs11109593	1.50E-08	0.0001	0.0057	+	None	None
ZMIZ1-AS1 (6)	rs10824709	1.60E-12	0.0001	0.0057	-	Enhancer	Enhancer
HBS1L (0)	rs4388304	5.10E-11	0.0001	0.0057	-	Transcription	Transcription
WDSUB1 (9)	rs2159876	6.70E-09	0.0001	0.0057	-	None	Transcription
NDUFB2 (6)	rs28533681	4.60E-09	0.0001	0.0057	+	None	None
LYNX1 (3)	rs2717590	2.60E-18	0.0001	0.0057	-	Repressed	Repressed
C1QL3 (0)	rs7909832	1.70E-17	0.0001	0.0057	+	None	None
LINC00667 (0)	rs35213903	9.20E-09	0.0001	0.0057	+	None	None
STX2 (0)	rs191866037	3.30E-09	0.0001	0.0057	-	None	None
OPTN (18)	rs17512962	1.80E-08	0.0001	0.0057	-	Transcription	Transcription
ABCC5 (0)	rs3792585	7.00E-09	0.0001	0.0057	+	Transcription	None
RPLP2 (1)	rs7479101	5.80E-10	0.0001	0.0057	+	Transcription	None
AC091729.9 (1)	rs6978842	2.20E-27	0.0001	0.0057	-	None	None
AC005562.1 (0)	rs143813433	1.70E-18	0.0001	0.0057	+	Promoter	Promoter
ATP6V0A2 (3)	rs10082815	2.00E-07	0.0001	0.0057	-	Repressed	None
KLHDC7A (0)	rs2992735	1.70E-08	0.0001	0.0057	+	None	None
RWDD3 (1)	rs146672728	6.90E-08	0.0001	0.0057	-	None	None
CINP (3)	rs11629266	3.00E-15	0.0001	0.0057	-	Transcription	Transcription
NDUFA10 (12)	rs11684044	3.30E-08	0.0001	0.0057	-	None	None
TUBG2 (1)	rs2292750	3.60E-14	0.0001	0.0057	-	Promoter	Promoter
ZNF506 (5)	rs11085277	2.60E-19	0.0001	0.0057	+	Transcription	None
NDST1 (19)	rs3797621	4.00E-07	0.0001	0.0057	+	Enhancer	Enhancer
ACP6 (3)	rs2275552	1.60E-07	0.0001	0.0057	+	Transcription	Transcription
CTD-2336O2.1 (0)	rs6558533	7.90E-11	0.0001	0.0057	+	Enhancer	Enhancer
CEP192 (17)	rs8089123	3.40E-09	0.0001	0.0057	-	None	Transcription
CCDC82 (9)	rs11021552	1.60E-14	0.0001	0.0057	+	Transcription	Transcription
PCDHB8 (0)	rs11741863	3.00E-15	0.0001	0.0057	+	None	Promoter
ARL16 (0)	rs62077189	1.00E-13	0.0001	0.0057	-	Transcription	None
RPF2 (10)	rs12527783	1.50E-08	0.0001	0.0057	+	None	None
ZNF273 (5)	rs73126387	2.10E-08	0.0001	0.0057	+	None	None
RP1-276N6.2 (1)	rs569919	3.90E-08	0.0001	0.0057	+	Repressed	None
RP11-1143G9.4 (0)	rs12812860	1.90E-12	0.0001	0.0057	-	None	None
ARHGAP40 (8)	rs76524484	7.60E-09	0.0001	0.0057	-	None	None
SPATA5L1 (0)	rs1974981	1.80E-08	0.0001	0.0057	+	None	None
SNHG16 (2)	rs12944474	3.60E-10	0.0001	0.0057	-	None	Enhancer
NMD3 (1)	rs28384382	4.30E-15	0.0001	0.0057	-	Promoter	Promoter
ULK3 (2)	rs12442901	2.20E-08	0.0001	0.0057	-	Enhancer	Enhancer
AC018642.1 (0)	rs966013	1.30E-07	0.0001	0.0057	-	Enhancer	Enhancer
ALG1L (1)	rs71327750	1.00E-07	0.0001	0.0057	-	None	None
ACOT4 (1)	rs3742819	4.80E-09	0.0001	0.0057	+	Promoter	Promoter
SEC31A (31)	rs10011004	6.70E-09	0.0001	0.0057	+	None	None
PAQR6 (0)	rs12128955	1.30E-11	0.0001	0.0057	-	Transcription	Transcription
OXA1L (9)	rs200470407	5.30E-27	0.0001	0.0057	+	Transcription	Transcription
CPT1C (10)	rs61189653	7.90E-09	0.0001	0.0057	-	None	None
LINC00610 (0)	rs149148062	2.70E-08	0.0001	0.0057	-	Enhancer	None
XXbac-BPG299F13.17 (0)	rs138787908	9.30E-13	0.0001	0.0057	-	Enhancer	Promoter
PILRA (1)	rs190448056	9.50E-21	0.0001	0.0057	+	Promoter	Promoter
STPG1 (6)	rs72880692	3.80E-13	0.0001	0.0057	-	Enhancer	Enhancer
SLC5A11 (11)	rs2303083	6.70E-08	0.0001	0.0057	+	Transcription	Transcription
CCL4L2 (0)	rs2002481	9.40E-08	0.0001	0.0057	-	Repressed	None
MYOM2 (38)	rs2280902	1.60E-09	0.0001	0.0057	-	Enhancer	None
HLA-DRA (4)	rs111232699	7.20E-09	0.0001	0.0057	-	None	None
RABL2A (0)	rs12711778	1.50E-09	0.0001	0.0057	-	Insulator	None
HAVCR1 (5)	rs1948759	4.00E-09	0.0001	0.0057	-	None	None
ZNF718 (2)	rs28641663	7.40E-23	0.0001	0.0057	+	None	None
PILRB (14)	rs10085549	1.80E-23	0.0001	0.0057	+	None	None
FARP1 (40)	rs7988345	1.20E-10	0.0001	0.0057	+	Enhancer	Transcription
CCDC25 (0)	rs17477565	1.40E-07	0.0001	0.0057	+	Enhancer	Transcription
KANSL1-AS1 (0)	rs118152943	4.10E-12	0.0001	0.0057	+	Promoter	Promoter
ZNF593 (1)	rs7546500	1.20E-08	0.0001	0.0057	-	None	Enhancer
ZNF554 (4)	rs2260414	1.20E-07	0.0001	0.0057	+	Transcription	None
STK38L (3)	rs10842906	2.70E-18	0.0001	0.0057	+	None	None
SDC2 (6)	rs2439512	7.80E-09	0.0001	0.0057	-	Enhancer	Enhancer
C10orf137 (23)	rs76339414	1.40E-07	0.0001	0.0057	-	None	None
EIF3E (11)	rs2597658	1.30E-07	0.0001	0.0057	+	Enhancer	Enhancer
AC005532.5 (4)	rs1638210	1.30E-14	0.0001	0.0057	-	None	None
PSORS1C1 (0)	rs3094221	8.70E-08	0.0001	0.0057	+	Repressed	Promoter
PAK1 (17)	rs34025917	4.70E-08	0.0001	0.0057	-	None	None
RP11-495K9.6 (1)	rs7313017	3.20E-13	0.0001	0.0057	-	None	None
FANK1 (1)	rs7097500	4.30E-14	0.0001	0.0057	-	Enhancer	None
RNF149 (1)	rs13031025	7.80E-21	0.0001	0.0057	+	Enhancer	None
TIMM22 (3)	rs3744750	1.20E-10	0.0001	0.0057	-	Enhancer	Enhancer
LINC00525 (1)	rs5004323	3.80E-14	0.0001	0.0057	-	None	None
SGCE (3)	rs35818859	1.30E-11	0.0001	0.0057	-	Transcription	None
CDK11A (6)	rs28541555	1.70E-15	0.0001	0.0057	+	None	Enhancer
MICA (0)	rs114609834	6.70E-11	0.0001	0.0057	-	None	None
LINS (4)	rs7183556	9.50E-12	0.0001	0.0057	-	None	None
PTPRD (18)	rs1323489	1.60E-07	0.0001	0.0057	+	None	None
FAM207A (7)	rs928305	5.80E-10	0.0001	0.0057	-	None	None
SLC35G2 (0)	rs2343662	2.10E-09	0.0001	0.0057	-	None	Transcription
ZNF738 (1)	rs12974222	4.00E-09	0.0001	0.0057	+	None	None
IQCB1 (0)	rs80047370	2.10E-14	0.0001	0.0057	+	Transcription	Transcription
CDH18 (0)	rs1703069	5.70E-10	0.0001	0.0057	+	Enhancer	Enhancer
RP11-96L14.7 (0)	rs3748854	1.00E-07	0.0001	0.0057	-	None	Enhancer
PPIE (9)	rs11809515	6.60E-36	0.0001	0.0057	+	Enhancer	Enhancer
CTD-2583A14.8 (0)	rs11668314	1.90E-14	0.0001	0.0057	-	Transcription	Promoter
CLEC18C (10)	rs3192882	8.90E-13	0.0001	0.0057	+	Transcription	None
NARG2 (0)	rs7179558	1.70E-09	0.0001	0.0057	-	Enhancer	Enhancer

CARS (19)	rs438384	4.10E-16	0.0001	0.0057	-	Transcription	None
U2AF1L4 (1)	rs7260184	1.60E-25	0.0001	0.0057	+	Enhancer	Enhancer
ACPL2 (3)	rs7636976	6.20E-11	0.0001	0.0057	+	Promoter	Promoter
RP11-539L10.3 (0)	rs7662433	4.90E-08	0.0001	0.0057	-	Promoter	Enhancer
ARFGAP3 (4)	rs5758989	2.20E-16	0.0001	0.0057	+	Enhancer	Promoter
CD59 (4)	rs1718066	2.70E-13	0.0001	0.0057	-	Enhancer	Promoter
RPS10 (3)	rs79954907	1.60E-10	0.0001	0.0057	+	None	None
DMBT1 (11)	rs11528750	6.30E-12	0.0001	0.0057	+	None	Repressed
KCNS3 (4)	rs1461951	4.10E-09	0.0001	0.0057	+	None	None
AC108488.3 (0)	rs4583476	4.20E-08	0.0001	0.0057	-	None	None
PHC1 (8)	rs28521458	1.50E-10	0.0001	0.0057	+	None	None
SIGLEC11 (7)	rs7507392	2.50E-10	0.0001	0.0057	+	None	None
NADK (4)	rs35301881	4.30E-09	0.0001	0.0057	+	None	None
BCR (30)	rs131684	8.60E-20	0.0001	0.0057	-	Transcription	Transcription
M6PR (5)	rs4883201	1.30E-12	0.0001	0.0057	-	None	None
CLEC18A (12)	rs4985394	1.70E-10	0.0001	0.0057	-	None	None
C10orf107 (0)	rs12219505	1.90E-08	0.0001	0.0057	+	None	None
GPX7 (1)	rs3753754	2.40E-10	0.0001	0.0057	-	Enhancer	Enhancer
DNAJC22 (0)	rs7955245	6.40E-12	0.0001	0.0057	-	None	None
RAB2A (4)	rs6471888	5.60E-12	0.0001	0.0057	-	Enhancer	Enhancer
RP1-29C18.8 (0)	rs9616694	1.40E-10	0.0001	0.0057	-	Repressed	Repressed
HLA-F (6)	rs151024893	6.30E-08	0.0001	0.0057	-	None	None
CPNE3 (4)	rs13270090	7.80E-10	0.0001	0.0057	-	Enhancer	Promoter
CERS2 (0)	rs63151761	6.50E-09	0.0001	0.0057	-	Enhancer	Promoter
STOX1 (2)	rs34657772	3.70E-08	0.0001	0.0057	+	None	None
DMKN (13)	rs4254439	4.90E-28	0.0001	0.0057	+	None	Enhancer
ZNF665 (5)	rs7253428	9.60E-10	0.0001	0.0057	-	Transcription	None
KLHL7-AS1 (0)	rs1985769	3.30E-15	0.0001	0.0057	+	None	None
RP11-452K12.7 (0)	rs11189182	1.10E-11	0.0001	0.0057	+	Transcription	Transcription
SLIRP (1)	rs8018963	2.60E-08	0.0001	0.0057	+	None	None
AL592284.1 (0)	rs112298300	4.80E-29	0.0001	0.0057	+	Enhancer	Enhancer
ANO5 (0)	rs4922978	1.70E-09	0.0001	0.0057	-	None	Enhancer
PWP2 (16)	rs2073434	9.80E-09	0.0001	0.0057	-	Enhancer	Enhancer
POLRMT (21)	rs8112348	3.30E-09	0.0001	0.0057	-	None	None
MECOM (5)	rs4955680	3.80E-07	0.0001	0.0057	+	None	None
PALM3 (0)	rs36014207	2.00E-15	0.0001	0.0057	-	Promoter	Promoter
PLGLB1 (0)	rs150897198	2.00E-08	0.0001	0.0057	+	None	None
TLDC1 (11)	rs433630	1.80E-13	0.0001	0.0057	-	Enhancer	Promoter
RPL14 (0)	rs4974077	1.50E-12	0.0001	0.0057	+	None	None
RP11-734I18.1 (0)	rs1589656	4.60E-10	0.0001	0.0057	-	None	None
ANKRD10 (8)	rs7332021	6.30E-19	0.0001	0.0057	-	None	None
RIC8A (0)	rs142580041	3.60E-08	0.0001	0.0057	-	Transcription	Transcription
PAX8 (0)	rs1139015	3.10E-13	0.0001	0.0057	-	Enhancer	Enhancer
FAH (15)	rs16971828	4.10E-11	0.0001	0.0057	-	None	None
DPY19L2 (0)	rs3913888	3.70E-10	0.0001	0.0057	-	None	None
ABCA10 (17)	rs1011789	1.60E-10	0.0001	0.0057	+	None	None
PRKAG1 (11)	rs10875911	4.90E-17	0.0001	0.0057	-	None	None
TMEM126A (0)	rs1046216	1.70E-26	0.0001	0.0057	-	Transcription	Transcription
RP11-77P16.4 (0)	rs73210765	2.40E-11	0.0001	0.0057	-	None	None
RP11-47I22.1 (1)	rs72716753	1.90E-08	0.0001	0.0057	-	None	None
CKAP2 (6)	rs7990581	1.80E-08	0.0001	0.0057	-	None	None
RP11-293A21.1 (0)	rs13105390	1.10E-08	0.0001	0.0057	-	None	Enhancer
SMDT1 (0)	rs133381	1.30E-10	0.0001	0.0057	+	Promoter	Promoter
LIMD1 (8)	rs62242182	3.20E-08	0.0001	0.0057	+	Enhancer	Enhancer
RP11-259O2.1 (2)	rs28529452	1.40E-12	0.0001	0.0057	-	Repressed	Repressed
MYO5B (13)	rs200219597	6.50E-12	0.0001	0.0057	+	Enhancer	Enhancer
CDH2 (1)	rs1373138	3.90E-08	0.0001	0.0057	-	None	None
ETFDH (0)	rs57327548	8.40E-08	0.0001	0.0057	-	None	None
C17orf76-AS1 (5)	rs12602006	4.90E-15	0.0001	0.0057	+	None	None
ZNF175 (1)	rs4308054	4.80E-09	0.0001	0.0057	+	Repressed	Repressed
METAP2 (4)	rs66765539	1.60E-11	0.0001	0.0057	+	Transcription	Transcription
PEX5L (0)	rs9825224	6.30E-08	0.0001	0.0057	+	None	None
C11orf54 (2)	rs1284141	6.40E-08	0.0001	0.0057	-	None	Enhancer
RP4-756H11.3 (2)	rs4718363	4.50E-18	0.0001	0.0057	-	None	None
LGMN (14)	rs79132724	3.10E-11	0.0001	0.0057	+	None	None
AC002472.13 (3)	rs641901	8.80E-08	0.0001	0.0057	+	None	None
NPC1 (1)	rs7233671	6.60E-11	0.0001	0.0057	+	Enhancer	Promoter
ZNF589 (0)	rs28759711	1.30E-09	0.0001	0.0057	-	None	None
IST1 (8)	rs34692271	1.00E-10	0.0001	0.0057	-	None	None
KLHL24 (1)	rs13078822	7.50E-08	0.0001	0.0057	-	Enhancer	Enhancer
METTL2B (1)	rs2288557	5.20E-17	0.0001	0.0057	-	Promoter	Promoter
CLDN23 (0)	rs7839473	3.70E-09	0.0001	0.0057	-	None	Repressed
RBM43 (4)	rs289924	4.70E-11	0.0001	0.0057	+	None	None
ARL17B (3)	rs2074404	2.10E-08	0.0001	0.0057	-	None	None
GREB1 (11)	rs4668732	4.70E-08	0.0001	0.0057	+	None	Transcription
RP3-414A15.2 (1)	rs758053	1.40E-08	0.0001	0.0057	-	None	None
THOC3 (2)	rs62389969	2.90E-10	0.0001	0.0057	+	None	None
AC002451.3 (0)	rs10085780	3.30E-11	0.0001	0.0057	+	Promoter	Promoter
NDUFV3 (3)	rs4148974	3.70E-08	0.0001	0.0057	-	Transcription	Transcription
HSD17B12 (14)	rs1518822	6.00E-13	0.0001	0.0057	-	None	None
RP4-545C24.1 (1)	rs714443	8.00E-22	0.0001	0.0057	-	None	None
NME7 (0)	rs12028817	2.30E-07	0.0001	0.0057	+	Enhancer	Enhancer
NSUN4 (0)	rs55921163	9.50E-11	0.0001	0.0057	-	None	None
VEZT (18)	rs116848449	4.00E-25	0.0001	0.0057	+	None	Transcription
C5 (22)	rs10760132	6.30E-09	0.0001	0.0057	-	None	None
BTNL3 (7)	rs72494581	4.10E-13	0.0001	0.0057	+	Insulator	None
WDR72 (20)	rs62005963	9.50E-08	0.0001	0.0057	+	None	None
OAS1 (5)	rs10774671	2.30E-16	0.0001	0.0057	+	Transcription	None
NADSYN1 (12)	rs4944946	4.40E-10	0.0001	0.0057	+	Promoter	Promoter
CWF19L1 (0)	rs2862954	2.80E-10	0.0001	0.0057	+	Transcription	Transcription
ASAH2 (0)	rs4350318	2.70E-22	0.0001	0.0057	+	None	None
NT5C2 (22)	rs1971589	2.30E-09	0.0001	0.0057	-	None	None
PPFIA2 (24)	rs12366599	1.80E-08	0.0001	0.0057	+	None	None
USP6 (14)	rs2585266	6.30E-09	0.0001	0.0057	+	Transcription	Transcription

LINC00969 (1)	rs76007885	9.80E-12	0.0001	0.0057	-	Enhancer	Enhancer
IFI27 (5)	rs7141881	8.00E-19	0.0001	0.0057	-	None	None
SFT2D1 (7)	rs2343590	1.40E-13	0.0001	0.0057	-	None	None
SUSD2 (8)	rs5760089	1.30E-07	0.0001	0.0057	+	None	None
PDLIM3 (2)	rs6823554	1.10E-07	0.0001	0.0057	-	None	None
CAMTA1 (4)	rs12711514	6.40E-11	0.0001	0.0057	-	None	Enhancer
TXNRD1 (10)	rs10861185	5.90E-23	0.0001	0.0057	-	None	None
ARL17A (1)	rs190544400	6.90E-10	0.0001	0.0057	+	Enhancer	Enhancer
CCNB1IP1 (6)	rs7155699	7.30E-18	0.0001	0.0057	-	Enhancer	Transcription
MBIP (11)	rs4083518	1.80E-14	0.0001	0.0057	+	Promoter	Promoter
WARS (16)	rs1998902	3.20E-34	0.0001	0.0057	-	Transcription	Transcription
PCAT6 (1)	rs12751404	1.60E-08	0.0001	0.0057	-	None	None
DTX2 (14)	rs150952334	2.20E-10	0.0001	0.0057	+	None	None
TMEM44-AS1 (2)	rs999941	4.60E-14	0.0001	0.0057	+	Enhancer	None
FGGY (29)	rs591512	7.80E-08	0.0001	0.0057	-	None	None
PGD (5)	rs4846218	7.10E-08	0.0001	0.0057	+	None	None
FAM90A26 (6)	rs10031337	6.50E-17	0.0001	0.0057	-	None	None
ZNF585A (1)	rs10413934	3.60E-10	0.0001	0.0057	+	Transcription	Promoter
MBTPS1 (23)	rs3785023	1.60E-09	0.0001	0.0057	+	Transcription	Transcription
FYN (22)	rs9387034	8.00E-09	0.0001	0.0057	+	None	None
BCL2L13 (5)	rs5747326	9.30E-12	0.0001	0.0057	-	Transcription	None
DECR2 (2)	rs3743893	1.60E-08	0.0001	0.0057	-	Transcription	None
RPL28 (1)	rs17700376	3.10E-09	0.0001	0.0057	-	Promoter	Promoter
CRELD2 (7)	rs138838	4.70E-10	0.0001	0.0057	-	Transcription	Transcription
SLC35E2 (5)	rs6699975	1.10E-11	0.0001	0.0057	+	Transcription	Transcription
DLG5 (17)	rs10762764	5.80E-08	0.0001	0.0057	+	Transcription	Transcription
CTD-2203K17.1 (1)	rs78735287	2.00E-14	0.0001	0.0057	-	None	None
SPRR1B (0)	rs512198	5.00E-10	0.0001	0.0057	-	None	None
GIPC1 (11)	rs8110419	2.80E-08	0.0001	0.0057	-	None	None
SNRPB2 (0)	rs2073055	2.20E-08	0.0001	0.0057	-	Promoter	Promoter
ANAPC4 (25)	rs3756207	2.20E-07	0.0001	0.0057	-	Enhancer	None
SMARCC2 (22)	rs35616842	1.60E-08	0.0001	0.0057	-	Enhancer	Enhancer
POLR2L (0)	rs74194353	5.00E-08	0.0001	0.0057	-	Promoter	Promoter
FAM182A (10)	rs845778	3.80E-10	0.0001	0.0057	-	None	None
AP1S1 (5)	rs10953325	3.10E-14	0.0001	0.0057	-	Enhancer	Enhancer
KIF9 (22)	rs4858894	1.00E-09	0.0001	0.0057	+	Enhancer	Enhancer
LEFTY1 (0)	rs1223246	3.40E-08	0.0001	0.0057	-	None	None
GOLGA6L4 (5)	rs609513	6.00E-12	0.0001	0.0057	+	None	None
RP11-567L7.5 (0)	rs9905500	1.70E-11	0.0001	0.0057	+	Enhancer	Transcription
RP4-724E13.2 (1)	rs1046964	1.90E-23	0.0001	0.0057	+	None	None
STX18 (0)	rs7686055	1.10E-11	0.0001	0.0057	-	None	None
AP000344.3 (0)	rs2154589	6.90E-08	0.0001	0.0057	+	None	None
CCL3L1 (0)	rs34914791	2.10E-09	0.0001	0.0057	-	None	None
AC004540.5 (6)	rs1229669	1.30E-07	0.0001	0.0057	-	None	None
CTD-2054N24.2 (2)	rs12904160	1.40E-11	0.0001	0.0057	-	None	None
RP11-294O2.2 (0)	rs4516676	3.10E-12	0.0001	0.0057	-	None	None
PPFIA3 (12)	rs4802556	6.20E-07	0.0001	0.0057	-	Enhancer	Enhancer
TNFRSF1A (4)	rs887477	3.10E-08	0.0001	0.0057	+	Enhancer	Enhancer
DPP4 (26)	rs12622595	1.00E-06	0.0001	0.0057	-	None	None
RPH3AL (15)	rs12951485	6.40E-10	0.0001	0.0057	-	Enhancer	Promoter
NUP54 (9)	rs11724543	4.20E-21	0.0001	0.0057	-	None	None
MRPL42 (7)	rs12811748	2.20E-34	0.0001	0.0057	-	None	None
C16orf45 (4)	rs2071332	3.00E-08	0.0001	0.0057	-	Transcription	Transcription
FUT10 (0)	rs12681435	1.90E-07	0.0001	0.0057	-	None	Transcription
PYROXD1 (10)	rs4762826	2.40E-17	0.0001	0.0057	-	Transcription	Transcription
IFI27L2 (1)	rs2273514	9.30E-08	0.0001	0.0057	-	Promoter	Promoter
ASAH2B (5)	rs4471367	1.40E-09	0.0001	0.0057	-	None	None
FLG (0)	rs17596572	7.60E-14	0.0001	0.0057	-	None	None
KLC2 (0)	rs11227447	3.90E-09	0.0001	0.0057	+	Enhancer	Enhancer
PPP1R3B (1)	rs330924	4.00E-10	0.0001	0.0057	-	Transcription	Transcription
CUTA (0)	rs146118878	3.00E-09	0.0001	0.0057	-	None	None
RBM7 (0)	rs3741302	2.20E-12	0.0001	0.0057	-	Promoter	Promoter
PBX2 (3)	rs115414351	8.10E-08	0.0001	0.0057	+	None	Repressed
COQ5 (8)	rs10774553	1.20E-08	0.0001	0.0057	+	Transcription	Transcription
KIN (2)	rs111705106	4.80E-09	0.0001	0.0057	+	Transcription	Transcription
SGCA (1)	rs2586478	6.70E-09	0.0001	0.0057	-	None	None
INPP1 (2)	rs2067454	9.90E-09	0.0001	0.0057	-	Transcription	None
RP11-184M15.2 (2)	rs904477	2.20E-08	0.0001	0.0057	+	Enhancer	Enhancer
C7orf50 (6)	rs10257761	7.80E-11	0.0001	0.0057	-	None	None
PPA2 (0)	rs13108489	5.60E-24	0.0001	0.0057	-	Transcription	Transcription
MAPT (9)	rs117120492	9.60E-08	0.0001	0.0057	+	None	Enhancer
CTC-241F20.3 (1)	rs12984612	2.70E-08	0.0001	0.0057	-	Promoter	Promoter
C9orf135 (0)	rs17518268	2.00E-08	0.0001	0.0057	+	Promoter	Promoter
RP11-755F10.1 (0)	rs580891	9.00E-11	0.0001	0.0057	+	None	None
SNIP1 (2)	rs4653300	1.60E-11	0.0001	0.0057	+	Enhancer	Enhancer
DNAJC15 (0)	rs2281778	3.40E-19	0.0001	0.0057	-	Promoter	Promoter
ACSF3 (4)	rs6500529	2.10E-15	0.0001	0.0057	-	None	Transcription
CASP8 (15)	rs146303601	4.40E-08	0.0001	0.0057	+	None	None
TPRG1L (4)	rs2760321	4.30E-08	0.0001	0.0057	-	Transcription	None
TMEM17 (3)	rs6713096	6.40E-09	0.0001	0.0057	-	Enhancer	Enhancer
NUDT19 (2)	rs71351189	5.20E-08	0.0001	0.0057	-	None	None
SPRR1A (0)	rs11580383	1.50E-11	0.0001	0.0057	+	None	None
NUDT2 (1)	rs77025004	1.10E-13	0.0001	0.0057	-	None	None
ZAP70 (13)	rs10169777	5.60E-08	0.0001	0.0057	-	Transcription	None
XRRA1 (1)	rs7105085	8.20E-14	0.0001	0.0057	+	Transcription	Transcription
GLTP (3)	rs11064860	2.10E-07	0.0001	0.0057	+	None	None
CACNA1H (21)	rs2753325	9.00E-13	0.0001	0.0057	-	Transcription	None
POLI (13)	rs12454487	4.10E-10	0.0001	0.0057	-	None	None
CLMN (1)	rs3180753	4.20E-12	0.0001	0.0057	+	None	Transcription
SYCE1L (1)	rs8398	8.90E-08	0.0001	0.0057	+	Transcription	Transcription
ASNSD1 (4)	rs1233302	1.10E-07	0.0001	0.0057	-	Transcription	None
SAAL1 (13)	rs10500831	4.30E-10	0.0001	0.0057	-	Transcription	None
ZNF786 (3)	rs11773822	3.40E-12	0.0001	0.0057	+	None	None
NT5C3A (0)	rs10244292	7.70E-12	0.0001	0.0057	-	None	None

CTA-445C9.15 (0)	rs4275	5.50E-13	0.0001	0.0057	+	None	None
HSPBP1 (6)	rs4806656	2.40E-22	0.0001	0.0057	-	Enhancer	Enhancer
LTBR (4)	rs10774430	3.50E-09	0.0001	0.0057	-	None	None
ACOT13 (0)	rs2143340	7.20E-08	0.0001	0.0057	-	Enhancer	Enhancer
ACO2 (6)	rs6519288	2.80E-10	0.0001	0.0057	-	None	None
RP1-283E3.8 (2)	rs28592733	2.90E-08	0.0001	0.0057	-	Repressed	Repressed
UGT2B17 (0)	rs145796316	3.70E-08	0.0001	0.0057	+	None	None
ATXN3 (1)	rs2295174	5.00E-20	0.0001	0.0057	-	Promoter	Promoter
TMEM14B (3)	rs1233893	4.50E-10	0.0001	0.0057	+	Transcription	Enhancer
ARHGEF35 (0)	rs1731353	1.40E-14	0.0001	0.0057	-	None	None
MAG (12)	rs10411704	1.10E-13	0.0001	0.0057	-	None	None
RAI1 (2)	rs9897596	5.50E-16	0.0001	0.0057	+	Enhancer	Enhancer
MTCH2 (3)	rs12361256	3.30E-07	0.0001	0.0057	+	None	None
LINC00960 (0)	rs1813383	2.40E-22	0.0001	0.0057	-	None	None
PRKCDBP (0)	rs11544764	9.20E-09	0.0001	0.0057	+	Promoter	Promoter
D2HGDH (12)	rs4425108	2.70E-10	0.0001	0.0057	-	None	Transcription
AGAP4 (0)	rs11239567	3.30E-10	0.0001	0.0057	-	Enhancer	Enhancer
LY75 (0)	rs3792198	4.50E-14	0.0001	0.0057	+	Enhancer	Enhancer
ZNF587 (0)	rs28374851	7.20E-09	0.0001	0.0057	+	Promoter	Promoter
TRAP1 (9)	rs4786429	9.90E-09	0.0001	0.0057	+	Transcription	Transcription
SPG7 (14)	rs9935289	6.80E-23	0.0001	0.0057	+	Promoter	Promoter
AC016757.3 (2)	rs6740487	1.60E-08	0.0001	0.0057	+	None	Enhancer
LL22NC03-86G7.1 (0)	rs9607347	7.00E-09	0.0001	0.0057	-	None	None
CECR1 (0)	rs17807317	4.00E-25	0.0001	0.0057	+	Promoter	Promoter
RP11-341N2.1 (0)	rs6741316	1.10E-07	0.0002	0.011	+	Enhancer	Promoter
SMU1 (0)	rs10125928	5.80E-08	0.0002	0.011	+	None	None
SLC37A1 (24)	rs368955	3.50E-08	0.0002	0.011	-	None	None
FANCD2 (39)	rs62240188	3.50E-08	0.0002	0.011	-	Repressed	Repressed
IFT74 (16)	rs10967599	1.40E-07	0.0002	0.011	+	Transcription	Transcription
SEPT2 (18)	rs62186363	2.30E-07	0.0002	0.011	+	Transcription	Transcription
SERPIND1 (2)	rs80181964	2.30E-07	0.0002	0.011	+	Enhancer	Enhancer
EFHB (15)	rs4103004	3.50E-07	0.0002	0.011	+	None	None
FXYD2 (0)	rs528431	1.50E-07	0.0002	0.011	+	None	None
SLAMF8 (2)	rs10158752	3.30E-07	0.0002	0.011	+	None	Enhancer
ACTR2 (4)	rs268858	2.10E-07	0.0002	0.011	+	None	Transcription
PLEC (33)	rs11784985	5.70E-08	0.0002	0.011	-	None	None
L47234.1 (0)	rs2965169	9.50E-08	0.0002	0.011	+	Enhancer	Enhancer
LINC00479 (0)	rs2838113	2.90E-08	0.0002	0.011	-	Transcription	Transcription
CHPT1 (9)	rs9668831	2.60E-07	0.0002	0.011	+	None	None
FRY-AS1 (0)	rs431506	2.20E-07	0.0002	0.011	+	None	None
TMCO5A (11)	rs7166638	1.40E-08	0.0002	0.011	-	None	None
MIR3188 (0)	rs7247222	2.00E-07	0.0002	0.011	+	Promoter	Promoter
RP11-439L8.3 (1)	rs1320578	9.20E-09	0.0002	0.011	-	None	None
C19orf55 (8)	rs57770499	1.00E-07	0.0002	0.011	-	None	None
AC007349.4 (0)	rs146979684	3.60E-08	0.0002	0.011	-	None	None
PTPN11 (5)	rs4767009	1.20E-07	0.0002	0.011	-	None	None
CAMK1D (10)	rs7100710	1.70E-07	0.0002	0.011	-	Transcription	Transcription
FAM120A (17)	rs76640831	3.70E-07	0.0002	0.011	-	None	None
TXNDC11 (9)	rs10492852	1.10E-07	0.0002	0.011	-	Enhancer	Enhancer
NPIPA3 (8)	rs7499403	6.90E-08	0.0002	0.011	+	None	None
BTNL8 (9)	rs114244788	5.00E-08	0.0002	0.011	-	Repressed	None
LRCH4 (0)	rs73154891	1.80E-07	0.0002	0.011	+	None	None
RP11-347C12.1 (10)	rs2911213	2.00E-07	0.0002	0.011	+	None	None
DCAF5 (3)	rs8005658	2.90E-08	0.0002	0.011	-	None	None
KITLG (6)	rs77143377	3.40E-07	0.0002	0.011	-	None	Enhancer
FLNB (26)	rs77213664	3.40E-08	0.0002	0.011	-	Transcription	Transcription
AGXT (2)	rs34977557	4.80E-08	0.0002	0.011	+	None	Enhancer
COL5A2 (39)	rs13306270	7.80E-07	0.0002	0.011	-	None	None
SAMM50 (9)	rs2281297	2.80E-08	0.0002	0.011	-	Transcription	None
CCDC158 (16)	rs113770925	1.30E-07	0.0002	0.011	-	None	None
AC069513.4 (2)	rs6583249	5.70E-08	0.0002	0.011	-	Enhancer	None
CTD-2620I22.3 (0)	rs78695861	2.80E-08	0.0002	0.011	+	Transcription	None
KLHL25 (0)	rs4843090	2.70E-07	0.0002	0.011	+	None	None
ZNF337 (3)	rs6076335	1.90E-08	0.0002	0.011	+	None	None
DNAH10 (67)	rs117516737	3.10E-07	0.0002	0.011	-	Enhancer	Enhancer
MUC22 (1)	rs2517540	2.10E-08	0.0002	0.011	-	None	None
PGAP3 (1)	rs903504	1.90E-07	0.0002	0.011	-	None	None
DDX1 (24)	rs807614	2.20E-07	0.0002	0.011	-	None	None
RP11-488L18.10 (0)	rs1771934	3.70E-07	0.0002	0.011	-	None	None
MAATS1 (10)	rs10048954	1.40E-07	0.0002	0.011	-	None	None
LGALS9C (12)	rs4324187	3.50E-07	0.0002	0.011	+	None	None
RP11-125B21.2 (10)	rs506935	2.20E-07	0.0002	0.011	+	None	Insulator
RP11-184E9.1 (0)	rs72751494	7.10E-09	0.0002	0.011	+	None	None
NIPSNAP3A (2)	rs4742922	1.70E-08	0.0002	0.011	+	Enhancer	Enhancer
MICU1 (10)	rs2921447	3.00E-09	0.0002	0.011	-	None	None
TBC1D20 (3)	rs4061743	2.90E-07	0.0002	0.011	-	Transcription	Enhancer
DIP2B (27)	rs224591	9.50E-07	0.0002	0.011	-	Transcription	Enhancer
NHP2L1 (3)	rs73176682	2.00E-07	0.0002	0.011	+	Enhancer	None
BCL9L (6)	rs7933848	4.10E-07	0.0002	0.011	-	Enhancer	Enhancer
MGAT5B (16)	rs880781	2.30E-07	0.0002	0.011	+	Repressed	Repressed
ALDH8A1 (0)	rs13191501	1.50E-07	0.0002	0.011	-	Transcription	None
CLYBL (7)	rs7335739	1.50E-07	0.0002	0.011	+	None	None
ZNF765 (8)	rs2708790	4.90E-08	0.0002	0.011	-	None	None
PLEKHB2 (1)	rs78926001	8.30E-07	0.0002	0.011	+	Enhancer	None
FAM167A (5)	rs2409769	4.30E-08	0.0002	0.011	-	Enhancer	Enhancer
AC007246.3 (15)	rs13007944	2.20E-07	0.0002	0.011	+	None	None
MRPS10 (0)	rs6458276	2.50E-07	0.0002	0.011	+	Enhancer	Enhancer
PCGF2 (3)	rs1239185	8.10E-08	0.0002	0.011	+	None	None
HDAC2 (13)	rs578289	3.20E-07	0.0002	0.011	+	None	None
TMEM9B (6)	rs2742478	1.60E-07	0.0002	0.011	-	None	None
STXBP4 (10)	rs12162133	2.80E-07	0.0002	0.011	-	None	None
PPM1F (0)	rs3859841	4.90E-08	0.0002	0.011	-	None	None
TMCO3 (12)	rs9577274	4.70E-08	0.0002	0.011	-	Transcription	None
CTAGE15 (0)	chr7:144060802	3.40E-07	0.0002	0.011	+	None	None

SPATA6L (18)	rs9776157	1.20E-07	0.0002	0.011	-	Enhancer	Promoter
STARD10 (1)	rs7109575	4.00E-07	0.0002	0.011	+	Promoter	Promoter
SIDT1 (26)	rs35350834	9.70E-08	0.0002	0.011	-	Enhancer	Promoter
AC020571.3 (2)	rs17231419	1.70E-07	0.0002	0.011	+	None	Enhancer
ZDHHC3 (10)	rs4683026	3.30E-07	0.0002	0.011	-	None	None
AC009228.1 (2)	rs11125478	3.30E-07	0.0002	0.011	-	None	None
AGPAT4 (4)	rs9456748	1.30E-07	0.0002	0.011	-	None	None
SLC25A39 (3)	rs9890354	4.00E-07	0.0002	0.011	-	None	None
GSTCD (5)	rs4699153	2.40E-07	0.0002	0.011	+	None	None
GAS8 (0)	rs4627348	2.10E-08	0.0002	0.011	+	Transcription	Transcription
P2RX2 (2)	rs7137282	3.10E-07	0.0002	0.011	-	None	Repressed
STEAP2 (8)	rs150666279	2.70E-07	0.0002	0.011	-	None	None
VWA5B1 (13)	rs4655192	2.60E-07	0.0002	0.011	-	None	None
SLC6A2 (15)	rs168924	6.40E-08	0.0002	0.011	+	Promoter	Repressed
FAM65A (0)	rs2136649	4.80E-07	0.0002	0.011	+	Enhancer	Enhancer
NLRP2 (18)	rs1654505	8.80E-09	0.0002	0.011	+	Repressed	None
ZNF626 (0)	rs6511148	4.80E-07	0.0002	0.011	+	Transcription	Promoter
MGMT (7)	rs12241533	8.80E-08	0.0002	0.011	-	None	None
SNX11 (3)	rs7209585	4.50E-08	0.0002	0.011	+	None	None
BEST3 (8)	rs57897082	1.70E-07	0.0002	0.011	+	None	None
NCK2 (6)	rs6720776	1.30E-07	0.0002	0.011	-	None	None
HIRIP3 (4)	rs3953224	2.00E-07	0.0002	0.011	+	None	None
COL6A2 (26)	rs1044598	6.40E-07	0.0003	0.015	-	None	None
KLHL3 (21)	rs4835755	2.90E-07	0.0003	0.015	-	None	None
MICAL1 (7)	rs12206928	3.20E-07	0.0003	0.015	-	Transcription	Transcription
BAIAP3 (11)	rs113501236	3.50E-08	0.0003	0.015	-	Enhancer	Enhancer
VSIG10 (0)	rs7956285	1.40E-07	0.0003	0.015	+	Enhancer	Enhancer
KAT8 (3)	rs7196161	9.00E-07	0.0003	0.015	+	None	None
DTYMK (0)	rs113056176	7.00E-07	0.0003	0.015	-	Transcription	Transcription
RCN2 (4)	rs12591631	7.30E-07	0.0003	0.015	-	Repressed	Repressed
C8orf59 (1)	rs7835882	1.20E-06	0.0003	0.015	+	Transcription	None
DNMT1 (27)	rs754529	1.00E-07	0.0003	0.015	-	None	Enhancer
RUFY2 (0)	rs12217908	3.00E-07	0.0003	0.015	+	Promoter	Promoter
PDE4DIP (53)	rs72704234	2.60E-06	0.0003	0.015	-	None	None
PTPRQ (65)	rs75011874	2.00E-07	0.0003	0.015	-	None	None
LRRC14 (3)	rs2721161	5.10E-07	0.0003	0.015	+	None	None
MLC1 (5)	rs9616319	4.10E-10	0.0003	0.015	+	Repressed	Repressed
ARPC2 (6)	rs6436049	3.10E-07	0.0003	0.015	-	Transcription	Transcription
ANO3 (16)	rs9988921	4.40E-07	0.0003	0.015	+	None	None
EML3 (2)	rs12798970	6.60E-07	0.0003	0.015	-	Promoter	Promoter
ALDH9A1 (10)	rs12408101	6.90E-07	0.0003	0.015	-	Promoter	Promoter
GCNT3 (11)	rs1479885	3.80E-07	0.0003	0.015	-	None	Transcription
C5orf17 (4)	rs6889928	3.60E-08	0.0003	0.015	-	None	None
KRT34 (0)	rs73303160	3.10E-08	0.0003	0.015	+	None	None
ESPNL (9)	rs13382446	7.50E-08	0.0003	0.015	+	None	Repressed
CACTIN (0)	rs12460780	1.10E-07	0.0003	0.015	-	None	None
NDE1 (9)	rs59402996	1.40E-07	0.0003	0.015	+	None	None
HSPA4 (14)	rs72801474	2.20E-07	0.0003	0.015	-	None	None
C5orf54 (1)	rs4921136	2.80E-08	0.0003	0.015	+	Enhancer	Enhancer
WHSC1 (13)	rs61789437	2.40E-07	0.0003	0.015	-	None	None
TREH (6)	rs11605069	1.50E-07	0.0003	0.015	+	None	None
SNX15 (2)	rs7936265	4.40E-07	0.0003	0.015	+	Repressed	Repressed
DALRD3 (3)	rs62264271	2.30E-06	0.0003	0.015	-	None	None
SH3BP1 (9)	rs9610776	3.80E-07	0.0003	0.015	-	Enhancer	Enhancer
SMG7 (28)	rs2782411	2.50E-07	0.0003	0.015	-	None	None
RP11-1026M7.2 (0)	rs34015040	3.20E-07	0.0003	0.015	-	None	None
CCZ1B (7)	chr7:5949726	2.60E-07	0.0003	0.015	+	None	None
ZNF534 (4)	rs73586087	1.40E-07	0.0003	0.015	+	None	Promoter
ZNF391 (5)	rs9468082	9.60E-07	0.0003	0.015	+	None	None
MMAB (0)	rs7306218	7.40E-07	0.0003	0.015	-	Enhancer	None
UPK1A (7)	rs4806187	2.50E-07	0.0003	0.015	+	Transcription	Transcription
FAIM (2)	rs774015	1.20E-07	0.0003	0.015	-	None	Repressed
RP11-496N12.9 (0)	rs12032961	7.20E-07	0.0003	0.015	-	None	None
LARP7 (1)	rs74862084	3.90E-07	0.0003	0.015	+	None	None
ZNF496 (1)	rs10754544	1.20E-07	0.0003	0.015	+	Transcription	Transcription
TM7SF3 (12)	rs1166773	9.00E-08	0.0003	0.015	+	Transcription	Transcription
SPC25 (7)	rs2232328	7.70E-07	0.0003	0.015	-	Enhancer	Enhancer
RP11-1070N10.7 (0)	rs10151475	4.70E-10	0.0003	0.015	-	Repressed	Repressed
CHID1 (13)	rs4963207	1.80E-07	0.0003	0.015	-	None	None
TMEM9B-AS1 (3)	rs7482838	1.60E-07	0.0003	0.015	+	Repressed	None
N4BP2L2 (9)	rs61945235	3.90E-07	0.0003	0.015	+	None	None
TMED9 (2)	rs13164561	1.20E-07	0.0003	0.015	+	None	None
GPBP1L1 (0)	rs2991980	3.10E-07	0.0003	0.015	+	Promoter	Promoter
AC006116.17 (0)	rs62128198	1.80E-08	0.0003	0.015	-	None	None
SOX11 (0)	rs6732265	1.70E-07	0.0003	0.015	-	Repressed	Repressed
ILK (1)	rs2555174	2.00E-07	0.0003	0.015	-	Enhancer	Enhancer
CHD2 (38)	rs62045476	4.30E-08	0.0003	0.015	+	Enhancer	Enhancer
ADAM22 (35)	rs3753105	4.20E-07	0.0003	0.015	-	Enhancer	None
ZNHIT6 (0)	rs7520491	2.20E-07	0.0003	0.015	+	None	None
CCBL2 (6)	rs35594043	3.80E-07	0.0003	0.015	-	None	None
MAP3K4 (10)	rs2665356	4.40E-07	0.0003	0.015	+	None	None
TMEM50B (2)	rs73195833	2.00E-07	0.0003	0.015	-	None	Enhancer
TMEM179 (1)	rs7155652	1.80E-07	0.0003	0.015	-	None	None
MYSM1 (13)	rs183171777	1.60E-07	0.0003	0.015	-	None	None
GULP1 (13)	rs1439901	1.50E-06	0.0003	0.015	-	None	None
MTNR1B (0)	rs9804472	5.50E-07	0.0003	0.015	-	None	None
ZADH2 (0)	rs4891244	4.60E-07	0.0003	0.015	-	None	None
MMP24-AS1 (6)	rs2425043	6.10E-07	0.0003	0.015	-	Transcription	Enhancer
PIAS4 (5)	rs2656921	2.70E-07	0.0003	0.015	-	None	None
ZNF84 (1)	rs116809216	2.60E-07	0.0003	0.015	-	Enhancer	None
STMN2 (6)	rs1235214	8.60E-08	0.0003	0.015	-	None	None
MCM3AP (12)	rs3949169	3.80E-07	0.0003	0.015	-	Transcription	None
FTSJ2 (1)	rs3800938	2.30E-07	0.0003	0.015	+	Transcription	None
KANK2 (13)	rs7250652	1.80E-07	0.0003	0.015	-	Transcription	Transcription

TEX9 (5)	rs16976919	3.40E-07	0.0003	0.015	-	Insulator	None
TRAF3IP1 (4)	rs113401137	1.80E-07	0.0003	0.015	-	None	None
BPHL (6)	rs75014223	2.30E-07	0.0003	0.015	-	None	None
EPB41L2 (15)	rs76695086	4.50E-07	0.0003	0.015	-	None	None
RPAP1 (2)	rs8035331	5.30E-07	0.0003	0.015	-	None	Repressed
INTS8 (24)	rs2607059	1.70E-07	0.0003	0.015	-	None	None
WFIKKN1 (0)	rs62030917	4.30E-07	0.0003	0.015	+	Enhancer	Enhancer
ZCRB1 (8)	rs10785303	3.80E-07	0.0003	0.015	+	None	None
PKD1L3 (6)	rs212171	1.30E-07	0.0003	0.015	+	None	None
FAM183A (0)	rs11210820	1.30E-08	0.0003	0.015	-	None	None
GALT (2)	rs10758232	2.70E-07	0.0003	0.015	+	None	None
PCDHB16 (0)	rs17844664	1.80E-07	0.0003	0.015	-	Transcription	None
SPAG6 (11)	rs150906719	7.80E-07	0.0003	0.015	-	None	None
MED16 (5)	rs55639032	2.20E-07	0.0004	0.019	+	Transcription	Enhancer
BBS7 (14)	rs2390170	4.40E-07	0.0004	0.019	+	None	None
RPS9 (0)	rs34924761	1.70E-07	0.0004	0.019	+	Enhancer	Enhancer
RP11-105C19.2 (0)	rs7190339	1.60E-07	0.0004	0.019	-	Repressed	None
FAM185A (1)	rs494543	3.60E-07	0.0004	0.019	-	None	None
FBP2 (0)	rs479197	2.20E-07	0.0004	0.019	+	None	None
RP11-66D17.3 (0)	rs17412946	8.90E-07	0.0004	0.019	+	None	Repressed
RP11-230B22.1 (0)	rs76097236	4.40E-07	0.0004	0.019	-	Enhancer	Enhancer
CLCN7 (10)	rs11860968	2.90E-07	0.0004	0.019	-	Transcription	Repressed
RP11-568A7.2 (1)	rs1854859	3.70E-07	0.0004	0.019	-	None	None
GIT1 (20)	rs9914175	5.20E-07	0.0004	0.019	-	None	None
ZNF615 (0)	rs111736629	3.40E-07	0.0004	0.019	-	None	Promoter
MTRF1 (12)	rs61959252	1.80E-07	0.0004	0.019	-	None	None
SEMA4A (17)	rs76719272	1.20E-06	0.0004	0.019	+	None	None
IKZF5 (1)	rs4388815	3.70E-07	0.0004	0.019	+	None	None
LINC00116 (1)	rs61730258	1.00E-06	0.0004	0.019	-	None	None
TBL1XR1 (13)	rs6799682	2.80E-07	0.0004	0.019	+	None	None
AC074011.2 (0)	rs35244334	4.60E-07	0.0004	0.019	+	Enhancer	Enhancer
AC133633.1 (1)	rs35613955	1.80E-09	0.0004	0.019	+	None	Repressed
GLTSCR2 (2)	rs4561582	3.40E-07	0.0004	0.019	-	None	None
CEP63 (15)	rs6799543	2.70E-07	0.0004	0.019	+	Transcription	Transcription
TOP2A (18)	rs2071427	2.80E-07	0.0004	0.019	+	Enhancer	Enhancer
NGEF (12)	rs1083518	1.80E-07	0.0004	0.019	+	Repressed	Repressed
ELOVL5 (6)	rs9382197	4.70E-07	0.0004	0.019	+	None	None
RBPMS (20)	rs10099387	3.20E-08	0.0004	0.019	+	None	None
HCG18 (4)	rs116127115	3.60E-08	0.0004	0.019	+	Transcription	Transcription
COL4A1 (21)	rs12431029	1.90E-07	0.0004	0.019	+	Enhancer	None
AC104135.4 (0)	rs7589773	1.70E-07	0.0004	0.019	+	None	None
USP34 (26)	rs72805330	2.80E-07	0.0004	0.019	-	Repressed	None
ALAS1 (2)	rs2952830	1.40E-06	0.0004	0.019	-	None	None
CEP97 (9)	rs56759184	5.50E-07	0.0004	0.019	-	Enhancer	None
MYO5C (27)	rs2639676	3.00E-07	0.0004	0.019	-	None	None
CTC-308K20.1 (0)	rs28550282	3.00E-08	0.0004	0.019	-	None	Enhancer
ZNF333 (0)	rs3893727	3.10E-07	0.0004	0.019	-	Transcription	Transcription
ELMOD2 (3)	rs17006132	2.50E-07	0.0004	0.019	+	None	None
LINC00989 (3)	rs113291027	3.80E-07	0.0004	0.019	-	None	None
VWA3B (22)	rs72957179	6.30E-07	0.0004	0.019	+	None	None
IPO8 (26)	rs10843816	1.70E-07	0.0004	0.019	+	None	None
ZNF195 (8)	rs2412171	9.60E-08	0.0004	0.019	+	Enhancer	Enhancer
SUPT6H (2)	rs2286519	5.90E-07	0.0004	0.019	+	Transcription	Transcription
PHLDB3 (13)	rs2599457	1.70E-07	0.0004	0.019	+	None	None
RP11-526I2.5 (0)	rs12916563	4.20E-08	0.0004	0.019	-	None	None
MEF2B (0)	rs72997105	1.20E-06	0.0004	0.019	-	None	None
PLXNB2 (35)	rs5771266	5.80E-08	0.0004	0.019	+	Enhancer	Promoter
SLC9B2 (1)	rs659812	6.30E-07	0.0004	0.019	+	None	None
HEBP1 (3)	rs735689	4.80E-08	0.0004	0.019	+	Insulator	Insulator
TPCN2 (19)	rs10792030	7.60E-07	0.0004	0.019	+	Promoter	Repressed
KIAA1211 (14)	rs73240517	2.00E-07	0.0004	0.019	+	None	Enhancer
C19orf24 (2)	rs2379240	1.10E-07	0.0004	0.019	+	Repressed	Repressed
SMG5 (6)	rs2151132	4.50E-07	0.0004	0.019	+	None	None
ABCA7 (41)	rs1137776	1.10E-07	0.0004	0.019	-	Transcription	None
AQP3 (1)	rs72707502	5.00E-07	0.0004	0.019	+	Enhancer	Enhancer
RP11-531A24.5 (0)	rs11782712	4.80E-10	0.0004	0.019	-	None	Transcription
TBC1D1 (18)	rs6531601	4.90E-07	0.0004	0.019	-	Enhancer	Enhancer
SNRNP27 (2)	rs4853017	4.30E-07	0.0004	0.019	+	None	Enhancer
TIMM9 (4)	rs59742322	5.20E-07	0.0004	0.019	+	None	None
NT5C (2)	rs113565684	8.40E-07	0.0004	0.019	-	None	None
ANXA6 (29)	rs2303038	1.20E-07	0.0004	0.019	-	Enhancer	Enhancer
SNX2 (9)	rs763497	8.30E-07	0.0004	0.019	-	None	None
YME1L1 (11)	rs11015598	2.60E-07	0.0004	0.019	-	Enhancer	Enhancer
RP11-98D18.9 (1)	rs146830974	2.70E-07	0.0004	0.019	+	None	None
S100B (0)	rs7282606	3.00E-07	0.0004	0.019	+	None	None
DGKB (22)	rs10278686	2.80E-07	0.0004	0.019	-	None	None
MROH8 (23)	rs76371791	1.80E-06	0.0004	0.019	-	Transcription	Enhancer
RP11-1191J2.4 (0)	rs35214302	6.10E-07	0.0004	0.019	+	Repressed	None
RP11-645C24.5 (0)	rs71374899	2.10E-07	0.0004	0.019	-	None	None
RP11-360F5.1 (1)	rs34314908	3.20E-07	0.0004	0.019	-	None	None
RP11-507K2.2 (4)	rs8017299	7.10E-07	0.0004	0.019	+	Transcription	None
EPDR1 (3)	rs10260487	4.20E-07	0.0005	0.023	+	None	None
LINC00623 (17)	rs61807905	1.50E-05	0.0005	0.023	+	Transcription	Transcription
EP300 (6)	rs8135045	4.00E-07	0.0005	0.023	-	None	None
XAF1 (8)	rs178340	2.00E-07	0.0005	0.023	+	None	None
RP11-434C1.1 (3)	rs2418220	4.30E-07	0.0005	0.023	-	Transcription	None
RAD51-AS1 (2)	rs2619682	7.70E-07	0.0005	0.023	+	None	None
PCDP1 (17)	rs2244213	9.10E-07	0.0005	0.023	-	Promoter	Promoter
AACS (21)	rs34961756	3.70E-07	0.0005	0.023	+	Enhancer	Enhancer
NEU4 (7)	rs1609990	2.10E-07	0.0005	0.023	+	None	Repressed
TRIM66 (0)	rs10840114	3.20E-07	0.0005	0.023	+	Transcription	Transcription
MYOZ3 (5)	rs11744028	3.50E-07	0.0005	0.023	-	Repressed	None
COL7A1 (64)	rs6773254	1.90E-06	0.0005	0.023	-	None	None
DNAJB6 (11)	rs56160067	2.60E-07	0.0005	0.023	+	Enhancer	Transcription

HIBADH (0)	rs12670250	7.90E-07	0.0005	0.023	+	None	None
RP11-1C8.4 (5)	rs7002944	7.60E-07	0.0005	0.023	-	None	None
EFCAB13 (14)	rs9894188	6.40E-07	0.0005	0.023	+	None	Enhancer
RP11-756H6.1 (1)	rs60615836	7.00E-07	0.0005	0.023	+	None	None
EHHADH (0)	rs73066840	4.10E-07	0.0005	0.023	+	None	None
DMXL2 (23)	rs16964254	3.70E-07	0.0005	0.023	-	Enhancer	None
ARHGAP19 (1)	rs12777701	5.50E-08	0.0005	0.023	+	Transcription	None
HLTF (24)	rs3732559	4.10E-07	0.0005	0.023	-	None	None
RP11-475N22.4 (1)	rs2659699	7.30E-07	0.0005	0.023	+	Repressed	Repressed
ZNF652 (1)	rs112163423	5.70E-07	0.0005	0.023	+	None	None
PGLS (1)	rs4808084	2.40E-07	0.0005	0.023	-	Enhancer	Enhancer
CECR7 (4)	rs2537972	2.10E-07	0.0005	0.023	-	None	Repressed
NOS2 (3)	rs111499547	5.10E-07	0.0005	0.023	-	None	None
CRY1 (12)	rs12298001	1.00E-06	0.0005	0.023	-	None	None
RP11-569G13.2 (5)	rs4559354	6.10E-07	0.0005	0.023	+	None	None
POMGNT1 (1)	rs28364741	8.10E-07	0.0005	0.023	-	Promoter	Promoter
DISP2 (4)	rs35565646	6.80E-07	0.0005	0.023	+	Insulator	Insulator
SLC35F4 (4)	rs9671923	1.90E-07	0.0005	0.023	+	Enhancer	None
CDADC1 (5)	rs73186813	6.00E-07	0.0005	0.023	-	None	Enhancer
COTL1 (0)	rs2967868	1.00E-07	0.0005	0.023	+	Enhancer	Promoter
CCDC159 (1)	rs111737410	3.60E-07	0.0005	0.023	+	None	None
NUB1 (10)	rs406624	3.90E-07	0.0005	0.023	+	Transcription	Transcription
TMEM253 (5)	rs4470055	2.70E-07	0.0005	0.023	+	Enhancer	Enhancer
CERCAM (9)	rs7849975	1.10E-06	0.0005	0.023	+	Promoter	Promoter
LRRFIP2 (29)	rs4678561	5.80E-07	0.0005	0.023	-	None	None
ITSN1 (6)	rs4817512	5.10E-07	0.0005	0.023	+	None	Repressed
DCXR (2)	rs57552134	4.50E-07	0.0005	0.023	+	Promoter	Promoter
TAGLN3 (4)	rs9839391	9.20E-08	0.0005	0.023	+	Enhancer	Enhancer
CCDC50 (3)	rs696351	5.00E-07	0.0005	0.023	+	None	None
CTSB (8)	rs6990033	3.80E-07	0.0005	0.023	-	Transcription	Transcription
MRPS18C (0)	rs76236608	5.20E-07	0.0005	0.023	-	None	None
GPN1 (2)	rs71443025	8.50E-07	0.0005	0.023	+	None	None
RACGAP1 (2)	rs7486874	2.40E-06	0.0005	0.023	+	None	None
PLEKHA1 (8)	rs71486610	3.40E-07	0.0005	0.023	-	Promoter	Promoter
PIP5K1A (11)	rs10888406	1.20E-06	0.0005	0.023	+	None	None
RP11-95D17.1 (0)	rs394650	2.60E-07	0.0005	0.023	-	None	None
ZNF737 (0)	rs1582997	2.10E-07	0.0005	0.023	+	None	None
CAMKK1 (15)	rs12937934	1.30E-07	0.0005	0.023	+	None	None
GRIN3A (3)	rs7873495	6.90E-10	0.0005	0.023	+	Enhancer	None
NEU3 (2)	rs12272818	7.10E-07	0.0005	0.023	-	None	None
MSH2 (6)	rs11692854	3.40E-07	0.0005	0.023	-	None	Transcription
BCO2 (3)	rs12419156	8.50E-07	0.0005	0.023	-	None	None
SHISA6 (5)	rs62062059	2.20E-07	0.0005	0.023	+	None	None
AC138969.4 (13)	rs9933569	4.60E-09	0.0005	0.023	-	None	None
LINC00863 (0)	rs41324346	2.40E-07	0.0005	0.023	+	None	None
TSGA10 (2)	rs72957109	1.60E-07	0.0005	0.023	-	None	None
PFKL (10)	rs909425	2.30E-07	0.0005	0.023	-	None	Repressed
RIMBP2 (18)	rs7976450	1.40E-07	0.0005	0.023	-	Enhancer	Enhancer
RBL2 (6)	rs9930106	9.50E-07	0.0006	0.027	-	None	None
LGALS9B (0)	rs75417978	4.10E-07	0.0006	0.027	+	None	None
ESRP1 (15)	rs114055430	4.40E-07	0.0006	0.027	-	None	None
ARID5A (7)	rs772174	1.20E-06	0.0006	0.027	+	Promoter	Promoter
TNNT2 (14)	rs868407	3.00E-07	0.0006	0.027	-	Repressed	Repressed
ADH5 (0)	rs62323245	5.80E-07	0.0006	0.027	-	None	None
UNC93B1 (3)	rs3094930	9.70E-07	0.0006	0.027	+	None	None
RP11-10K16.1 (3)	rs934358	3.80E-07	0.0006	0.027	+	None	None
ZNF208 (3)	rs4932746	1.10E-06	0.0006	0.027	-	Transcription	Promoter
METTL21A (2)	rs2551949	3.90E-07	0.0006	0.027	+	None	None
GPR114 (6)	rs3931940	1.80E-07	0.0006	0.027	-	Enhancer	Enhancer
CHN2 (6)	rs245904	1.60E-07	0.0006	0.027	+	Repressed	None
CTD-2530H12.4 (0)	rs584046	8.30E-07	0.0006	0.027	-	None	Enhancer
CIRH1A (17)	rs113827003	4.50E-07	0.0006	0.027	-	None	Enhancer
RP5-1018K9.1 (1)	rs4614315	8.30E-07	0.0006	0.027	+	None	None
C8B (4)	rs10888960	7.00E-07	0.0006	0.027	+	None	Insulator
TLK2 (23)	rs2032737	1.20E-06	0.0006	0.027	-	Insulator	Insulator
MARK3 (10)	rs10132641	7.80E-07	0.0006	0.027	-	Promoter	Promoter
RP11-173P15.3 (0)	rs73222795	4.50E-07	0.0006	0.027	-	None	None
RP11-798G7.8 (0)	rs141293023	4.30E-07	0.0006	0.027	-	None	None
SRPK2 (21)	rs6962220	5.10E-07	0.0006	0.027	+	None	None
SLC25A51 (0)	rs55889614	3.80E-07	0.0006	0.027	+	Transcription	Transcription
HR (5)	rs34034897	1.90E-07	0.0006	0.027	-	None	None
RP11-535A19.2 (3)	rs139804261	1.70E-07	0.0006	0.027	+	Enhancer	None
RP11-529H2.2 (2)	rs7685308	5.10E-07	0.0006	0.027	+	Transcription	Transcription
MAPK13 (7)	rs12201042	9.70E-07	0.0006	0.027	-	None	None
SNRPN (7)	rs4906936	2.90E-07	0.0006	0.027	-	None	None
PPP2R1A (0)	rs113671789	3.80E-07	0.0006	0.027	-	Promoter	Promoter
PDZD7 (0)	rs12259511	1.10E-06	0.0006	0.027	+	Repressed	Repressed
ACAA2 (0)	rs665194	4.20E-07	0.0006	0.027	-	Transcription	Transcription
GNPAT (0)	rs16852515	3.20E-07	0.0006	0.027	-	None	None
LSS (16)	rs35679325	2.10E-07	0.0006	0.027	+	Transcription	Transcription
MAEA (10)	rs4333131	3.40E-07	0.0006	0.027	-	None	None
MMS19 (28)	rs12569570	4.20E-07	0.0006	0.027	+	Transcription	Transcription
DDTL (0)	rs4820571	5.20E-07	0.0006	0.027	-	None	None
PSMD2 (21)	rs181896693	7.00E-07	0.0006	0.027	+	Repressed	Repressed
TVP23C (2)	rs58393633	4.90E-07	0.0006	0.027	-	None	None
SLC35F5 (0)	rs6709158	3.50E-07	0.0006	0.027	+	Transcription	Transcription
SCN2B (0)	rs629397	4.00E-07	0.0006	0.027	+	None	None
GCSAML-AS1 (0)	rs10802510	7.50E-07	0.0006	0.027	+	None	None
PXDC1 (4)	rs72650640	1.60E-07	0.0006	0.027	-	None	None
ABHD3 (1)	rs7407412	1.30E-06	0.0006	0.027	+	None	None
SGK223 (3)	rs2945874	6.50E-07	0.0006	0.027	-	None	None
LYRM4 (0)	rs111557465	5.40E-07	0.0006	0.027	+	Promoter	Promoter
DMPK (6)	rs10401487	5.20E-07	0.0006	0.027	+	None	None
ATP5G3 (3)	rs10183474	3.80E-07	0.0006	0.027	+	None	None

LINC00617 (3)	rs11623080	1.10E-07	0.0006	0.027	-	Repressed	Repressed
METTL21B (1)	rs10431505	8.80E-07	0.0006	0.027	+	Enhancer	Transcription
ATP6V1E2 (4)	rs10175812	7.10E-08	0.0006	0.027	-	None	None
FUT9 (4)	rs6915529	2.70E-07	0.0006	0.027	+	None	None
C7orf73 (2)	rs3110823	1.00E-06	0.0006	0.027	-	Insulator	Enhancer
MARCH6 (25)	rs2640709	1.50E-07	0.0006	0.027	+	None	Enhancer
APOBEC3G (8)	rs6519166	5.90E-07	0.0006	0.027	-	None	None
PIGQ (12)	rs4262946	1.40E-07	0.0006	0.027	-	None	None
RP11-110I1.14 (0)	rs3759012	3.30E-07	0.0006	0.027	+	Promoter	Promoter
PRKCI (17)	rs73172097	1.00E-06	0.0006	0.027	-	None	None
RP11-105C19.1 (0)	rs72782890	1.70E-07	0.0006	0.027	-	None	None
RP11-140H17.1 (0)	rs112407786	7.30E-07	0.0006	0.027	+	Transcription	None
PROCR (2)	rs6060288	2.00E-06	0.0006	0.027	+	None	None
UGGT2 (14)	rs7984912	2.60E-07	0.0006	0.027	-	None	Enhancer
RP11-488P3.1 (0)	rs4847255	5.80E-07	0.0006	0.027	+	None	Enhancer
DGKH (21)	rs2780650	1.00E-06	0.0006	0.027	-	None	None
C10orf90 (7)	rs11245088	3.90E-07	0.0006	0.027	-	None	None
EIF2A (14)	rs6440685	3.50E-07	0.0006	0.027	+	Enhancer	Enhancer
RP11-680H20.2 (2)	rs1792632	3.70E-07	0.0006	0.027	+	None	None
ADAM15 (17)	rs11589479	5.40E-07	0.0006	0.027	-	None	None
NXF1 (8)	rs2070870	6.60E-07	0.0006	0.027	-	Promoter	Promoter
GPR98 (59)	rs74402761	6.70E-07	0.0007	0.03	+	None	None
SRP68 (11)	rs111598470	7.20E-07	0.0007	0.03	-	Transcription	Transcription
CHD1 (0)	rs151102	9.90E-07	0.0007	0.03	-	None	None
CCNY (8)	rs16936653	1.30E-08	0.0007	0.03	+	None	None
FAM66A (2)	rs1478898	1.60E-07	0.0007	0.03	+	Enhancer	None
GS1-124K5.11 (1)	rs17711722	2.20E-06	0.0007	0.03	+	None	None
PITPNC1 (10)	rs12952954	7.20E-07	0.0007	0.03	-	Transcription	Transcription
EIF4G1 (24)	rs6786200	7.20E-07	0.0007	0.03	-	None	None
GLB1L2 (21)	rs11608146	5.60E-07	0.0007	0.03	+	None	None
UBA6 (25)	rs141915916	1.10E-06	0.0007	0.03	-	None	None
HRAS (4)	rs7944548	3.50E-07	0.0007	0.03	-	Transcription	Enhancer
GLS2 (6)	rs2657910	6.60E-07	0.0007	0.03	-	None	None
MAPKBP1 (27)	rs78325423	4.10E-07	0.0007	0.03	-	None	None
RP4-553F4.6 (2)	rs2050211	1.80E-06	0.0007	0.03	-	Repressed	None
RP11-499O7.7 (0)	rs61830354	4.80E-07	0.0007	0.03	-	None	None
RP11-44F14.8 (0)	rs2908786	1.60E-07	0.0007	0.03	-	None	Repressed
RBCK1 (11)	rs6107514	2.30E-07	0.0007	0.03	+	Transcription	Enhancer
CADPS2 (1)	rs4731059	1.00E-06	0.0007	0.03	-	None	None
C8orf49 (0)	rs185051096	5.10E-07	0.0007	0.03	-	None	None
IL17RC (0)	rs77048068	2.20E-07	0.0007	0.03	-	Repressed	Repressed
SNAP91 (10)	rs9791269	1.70E-06	0.0007	0.03	+	None	Repressed
C1QTNF9B (0)	rs56024868	1.20E-07	0.0007	0.03	-	None	None
LINC00242 (0)	rs10455751	2.80E-09	0.0007	0.03	+	None	Repressed
EPT1 (3)	rs116693164	5.30E-07	0.0007	0.03	-	Enhancer	Enhancer
GORAB (6)	rs12116863	1.30E-06	0.0007	0.03	+	None	None
CLTCL1 (19)	rs1206535	9.70E-07	0.0007	0.03	+	None	None
ST18 (20)	rs148223527	7.70E-07	0.0007	0.03	+	Transcription	None
RNF166 (5)	rs2306048	6.70E-07	0.0007	0.03	-	None	Enhancer
TMEM167B (3)	rs4970818	7.90E-07	0.0007	0.03	+	None	None
TUBG1 (7)	rs12949175	9.80E-07	0.0007	0.03	-	Enhancer	None
ACTB (9)	rs852500	1.50E-07	0.0007	0.03	+	None	None
CLIC6 (5)	rs13049745	5.20E-07	0.0007	0.03	+	Promoter	Promoter
TRAPPC4 (1)	rs8192696	4.30E-07	0.0007	0.03	+	Enhancer	Enhancer
FRG1 (0)	rs9917993	4.70E-07	0.0007	0.03	-	None	None
ATP5S (3)	rs1967919	2.60E-07	0.0007	0.03	-	Enhancer	Enhancer
SLC30A9 (3)	rs35471602	9.70E-07	0.0007	0.03	-	None	None
PLIN1 (0)	rs72762354	2.90E-07	0.0007	0.03	-	None	None
RP11-158I13.2 (10)	rs12468596	1.90E-06	0.0007	0.03	+	None	None
SPIDR (25)	rs142983042	1.30E-06	0.0007	0.03	-	None	None
RP11-1000B6.3 (3)	rs11632524	5.70E-07	0.0007	0.03	-	None	None
TNFRSF14 (6)	rs2234161	5.60E-07	0.0007	0.03	+	None	None
CEBPZ (10)	rs3213745	9.40E-07	0.0007	0.03	-	Promoter	Promoter
DNMT3B (9)	rs2424896	4.90E-07	0.0007	0.03	+	None	None
CLU (8)	rs56290800	5.90E-07	0.0007	0.03	-	Transcription	None
CLPTM1L (1)	rs115218804	1.50E-07	0.0007	0.03	-	Repressed	Repressed
ABCD4 (18)	rs55834600	9.00E-07	0.0007	0.03	-	Repressed	Repressed
AASDH (1)	rs13135046	9.20E-07	0.0007	0.03	-	None	None
ITIH5 (8)	rs17141695	3.80E-07	0.0007	0.03	-	None	None
ACTR1B (4)	rs62157588	1.30E-06	0.0007	0.03	+	Enhancer	None
NDUFC1 (7)	rs11944434	4.30E-07	0.0007	0.03	-	Transcription	Transcription
RP11-472M19.2 (2)	rs11970648	1.30E-06	0.0007	0.03	+	None	None
OR2A7 (0)	chr7:144060802	7.50E-07	0.0007	0.03	+	None	None
THSD7B (9)	rs77055332	1.80E-08	0.0007	0.03	+	Promoter	Promoter
HNRNPF (1)	rs1416226	1.50E-06	0.0007	0.03	+	Promoter	Promoter
RP11-234O6.2 (1)	rs79690961	1.40E-07	0.0007	0.03	+	None	None
ADARB2 (7)	rs4540773	4.90E-07	0.0007	0.03	+	None	None
EBAG9 (7)	rs2844232	4.70E-07	0.0007	0.03	+	Transcription	Transcription
RP11-502I4.3 (0)	rs57075395	9.30E-07	0.0007	0.03	+	None	None
NCMAP (1)	rs9728914	1.30E-06	0.0007	0.03	+	None	None
HOPX (1)	rs36076528	7.50E-07	0.0007	0.03	+	None	None
CDC25B (11)	rs910654	3.70E-07	0.0007	0.03	+	Promoter	Promoter
RP11-640M9.1 (11)	rs112298300	5.00E-06	0.0007	0.03	-	Enhancer	Enhancer
CXCL12 (5)	rs145409428	5.50E-07	0.0007	0.03	+	None	None
CTD-3126B10.4 (1)	rs11645136	6.00E-07	0.0008	0.034	-	Repressed	None
EPHX2 (6)	rs6558003	3.90E-07	0.0008	0.034	+	Transcription	Transcription
SCAMP2 (2)	rs12904227	1.10E-06	0.0008	0.034	-	None	None
OIP5-AS1 (9)	rs11635991	2.70E-07	0.0008	0.034	-	None	Transcription
RP11-585F1.10 (1)	rs10097920	3.50E-07	0.0008	0.034	-	None	None
CFDP1 (0)	rs37596	8.70E-07	0.0008	0.034	+	Promoter	Promoter
POLE4 (3)	rs6754413	1.10E-07	0.0008	0.034	-	None	None
AMER2 (0)	rs9553542	6.40E-07	0.0008	0.034	+	None	None
KRT6A (7)	rs599466	1.10E-06	0.0008	0.034	+	None	None
MYO1D (16)	rs3115687	7.80E-07	0.0008	0.034	-	None	None

RP11-256L6.2 (1)	rs11108372	5.10E-07	0.0008	0.034	+	None	Transcription
ABCA13 (61)	rs34196162	2.10E-07	0.0008	0.034	+	None	None
PIGK (10)	rs11162298	5.50E-07	0.0008	0.034	-	None	None
OSR2 (0)	rs2512044	1.30E-06	0.0008	0.034	+	None	None
CTDP1 (8)	rs34031764	5.30E-07	0.0008	0.034	-	Transcription	Transcription
GPR97 (11)	rs10852554	8.50E-07	0.0008	0.034	+	Transcription	Transcription
C21orf67 (0)	rs7278847	4.00E-07	0.0008	0.034	-	Repressed	Repressed
UBE4B (9)	rs7354817	5.20E-07	0.0008	0.034	-	Transcription	None
RPL29 (0)	rs13068631	3.00E-06	0.0008	0.034	-	Insulator	Enhancer
GPR56 (18)	rs935746	1.90E-07	0.0008	0.034	+	Enhancer	Enhancer
PISD (7)	rs11703808	3.60E-07	0.0008	0.034	-	Enhancer	Enhancer
ACTR10 (8)	rs78570253	1.20E-06	0.0008	0.034	+	None	None
HAX1 (6)	rs79269068	2.40E-06	0.0008	0.034	+	None	None
RP11-233G1.4 (1)	rs2445387	4.60E-08	0.0008	0.034	-	None	None
KM-PA-2 (3)	rs7388446	5.50E-07	0.0008	0.034	+	None	None
RP11-10A14.4 (4)	rs73195802	2.10E-08	0.0008	0.034	+	None	Transcription
DNTTIP1 (5)	rs150340993	1.00E-06	0.0008	0.034	+	None	None
RP11-137H2.4 (1)	rs11185932	4.40E-07	0.0008	0.034	-	Promoter	Promoter
ARHGEF37 (12)	rs965876	6.00E-07	0.0008	0.034	+	Transcription	Transcription
ARHGEF1 (32)	rs7256255	1.10E-06	0.0008	0.034	+	None	None
ZNF253 (3)	rs2160156	8.90E-07	0.0008	0.034	-	Transcription	None
ANAPC7 (5)	rs12322130	9.40E-07	0.0008	0.034	+	None	None
AP002954.4 (0)	rs3181261	8.30E-07	0.0008	0.034	+	Repressed	Repressed
FAM222B (7)	rs12938360	1.80E-06	0.0008	0.034	-	None	None
PHTF1 (14)	rs1230675	3.50E-07	0.0008	0.034	+	None	None
FAM131A (3)	rs7652854	1.80E-07	0.0008	0.034	-	None	Transcription
SMAP1 (8)	rs12210123	1.80E-06	0.0008	0.034	+	Repressed	None
MRPL1 (10)	rs10518195	2.10E-06	0.0008	0.034	+	None	None
IRAK2 (10)	rs4383496	6.20E-07	0.0008	0.034	-	Repressed	Repressed
ST3GAL4 (12)	rs12293992	4.50E-07	0.0008	0.034	+	None	None
CHD6 (28)	rs78046442	8.40E-07	0.0008	0.034	-	Enhancer	Enhancer
ANKRD36C (37)	rs4613328	2.40E-06	0.0008	0.034	+	None	None
RP11-64C12.4 (0)	rs7237465	4.20E-07	0.0008	0.034	-	None	None
THAP10 (0)	rs72753503	7.70E-07	0.0008	0.034	+	None	None
RERGL (2)	rs11044269	1.00E-06	0.0008	0.034	-	None	Insulator
CCDC85A (1)	rs1319240	7.00E-07	0.0008	0.034	-	Enhancer	None
IL6ST (7)	rs7731626	3.80E-07	0.0008	0.034	-	Enhancer	Enhancer
HIST1H2BK (0)	rs181459139	1.10E-06	0.0008	0.034	+	None	None
ELMO1 (16)	rs71535802	8.70E-07	0.0008	0.034	-	None	None
KIAA0586 (36)	rs989971	7.40E-07	0.0008	0.034	+	None	None
CHFR (26)	rs11147064	1.10E-06	0.0008	0.034	-	Enhancer	Promoter
PEA15 (0)	rs9633341	4.90E-07	0.0008	0.034	-	Transcription	Transcription
AC068039.4 (0)	rs4668407	9.00E-07	0.0008	0.034	-	None	None
CCDC144NL (7)	rs7218489	1.70E-06	0.0009	0.037	+	Enhancer	Repressed
SERPINC1 (4)	rs12077782	3.30E-06	0.0009	0.037	+	None	None
GTF3C1 (10)	rs1515886	5.90E-07	0.0009	0.037	+	Repressed	Repressed
CYB5A (3)	rs6566800	6.10E-08	0.0009	0.037	-	None	Repressed
VAV3 (23)	rs6583048	2.40E-07	0.0009	0.037	+	Transcription	None
H6PD (3)	rs7526864	6.70E-07	0.0009	0.037	-	None	Enhancer
TRIM37 (2)	rs4793954	1.80E-06	0.0009	0.037	-	None	None
FMN1 (17)	rs922511	5.80E-07	0.0009	0.037	-	None	Enhancer
NFYB (7)	rs17583022	2.00E-07	0.0009	0.037	-	None	None
RP4-647C14.2 (0)	rs177405	6.30E-07	0.0009	0.037	-	None	None
CSTF3 (12)	rs7927262	1.00E-06	0.0009	0.037	-	None	None
WRAP73 (0)	rs10752739	4.20E-07	0.0009	0.037	+	Repressed	None
ZSCAN23 (1)	rs74556591	2.10E-06	0.0009	0.037	+	None	Repressed
ZMYND11 (2)	rs2478247	1.10E-06	0.0009	0.037	-	None	None
RP5-1092A3.4 (0)	rs111715719	9.00E-07	0.0009	0.037	-	None	None
TBC1D31 (21)	rs2272723	7.80E-08	0.0009	0.037	-	None	None
GSTZ1 (9)	rs59630055	6.10E-07	0.0009	0.037	+	Enhancer	Enhancer
PLCG1 (0)	rs79305334	8.10E-07	0.0009	0.037	+	Enhancer	Enhancer
ZNF37A (0)	rs2505200	1.20E-06	0.0009	0.037	-	None	None
SNAPC4 (8)	rs11145930	2.10E-07	0.0009	0.037	-	Enhancer	Promoter
PTPLAD2 (6)	rs13284328	1.10E-06	0.0009	0.037	+	Enhancer	None
FLT3 (7)	rs6491257	7.40E-07	0.0009	0.037	-	None	Repressed
ZNF250 (3)	rs2975253	2.30E-06	0.0009	0.037	-	None	None
POLDIP2 (5)	rs71368117	3.00E-06	0.0009	0.037	-	Enhancer	None
CDHR1 (16)	rs73315862	9.60E-07	0.0009	0.037	+	None	Enhancer
GRM8 (10)	rs13227115	1.40E-06	0.0009	0.037	+	Enhancer	None
PRMT7 (13)	rs61593058	3.80E-06	0.0009	0.037	+	Transcription	Transcription
LMBRD2 (0)	rs11954023	4.10E-07	0.0009	0.037	-	None	None
GABRA5 (11)	rs76680719	9.20E-08	0.0009	0.037	+	None	None
CACNA2D1 (39)	rs28648983	8.70E-07	0.0009	0.037	-	None	None
MIOS (1)	rs73674582	6.50E-07	0.0009	0.037	-	None	Promoter
KIRREL3 (17)	rs35156054	4.60E-07	0.0009	0.037	-	None	None
BRWD1-IT2 (0)	rs12483559	3.20E-07	0.0009	0.037	-	Transcription	None
MRC1L1 (30)	rs691068	2.00E-07	0.0009	0.037	+	None	None
ADCY5 (1)	rs2717229	5.70E-07	0.0009	0.037	-	Transcription	Transcription
C22orf43 (5)	rs6003823	1.30E-06	0.0009	0.037	-	None	None
SNRNP200 (31)	rs1669762	2.20E-06	0.0009	0.037	-	Repressed	None
FAM229B (2)	rs1631968	1.10E-06	0.0009	0.037	+	Transcription	Transcription
COL12A1 (16)	rs45606834	6.10E-07	0.0009	0.037	-	None	Transcription
OTUB1 (3)	rs142540988	2.00E-06	0.0009	0.037	+	None	None
SLC4A3 (3)	rs60887428	1.00E-07	0.0009	0.037	-	None	None
DMAP1 (0)	rs226072	1.40E-06	0.0009	0.037	+	None	None
LZTFL1 (12)	rs9311379	1.40E-06	0.0009	0.037	-	Transcription	Transcription
IGF2BP2 (12)	rs12636310	4.30E-07	0.0009	0.037	-	None	None
ABCC8 (34)	rs36096028	6.20E-07	0.0009	0.037	-	Repressed	Repressed
OBSCN (7)	rs41303079	1.30E-06	0.0009	0.037	+	None	None
CLSTN2 (9)	rs16850488	6.00E-07	0.0009	0.037	-	None	None
ABCC1 (13)	rs4781708	3.90E-07	0.0009	0.037	-	Enhancer	None
ASRGL1 (1)	rs7924998	9.90E-07	0.0009	0.037	+	None	None
SORT1 (17)	rs570349	1.30E-06	0.0009	0.037	+	Enhancer	None
CNTN5 (18)	rs10892755	7.60E-07	0.0009	0.037	-	Enhancer	Enhancer

P4HTM (6)	rs62264271	5.10E-06	0.0009	0.037	-	None	None
CTB-43E15.2 (1)	rs1035878	5.60E-07	0.0009	0.037	+	Enhancer	Enhancer
CSNK1G2 (4)	rs8105415	1.70E-08	0.001	0.04	+	None	None
PKNOX2 (3)	rs11219981	9.40E-08	0.001	0.04	+	Repressed	None
INPP5B (15)	rs56217551	2.90E-07	0.001	0.04	+	Transcription	Transcription
LCN12 (3)	rs4242708	3.20E-07	0.001	0.04	-	None	None
MMP16 (5)	rs1603793	1.00E-06	0.001	0.04	+	None	Enhancer
HCG9 (0)	rs114770144	2.40E-07	0.001	0.04	-	Enhancer	Enhancer
MYO16 (4)	rs79407211	2.20E-08	0.001	0.04	+	None	None
RP11-418J17.1 (7)	rs7542832	1.40E-06	0.001	0.04	-	None	None
NME6 (0)	rs13068288	4.00E-06	0.001	0.04	-	Transcription	None
HSPA9 (11)	rs28363511	1.20E-06	0.001	0.04	-	Transcription	Enhancer
AC005152.2 (18)	rs2435979	1.70E-07	0.001	0.04	+	Enhancer	Enhancer
NKX6-3 (0)	rs12549902	6.10E-07	0.001	0.04	-	Promoter	None
CHRDL2 (0)	rs61900641	1.20E-06	0.001	0.04	-	None	Enhancer
SENP6 (0)	rs6910395	2.20E-06	0.001	0.04	+	None	None
TCOF1 (18)	rs148432105	4.30E-07	0.001	0.04	-	Transcription	Transcription
AC007787.2 (0)	rs111908806	1.50E-06	0.001	0.04	+	None	None
ZNF586 (3)	rs61497125	1.20E-06	0.001	0.04	+	None	None
SYT9 (5)	rs7934484	7.40E-07	0.001	0.04	+	Repressed	None
NFIB (9)	rs9696439	4.60E-07	0.001	0.04	+	None	None
FAM189A1 (2)	rs2636065	9.10E-07	0.001	0.04	-	None	None
RP11-24J23.2 (4)	rs7533812	9.70E-07	0.001	0.04	-	None	None
ILVBL (14)	rs113170451	7.30E-07	0.001	0.04	+	Transcription	Transcription
H1FX (0)	rs2811429	1.80E-06	0.001	0.04	+	Enhancer	Enhancer
TRIM52 (2)	rs2545086	4.90E-07	0.001	0.04	-	None	None
EML5 (23)	rs2145710	1.10E-06	0.001	0.04	+	None	None
PNPLA2 (9)	rs12277141	5.20E-07	0.001	0.04	-	Enhancer	None
RP11-273G15.2 (3)	rs12681773	7.70E-08	0.001	0.04	-	None	None
CHD7 (13)	rs1905305	2.40E-06	0.001	0.04	-	Enhancer	None
FAM153C (22)	rs7716970	2.80E-07	0.001	0.04	+	None	None
FAM107A (4)	rs73089526	6.70E-07	0.001	0.04	-	None	None
H2AFY2 (3)	rs73268556	4.60E-07	0.001	0.04	-	None	None
AC013460.1 (9)	rs56026054	2.80E-07	0.001	0.04	+	Enhancer	Enhancer
RP11-12C17.2 (1)	rs2433673	1.10E-07	0.001	0.04	-	None	None
CLDN10-AS1 (2)	rs7982853	8.20E-07	0.001	0.04	+	Enhancer	Enhancer
FAM66C (7)	rs7306155	4.00E-07	0.001	0.04	-	None	None
WASL (10)	rs7778957	1.10E-06	0.001	0.04	+	None	None
DTNA (27)	rs11660071	1.30E-06	0.001	0.04	+	None	None
C3orf52 (8)	rs6764824	1.50E-06	0.001	0.04	-	None	None
AL773572.7 (2)	rs2839411	3.20E-07	0.001	0.04	+	Enhancer	None
EPS8L1 (21)	rs111231617	3.60E-07	0.001	0.04	+	None	Promoter
ATP13A4 (5)	rs62287089	3.90E-07	0.001	0.04	-	None	None
TAPT1 (16)	rs33980476	4.70E-07	0.0011	0.043	-	None	None
RANBP17 (9)	rs77420825	7.60E-07	0.0011	0.043	+	None	None
RP11-108M9.4 (1)	rs2761505	1.30E-06	0.0011	0.043	-	None	None
AKAP2 (0)	rs79916100	2.90E-07	0.0011	0.043	-	None	None
TDRKH (1)	rs1623480	7.10E-07	0.0011	0.043	-	Enhancer	Enhancer
RP11-439E19.7 (0)	rs3007408	7.60E-07	0.0011	0.043	+	None	None
DNM1 (27)	rs11549266	2.10E-06	0.0011	0.043	+	Transcription	Transcription
CCT8 (9)	rs9976799	9.70E-07	0.0011	0.043	+	None	None
RASAL1 (2)	rs16942628	6.40E-07	0.0011	0.043	+	Enhancer	Enhancer
ALG8 (1)	rs616848	8.00E-07	0.0011	0.043	+	None	None
H1FX-AS1 (0)	rs2811429	9.00E-07	0.0011	0.043	+	Enhancer	Enhancer
SBF2 (20)	rs113481154	1.60E-07	0.0011	0.043	-	None	None
ERCC6L2 (20)	rs10512242	8.80E-07	0.0011	0.043	+	None	None
RP11-102F4.2 (1)	rs146850204	1.90E-07	0.0011	0.043	+	None	None
ARNTL2 (3)	rs10842953	9.30E-07	0.0011	0.043	+	Enhancer	Enhancer
RP11-280H21.1 (2)	rs79198321	4.50E-08	0.0011	0.043	+	None	None
IMPDH1 (0)	rs13230155	2.60E-06	0.0011	0.043	-	Enhancer	None
AP000688.29 (1)	rs112953000	7.40E-07	0.0011	0.043	-	None	Repressed
STXBP2 (7)	rs2303115	4.40E-07	0.0011	0.043	-	None	None
SYK (7)	rs4744514	1.30E-06	0.0011	0.043	+	Transcription	None
PRSS3 (4)	rs760098	3.70E-07	0.0011	0.043	+	None	None
TCEA3 (4)	rs2902874	1.60E-06	0.0011	0.043	+	None	None
VDR (9)	rs3890734	5.80E-07	0.0011	0.043	-	None	None
IL32 (0)	rs78997174	8.50E-07	0.0011	0.043	-	None	None
UPB1 (6)	rs5751984	6.00E-07	0.0011	0.043	+	None	None
CALM2 (10)	rs34990437	4.10E-07	0.0011	0.043	+	Enhancer	Enhancer
ITSN2 (41)	rs13401241	1.10E-06	0.0011	0.043	+	Enhancer	Enhancer
RGL3 (4)	rs317915	1.90E-06	0.0011	0.043	+	Transcription	None
IL17B (2)	rs1009424	1.30E-06	0.0011	0.043	+	None	Enhancer
ATP6V0A1 (11)	rs56317044	1.80E-06	0.0011	0.043	+	None	None
RP11-384K6.6 (3)	rs6833597	1.20E-06	0.0011	0.043	-	None	None
SPTLC1 (10)	rs12336689	9.10E-07	0.0011	0.043	-	None	None
KRR1 (6)	rs201057510	1.60E-06	0.0011	0.043	+	None	Enhancer
COG5 (6)	rs7786186	2.50E-07	0.0011	0.043	+	Enhancer	None
LINC00035 (1)	rs7806956	1.70E-06	0.0011	0.043	-	Enhancer	Enhancer
RHOH (6)	rs58826568	3.40E-08	0.0011	0.043	+	None	None
RBM27 (19)	rs959237	2.10E-06	0.0011	0.043	-	None	None
MAN2C1 (7)	rs4886683	3.30E-06	0.0011	0.043	+	Repressed	None
MUC4 (0)	rs56022806	4.00E-07	0.0011	0.043	+	None	None
AL589743.1 (5)	rs7401094	2.30E-06	0.0011	0.043	-	None	None
KIDINS220 (5)	rs13412882	6.90E-07	0.0011	0.043	-	None	Repressed
SLC25A46 (6)	rs2416238	1.10E-06	0.0011	0.043	+	None	None
RP1-149A16.17 (0)	rs5994542	6.30E-07	0.0011	0.043	-	Repressed	Repressed
RP11-401O9.3 (0)	rs11651428	9.20E-07	0.0011	0.043	+	Repressed	None
FBXO7 (8)	rs2285089	6.00E-07	0.0011	0.043	-	None	None
ZFYVE26 (28)	rs28610956	3.10E-07	0.0011	0.043	-	None	None
PTPRF (18)	rs841565	1.80E-06	0.0011	0.043	-	None	None
FRRS1 (1)	rs2392047	2.30E-06	0.0011	0.043	-	None	None
INSC (2)	rs10832529	1.60E-06	0.0011	0.043	-	None	None
RP11-680G24.5 (0)	rs62036867	3.70E-07	0.0011	0.043	-	Enhancer	Enhancer
EYA4 (8)	rs7747507	5.70E-09	0.0011	0.043	-	Repressed	None

TFRC (16)	rs2344654	7.70E-07	0.0011	0.043	+	Enhancer	Enhancer
SOAT1 (3)	rs11126	1.60E-06	0.0011	0.043	+	None	None
SEMA6A (20)	rs7712671	1.10E-06	0.0011	0.043	-	None	None
ABCF1 (11)	rs114398276	2.60E-07	0.0011	0.043	+	None	None
LINC00174 (5)	rs147104756	1.70E-06	0.0011	0.043	+	None	None
NCEH1 (1)	rs1799598	4.70E-07	0.0011	0.043	-	Enhancer	Enhancer
RFX6 (16)	rs2229079	6.40E-07	0.0011	0.043	-	None	None
MRPL34 (4)	rs12976380	7.90E-07	0.0011	0.043	+	Enhancer	None
ALKBH2 (2)	rs4766490	4.90E-07	0.0011	0.043	+	Enhancer	Enhancer
RP11-403P17.5 (1)	rs72790421	2.50E-06	0.0011	0.043	+	None	None
LY6H (0)	rs7835985	7.00E-07	0.0011	0.043	-	Repressed	Promoter
RP13-516M14.2 (0)	rs113441289	8.10E-07	0.0012	0.047	-	None	None
AP000688.14 (4)	rs192397973	1.50E-06	0.0012	0.047	-	None	None
PLA2G4C (23)	rs434371	1.20E-06	0.0012	0.047	-	Enhancer	Enhancer
ART5 (3)	rs61878490	3.50E-07	0.0012	0.047	+	None	None
TMEM232 (28)	rs6896041	2.10E-08	0.0012	0.047	+	None	None
STK39 (3)	rs200297735	1.50E-06	0.0012	0.047	-	None	None
GTPBP4 (4)	rs816603	8.60E-07	0.0012	0.047	+	Enhancer	Enhancer
C19orf44 (2)	rs7251415	1.70E-06	0.0012	0.047	+	Enhancer	Enhancer
FNIP2 (3)	rs35631953	1.50E-06	0.0012	0.047	-	None	None
ZNF732 (1)	rs111321884	7.10E-07	0.0012	0.047	+	None	None
SMCO4 (0)	rs7118644	7.20E-07	0.0012	0.047	-	Enhancer	None
FNDC3A (26)	rs7322908	2.00E-06	0.0012	0.047	-	Transcription	None
C3orf55 (3)	rs6768906	1.10E-06	0.0012	0.047	-	None	None
GCGR (4)	rs79700919	5.70E-07	0.0012	0.047	+	None	None
DAD1 (0)	rs5742830	4.70E-07	0.0012	0.047	-	Insulator	Enhancer
KDM3A (18)	rs7559420	1.60E-06	0.0012	0.047	-	Transcription	Transcription
BTBD11 (7)	rs7979127	1.90E-06	0.0012	0.047	+	Transcription	Transcription
MARCH1 (0)	rs1992441	1.00E-06	0.0012	0.047	-	Transcription	Transcription
PGS1 (2)	rs11656568	8.80E-07	0.0012	0.047	+	None	Enhancer
ERLIN1 (7)	rs792697	1.30E-06	0.0012	0.047	+	None	Repressed
MYO3A (11)	rs11598844	6.40E-07	0.0012	0.047	-	Transcription	None
RP11-517C16.2 (0)	rs41367549	1.10E-07	0.0012	0.047	+	None	None
RARS (15)	rs199533891	9.90E-07	0.0012	0.047	+	None	None
RP11-44N21.4 (4)	rs1003635	5.80E-07	0.0012	0.047	+	None	None
CENPP (0)	rs10123080	3.70E-07	0.0012	0.047	+	None	None
CROCC (10)	rs2610598	9.40E-07	0.0012	0.047	-	None	None
RETNLB (3)	rs192918145	5.40E-07	0.0012	0.047	+	None	None
HHLA3 (1)	rs58696538	2.20E-06	0.0012	0.047	-	Transcription	None
RP11-410N8.3 (0)	rs117104588	1.30E-06	0.0012	0.047	+	Enhancer	Enhancer
ASB5 (10)	rs35821163	1.00E-06	0.0012	0.047	+	None	None
CLDN7 (1)	rs2654188	7.20E-07	0.0012	0.047	+	None	None
DRD4 (1)	rs72844728	3.90E-07	0.0012	0.047	+	Enhancer	Enhancer
PWRN1 (14)	rs11161066	6.00E-07	0.0012	0.047	-	None	None
PIP4K2B (0)	rs1043515	1.10E-06	0.0012	0.047	-	Transcription	Transcription
ZCCHC24 (4)	rs2002688	2.80E-07	0.0012	0.047	+	Enhancer	Enhancer
IL1RL2 (7)	rs2241132	6.60E-07	0.0012	0.047	-	Enhancer	Promoter
FGD5-AS1 (5)	rs2733585	1.10E-06	0.0013	0.05	+	Transcription	Transcription
PTPN21 (16)	rs61980197	4.90E-07	0.0013	0.05	-	Enhancer	Enhancer
ATF1 (9)	rs1106725	1.30E-06	0.0013	0.05	-	None	None
AUH (11)	rs2281921	1.20E-06	0.0013	0.05	+	None	Enhancer
VTCN1 (3)	rs73006003	1.40E-06	0.0013	0.05	-	Enhancer	Enhancer
PRMT1 (4)	rs838136	7.70E-07	0.0013	0.05	-	Promoter	Promoter
LIPA (17)	rs304502	1.60E-06	0.0013	0.05	+	None	Enhancer
AK5 (15)	rs58073982	2.20E-06	0.0013	0.05	-	Transcription	Transcription
TLR4 (0)	rs35946190	1.10E-06	0.0013	0.05	-	None	None
LPCAT2 (0)	rs6499763	9.40E-07	0.0013	0.05	-	Repressed	Repressed
ZNF720 (6)	rs55794034	3.50E-06	0.0013	0.05	+	None	None
APMAP (1)	rs6050929	1.40E-06	0.0013	0.05	+	Enhancer	None
ISPD (10)	rs12670755	1.20E-06	0.0013	0.05	-	None	None
REP15 (0)	rs144049521	7.10E-07	0.0013	0.05	+	None	None
SUDS3 (12)	rs2036314	1.40E-06	0.0013	0.05	+	None	None
LRRC55 (1)	rs1941035	2.00E-06	0.0013	0.05	+	None	None
RP11-809O17.1 (0)	rs11774410	6.70E-07	0.0013	0.05	-	None	None
FAM159B (0)	rs4527552	2.00E-06	0.0013	0.05	+	Enhancer	Enhancer
LRP4 (11)	rs12284298	2.10E-06	0.0013	0.05	-	Enhancer	Enhancer
ACBD4 (8)	rs16939879	8.60E-07	0.0013	0.05	+	None	None
ADCK1 (14)	rs8021165	1.10E-06	0.0013	0.05	-	None	None
FGFR2 (3)	rs2935717	8.40E-07	0.0013	0.05	+	None	Repressed
RNF6 (1)	rs7338440	1.10E-06	0.0013	0.05	+	Enhancer	None
RP11-137L10.6 (10)	rs10824192	4.30E-06	0.0013	0.05	-	None	None
ZNRD1 (1)	rs116028867	1.60E-06	0.0013	0.05	+	None	None
PLEKHG5 (13)	rs11806429	1.30E-06	0.0013	0.05	-	Promoter	Promoter
DAB2 (18)	rs10512696	9.40E-07	0.0013	0.05	+	Promoter	Promoter
RP11-27N21.3 (5)	rs2440660	1.60E-06	0.0013	0.05	-	None	None
TSTD2 (0)	rs2805775	1.20E-06	0.0013	0.05	+	Transcription	None
EIF2AK1 (8)	rs35962539	4.30E-07	0.0013	0.05	+	None	None
CAPZB (11)	rs1546263	4.20E-07	0.0013	0.05	+	Enhancer	Enhancer
USP45 (4)	rs4504482	1.50E-06	0.0013	0.05	-	None	None
RP11-88H9.2 (3)	rs1545300	5.90E-07	0.0013	0.05	+	Enhancer	Enhancer
MRO (7)	rs8090501	2.30E-06	0.0013	0.05	-	None	None
EFTUD2 (29)	rs3826425	1.70E-06	0.0013	0.05	-	None	None
MDH1 (10)	rs6754730	1.80E-06	0.0013	0.05	-	None	None
TMEM179B (1)	rs117926699	1.50E-06	0.0013	0.05	-	Enhancer	None
VAC14 (14)	rs8053749	1.50E-06	0.0013	0.05	+	None	Enhancer
RP13-735L24.1 (3)	rs74062850	5.40E-07	0.0013	0.05	-	Promoter	Promoter
AC003986.5 (1)	rs17435661	5.30E-07	0.0013	0.05	+	Enhancer	Enhancer
PI4K2B (1)	rs62411562	3.60E-07	0.0013	0.05	-	Transcription	Enhancer
