# Supplementary material for: Transcript Expression Data from Human Islets Links Regulatory Signals from Genome-Wide Association Studies for Type 2 Diabetes and Glycemic Traits to Their Downstream Effectors
Source: PLoS Genet. 2015 Dec 1;11(12):e1005694. doi: 10.1371/journal.pgen.1005694 (PMC4666611; doi:10.1371/journal.pgen.1005694)
Supplement: S2 Table — (PDF) [file pgen.1005694.s002.pdf]

Supplementary Table 2. Detailed information on the 21 reported index variants for T2D and glycemic traits co-inciding with islet eQTLs

	Locus	Trait	Reported associated variant	Alleles (risk/non-risk)	eQTL Gene	eQTL Exon	Best eQTL in recombination interval	*r2* between best exon-eQTL and GWAS variants	Exon-eQTL variant *p*-value	Exon-eQTL variant permuted *p*-value	Exon-eQTL variant *q*-value (derived from permuted *p*-values)	Exon-eQTL variant *p*-value conditional on GWAS variant	Exon-eQTL variant *ß* for risk allele	Islet active chromatin annotation
**Study-wide signficant findings**	*ABO**	Disposition index	rs505922	C/T	*ABO*	3	rs676996	0.96	1.3E-14	1.0E-04	5.7E-03	0.77	0.86	-
	ANK1	Corrected insulin response	rs12549902	A/G	*NKX6-3*	0	rs12549902	1.00	6.1E-07	1.0E-03	4.0E-02	1.00	-0.36	Enhancer; Promoter
	AP3S2	T2D	rs2028299	A/C	*AP3S2*	6	rs4932261	0.97	5.1E-09	1.0E-04	5.7E-03	0.30	-0.55	Enhancer
	ARAP1	T2D	rs1552224	A/C	*STARD10*	7	rs7109575	0.97	8.5E-08	4.0E-04	1.9E-02	0.97	-0.39	Enhancer; Promoter
	ARAP1	Fasting glucose; Fasting proinsulin	rs11603334	G/A	*STARD10*	7	rs7109575	0.97	8.5E-08	4.0E-04	1.9E-02	0.97	-0.39	Enhancer; Promoter
	CDC123/CAMK1D	T2D	rs11257655	T/C	*CAMK1D*	10	rs7100710	0.82	1.7E-07	2.0E-04	1.1E-02	0.21	0.61	Enhancer
	DGKB/TMEM195	T2D; Fasting glucose; HOMA-B	rs2191349	T/G	*DGKB*	17	rs10231021	0.90	5.3E-07	1.0E-03	4.0E-02	0.41	0.44	Enhancer; Promoter
	MADD	Fasting proinsulin	rs10838687	T/G	*ACP2*	0	rs12222581	0.90	4.6E-08	1.0E-04	5.7E-03	0.92	0.31	Enhancer
	MADD	Fasting proinsulin	rs10501320	G/C	*MADD*	7	rs10501320	1.00	4.7E-09	1.0E-04	5.7E-03	1.00	0.25	Enhancer
	MADD	Fasting glucose	rs7944584	A/T	*MADD*	7	rs10501320	0.95	4.7E-09	1.0E-04	5.7E-03	0.53	0.25	Enhancer
	WARS	Fasting glucose	rs3783347	G/T	*WARS*	16	rs1998902	0.99	3.2E-34	1.0E-04	5.7E-03	1.00	-1.58	Enhancer; Promoter
**Additional findings with permuted *p* <0.05**	*ADCY5*	T2D; Fasting glucose; HOMA-B	rs11708067	A/G	*ADCY5*	13	rs11708067	1.00	1.1E-05	8.4E-03	1.8E-01	1.00	-0.44	Enhancer
	ADCY5	2-hour glucose	rs2877716	C/T	*ADCY5*	13	rs11708067	0.85	1.1E-05	8.4E-03	1.8E-01	0.45	-0.44	Enhancer
	AMT	Fasting glucose	rs11715915	C/T	*RBM6*	9	rs148734725	0.87	2.0E-05	5.9E-03	1.5E-01	0.39	-0.23	Enhancer; Promoter
	DGKB/TMEM195	T2D; Fasting glucose	rs17168486	T/C	*DGKB*	19	rs10281892	0.90	8.4E-06	9.3E-03	1.9E-01	0.96	0.52	Enhancer; Promoter
	FADS1	Fasting glucose; HOMA-B	rs174550	T/C	*FADS1*	12	rs28456	0.82	5.3E-06	1.6E-02	2.6E-01	0.22	0.31	Enhancer; Promoter
	MTNR1B	T2D; Fasting glucose; HOMA-B; Corrected insulin response	rs10830963	G/C	*MTNR1B*	0	rs10830963	1.00	1.1E-05	1.5E-02	2.5E-01	1.00	0.40	Enhancer
	PCSK1	Fasting glucose	rs4869272	T/C	*CTD-2260A17.2*	1	rs4869273	0.98	4.4E-05	2.6E-02	3.3E-01	0.41	0.58	Enhancer
	TMEM163	T2D	rs6723108	T/G	*MGAT5*	15	rs1942055	0.94	1.8E-05	2.4E-02	3.2E-01	1.00	0.26	Enhancer
	*ZFAND6**	T2D	rs11634397	G/A	*LINC00927*	0	rs34140486	0.82	5.5E-06	1.6E-02	2.6E-01	0.21	-0.54	-
	ZMIZ1	T2D	rs12571751	A/G	*ZMIZ1*	10	rs703967	0.98	2.7E-05	3.8E-02	3.9E-01	0.76	0.13	Enhancer

*These loci are excluded from further analysis since there was no overlap with active islet chromatin annotations
