# Supplementary material for: Transcript Expression Data from Human Islets Links Regulatory Signals from Genome-Wide Association Studies for Type 2 Diabetes and Glycemic Traits to Their Downstream Effectors
Source: PLoS Genet. 2015 Dec 1;11(12):e1005694. doi: 10.1371/journal.pgen.1005694 (PMC4666611; doi:10.1371/journal.pgen.1005694)
Supplement: S3 Table — (PDF) [file pgen.1005694.s003.pdf]

Supplementary Table 3. Overlap between islet exon-eQTLs and the gene-level eQTLs from Fadista *et al.*

Gene (exon)	Fadista gene	Fadista SNP	Direction of effect	Fadista direction of effect	Sharing
CYP3A5 (10)	CYP3A5	rs10238028	+	+	shared_variant
EPDR1 (3)	EPDR1	rs10260487	+	+	shared_variant
MAG (12)	MAG	rs10411704	-	-	shared_variant
ZNF880 (4)	ZNF880	rs10445586	-	+	shared_variant
SNHG5 (3)	SNHG5	rs1059307	+	+	shared_variant
RPS26 (0)	RPS26	rs10876864	-	-	shared_variant
HLA-DRA (4)	HLA-DRA	rs111232699	-	-	shared_variant
AGA (0)	AGA	rs11131799	+	+	shared_variant
LYRM4 (0)	LYRM4	rs111557465	+	+	shared_variant
UBE2U (2)	UBE2U	rs11208378	+	+	shared_variant
LRRIQ3 (10)	LRRIQ3	rs11210442	-	-	shared_variant
SLC1A6 (0)	SLC1A6	rs112816712	+	+	shared_variant
CRIPAK (0)	CRIPAK	rs113727613	+	+	shared_variant
HEPHL1 (4)	HEPHL1	rs1138800	+	+	shared_variant
PAX8 (0)	PAX8	rs1139015	-	-	shared_variant
HLA-DPB1 (2)	HLA-DPB1	rs114988017	-	-	shared_variant
HLA-DMA (5)	HLA-DMA	rs115334078	+	+	shared_variant
CIB2 (0)	CIB2	rs11547207	+	+	shared_variant
HLA-C (0)	HLA-C	rs116108880	-	-	shared_variant
NDUFA10 (12)	NDUFA10	rs11684044	-	-	shared_variant
PCDHB8 (0)	PCDHB8	rs11741863	+	+	shared_variant
LRRC37A2 (1)	LRRC37A2	rs117692492	-	-	shared_variant
ITGB3BP (0)	ITGB3BP	rs11804843	+	+	shared_variant
SERPINC1 (4)	SERPINC1	rs12077782	+	+	shared_variant
EXOSC6 (0)	EXOSC6	rs12102803	-	-	shared_variant
PAQR6 (0)	PAQR6	rs12128955	-	-	shared_variant
KHDC1 (3)	KHDC1	rs12154185	-	+	shared_variant
CCDC163P (0)	CCDC163P	rs12729974	-	-	shared_variant
NTPCR (2)	NTPCR	rs12743233	-	-	shared_variant
C11orf54 (2)	C11orf54	rs1284141	-	-	shared_variant
ZNF100 (0)	ZNF100	rs12972593	-	-	shared_variant
TMEM18 (4)	TMEM18	rs12990777	+	-	shared_variant
CDC25A (2)	CDC25A	rs13068288	+	+	shared_variant
CCDC85A (1)	CCDC85A	rs1319240	-	-	shared_variant
PTPLAD2 (6)	PTPLAD2	rs13284328	+	+	shared_variant
ESPNL (9)	ESPNL	rs13382446	+	+	shared_variant
NQO2 (1)	NQO2	rs138616686	-	-	shared_variant
REP15 (0)	REP15	rs144049521	+	+	shared_variant
MICB (0)	MICB	rs147232276	-	-	shared_variant
HSD17B12 (14)	HSD17B12	rs1518822	-	+	shared_variant
TMED6 (0)	TMED6	rs153060	-	-	shared_variant
ZNF107 (6)	ZNF107	rs1596929	-	-	shared_variant
ARHGEF35 (0)	ARHGEF35	rs1731353	-	-	shared_variant
C9orf135 (0)	C9orf135	rs17518268	+	+	shared_variant
HLA-DQA1 (3)	HLA-DQA1	rs17612669	-	-	shared_variant
CECR1 (0)	CECR1	rs17807317	+	+	shared_variant
NOM1 (3)	NOM1	rs1833140	+	-	shared_variant
ARL17A (1)	ARL17B+ARL17A	rs190544400	+	+	shared_variant
XKR9 (0)	XKR9	rs1993862	+	+	shared_variant
TAPBPL (3)	TAPBPL	rs2041387	+	+	shared_variant
VWDE (21)	VWDE	rs2119141	+	+	shared_variant
SRR (3)	SRR	rs2131700	-	-	shared_variant
PPIL3 (0)	PPIL3	rs2136600	-	-	shared_variant
FN3KRP (4)	FN3KRP	rs2246577	-	+	shared_variant
MAP6D1 (0)	MAP6D1	rs2255015	-	-	shared_variant
ACP6 (3)	ACP6	rs2275552	+	+	shared_variant
MYOM2 (38)	MYOM2	rs2280902	-	-	shared_variant
DNAJC15 (0)	DNAJC15	rs2281778	-	-	shared_variant

SLC5A11 (11)	SLC5A11	rs2303083	+	+	shared_variant
PSG4 (10)	PSG4	rs2354278	-	-	shared_variant
GBP3 (0)	GBP3	rs2390677	-	-	shared_variant
L1TD1 (2)	L1TD1	rs2457823	+	+	shared_variant
DDX11 (0)	DDX11	rs2543246	-	-	shared_variant
SGCA (1)	SGCA	rs2586478	-	-	shared_variant
ENOSF1 (11)	ENOSF1	rs2741168	+	+	shared_variant
ERAP2 (1)	ERAP2	rs2927608	+	+	shared_variant
KLHDC7A (0)	KLHDC7A	rs2992735	+	+	shared_variant
CDSN (0)	CDSN	rs3094199	-	-	shared_variant
PSORS1C1 (0)	PSORS1C1	rs3094221	+	+	shared_variant
ST7L (0)	ST7L	rs351372	-	-	shared_variant
CCBL2 (6)	RBMXL1+CCBL2	rs35594043	-	-	shared_variant
ACOT4 (1)	ACOT4	rs3742819	+	+	shared_variant
ZFP57 (0)	ZFP57	rs374317	+	+	shared_variant
GPX7 (1)	GPX7	rs3753754	-	-	shared_variant
LY75 (0)	LY75+CD302+LY75-CD302	rs3792198	+	+	shared_variant
PRPH2 (0)	PRPH2	rs3818087	+	+	shared_variant
ATP2C2 (0)	ATP2C2	rs41367549	+	+	shared_variant
NDUFV3 (3)	NDUFV3	rs4148974	-	-	shared_variant
LRRC69 (11)	LRRC69	rs4551303	-	-	shared_variant
GLIPR1L2 (0)	GLIPR1L2	rs4565940	-	-	shared_variant
XRCC6BP1 (0)	XRCC6BP1	rs4590915	-	-	shared_variant
APBB1IP (1)	APBB1IP	rs4749125	-	-	shared_variant
PYROXD1 (10)	PYROXD1	rs4762826	-	-	shared_variant
GRHPR (2)	GRHPR	rs4878690	+	+	shared_variant
AP3S2 (6)	AP3S2+C15orf38+C15orf38-AP3S2	rs4932261	-	-	shared_variant
ABO (0)	ABO	rs494242	+	+	shared_variant
GSTM3 (0)	GSTM3	rs4970777	+	+	shared_variant
SH3YL1 (1)	SH3YL1	rs59937473	-	-	shared_variant
FAM182B (0)	FAM182B	rs6050988	+	+	shared_variant
WARS2 (0)	WARS2	rs61808892	-	-	shared_variant
ATP12A (1)	ATP12A	rs61948108	+	+	shared_variant
C15orf57 (1)	C15orf57	rs62019879	-	-	shared_variant
LDHC (0)	LDHC	rs6486421	-	-	shared_variant
APOBEC3G (8)	APOBEC3G	rs6519166	-	-	shared_variant
THNSL2 (10)	THNSL2	rs6547757	+	+	shared_variant
TCL6 (7)	TCL6	rs6575528	-	-	shared_variant
FOXRED1 (0)	FOXRED1	rs659551	+	+	shared_variant
TRMT61B (4)	TRMT61B	rs6718662	-	-	shared_variant
MRPL21 (0)	MRPL21	rs678904	-	-	shared_variant
PON1 (1)	PON1	rs705381	-	-	shared_variant
XRRA1 (1)	XRRA1	rs7105085	+	+	shared_variant
CAT (1)	CAT	rs7120960	-	-	shared_variant
NDUFA3 (3)	NDUFA3	rs7253859	+	+	shared_variant
RHD (10)	RHD	rs72660908	-	-	shared_variant
KCND3 (0)	KCND3	rs72694622	+	+	shared_variant
ELFN2 (0)	ELFN2	rs7286065	-	-	shared_variant
ATP6AP1L (9)	ATP6AP1L	rs73138787	+	+	shared_variant
FAM118A (10)	FAM118A	rs738177	-	-	shared_variant
GPR149 (0)	GPR149	rs74761378	+	+	shared_variant
C17orf97 (1)	C17orf97	rs7502594	+	+	shared_variant
NUDT2 (1)	NUDT2	rs77025004	-	+	shared_variant
THSD7B (9)	THSD7B	rs77055332	+	+	shared_variant
FRA10AC1 (0)	FRA10AC1	rs7909452	-	-	shared_variant
C1QL3 (0)	C1QL3	rs7909832	+	+	shared_variant
SNX19 (11)	SNX19	rs7949722	-	-	shared_variant
IQCB1 (0)	IQCB1	rs80047370	+	+	shared_variant
TRAPPC4 (1)	MIR3656+TRAPPC4	rs8192696	+	+	shared_variant
HLA-DRB1 (0)	HLA-DRB1	rs9270838	-	-	shared_variant
HLA-DRB5 (0)	HLA-DRB5	rs9271147	-	-	shared_variant
HLA-DQB1 (0)	HLA-DQB1	rs9274284	-	-	shared_variant

TPMT (4)	TPMT	rs9465105	-	-	shared_variant
PEX5L (0)	PEX5L	rs9825224	+	+	shared_variant
NOMO3 (13)	NOMO3	.	.	.	shared_gene
ELP3 (21)	ELP3	.	.	.	shared_gene
EPHX2 (6)	EPHX2	.	.	.	shared_gene
AKAP2 (0)	AKAP2+PALM2+PALM2-AKAP2	.	.	.	shared_gene
ATG10 (0)	ATG10	.	.	.	shared_gene
HLA-B (0)	HLA-B	.	.	.	shared_gene
TSPAN12 (0)	TSPAN12	.	.	.	shared_gene
PM20D1 (0)	PM20D1	.	.	.	shared_gene
ZNF83 (0)	ZNF83	.	.	.	shared_gene
CHURC1 (1)	CHURC1	.	.	.	shared_gene
CPNE1 (1)	RBM12+CPNE1	.	.	.	shared_gene
EFHB (15)	EFHB	.	.	.	shared_gene
EFCAB2 (0)	EFCAB2	.	.	.	shared_gene
NARS2 (0)	NARS2	.	.	.	shared_gene
HLA-A (2)	HLA-A	.	.	.	shared_gene
CHPT1 (9)	CHPT1	.	.	.	shared_gene
FMN1 (17)	FMN1	.	.	.	shared_gene
AGAP7 (0)	AGAP7	.	.	.	shared_gene
TDRD5 (15)	TDRD5	.	.	.	shared_gene
ZNF208 (3)	ZNF208	.	.	.	shared_gene
C15orf40 (1)	C15orf40	.	.	.	shared_gene
EFCAB1 (4)	EFCAB1	.	.	.	shared_gene
C8orf59 (1)	C8orf59	.	.	.	shared_gene
HCG9 (0)	HCG9	.	.	.	shared_gene
ARAP1 (37)	ARAP1	.	.	.	shared_gene
SURF1 (5)	SURF1	.	.	.	shared_gene
NAAA (0)	NAAA	.	.	.	shared_gene
ACSM1 (3)	ACSM1	.	.	.	shared_gene
ZNF732 (1)	ZNF732	.	.	.	shared_gene
MLC1 (5)	MLC1	.	.	.	shared_gene
C4orf33 (7)	C4orf33	.	.	.	shared_gene
ZNF584 (1)	ZNF584	.	.	.	shared_gene
AGXT (2)	AGXT	.	.	.	shared_gene
TMEM45A (2)	TMEM45A	.	.	.	shared_gene
POMZP3 (0)	POMZP3	.	.	.	shared_gene
DCTN5 (6)	DCTN5	.	.	.	shared_gene
PEX6 (0)	PEX6	.	.	.	shared_gene
OR7D2 (0)	OR7D2	.	.	.	shared_gene
STYXL1 (2)	STYXL1	.	.	.	shared_gene
TLL2 (13)	TLL2	.	.	.	shared_gene
C2orf54 (0)	C2orf54	.	.	.	shared_gene
SIGLEC12 (0)	SIGLEC12	.	.	.	shared_gene
UROS (0)	UROS	.	.	.	shared_gene
ABCB9 (2)	ABCB9	.	.	.	shared_gene
CYP2C9 (6)	CYP2C9	.	.	.	shared_gene
ULK4 (0)	ULK4	.	.	.	shared_gene
PRUNE2 (15)	PRUNE2	.	.	.	shared_gene
FTSJ3 (0)	FTSJ3	.	.	.	shared_gene
NOTCH2NL (2)	NOTCH2NL	.	.	.	shared_gene
TSPAN33 (1)	TSPAN33	.	.	.	shared_gene
ZP3 (8)	ZP3	.	.	.	shared_gene
PSORS1C2 (0)	PSORS1C2	.	.	.	shared_gene
QSOX2 (1)	QSOX2	.	.	.	shared_gene
FAM106A (0)	FAM106A	.	.	.	shared_gene
ERAP1 (13)	ERAP1	.	.	.	shared_gene
SLC25A26 (2)	SLC25A26	.	.	.	shared_gene
IP6K3 (0)	IP6K3	.	.	.	shared_gene
DHRS4L2 (9)	DHRS4L2	.	.	.	shared_gene
HLA-DQA2 (2)	HLA-DQA2	.	.	.	shared_gene
CLIC6 (5)	CLIC6	.	.	.	shared_gene

CNGA1 (0)	CNGA1	.	.	.	shared_gene
GABRA5 (11)	GABRA5	.	.	.	shared_gene
SNX16 (0)	SNX16	.	.	.	shared_gene
DIP2B (27)	DIP2B	.	.	.	shared_gene
ZNF468 (4)	ZNF468	.	.	.	shared_gene
DDTL (0)	DDTL	.	.	.	shared_gene
OR3A2 (0)	OR3A2	.	.	.	shared_gene
CCDC67 (10)	CCDC67	.	.	.	shared_gene
UCA1 (1)	UCA1	.	.	.	shared_gene
USP40 (0)	USP40	.	.	.	shared_gene
PLEKHM1 (6)	MIR4315-2+PLEKHM1P+MIR4315-1+PLEKHM1	.	.	.	shared_gene
TTC12 (1)	TTC12	.	.	.	shared_gene
RNF212 (10)	RNF212	.	.	.	shared_gene
GSTM1 (0)	GSTM1	.	.	.	shared_gene
ST6GAL1 (22)	ST6GAL1	.	.	.	shared_gene
TIPIN (0)	TIPIN	.	.	.	shared_gene
SLC44A5 (2)	SLC44A5	.	.	.	shared_gene
NBPF3 (21)	NBPF3	.	.	.	shared_gene
KRT40 (1)	KRT40	.	.	.	shared_gene
PSORS1C3 (0)	PSORS1C3	.	.	.	shared_gene
PSMD13 (0)	PSMD13	.	.	.	shared_gene
ZNF98 (0)	ZNF98	.	.	.	shared_gene
MTNR1B (0)	MTNR1B	.	.	.	shared_gene
ARHGAP40 (8)	ARHGAP40	.	.	.	shared_gene
CCL4L2 (0)	CCL4L1+CCL4L2	.	.	.	shared_gene
MICA (0)	MICA	.	.	.	shared_gene
FUT9 (4)	FUT9	.	.	.	shared_gene
DMBT1 (11)	DMBT1	.	.	.	shared_gene
C10orf107 (0)	C10orf107	.	.	.	shared_gene
STOX1 (2)	STOX1	.	.	.	shared_gene
TSGA10 (2)	TSGA10	.	.	.	shared_gene
FUT10 (0)	FUT10	.	.	.	shared_gene
FLG (0)	FLG	.	.	.	shared_gene
ZNF786 (3)	ZNF786	.	.	.	shared_gene
