# Supplementary figures and images for: Transcript Expression Data from Human Islets Links Regulatory Signals from Genome-Wide Association Studies for Type 2 Diabetes and Glycemic Traits to Their Downstream Effectors
Source: PLoS Genet. 2015 Dec 1;11(12):e1005694. doi: 10.1371/journal.pgen.1005694 (PMC4666611; doi:10.1371/journal.pgen.1005694)

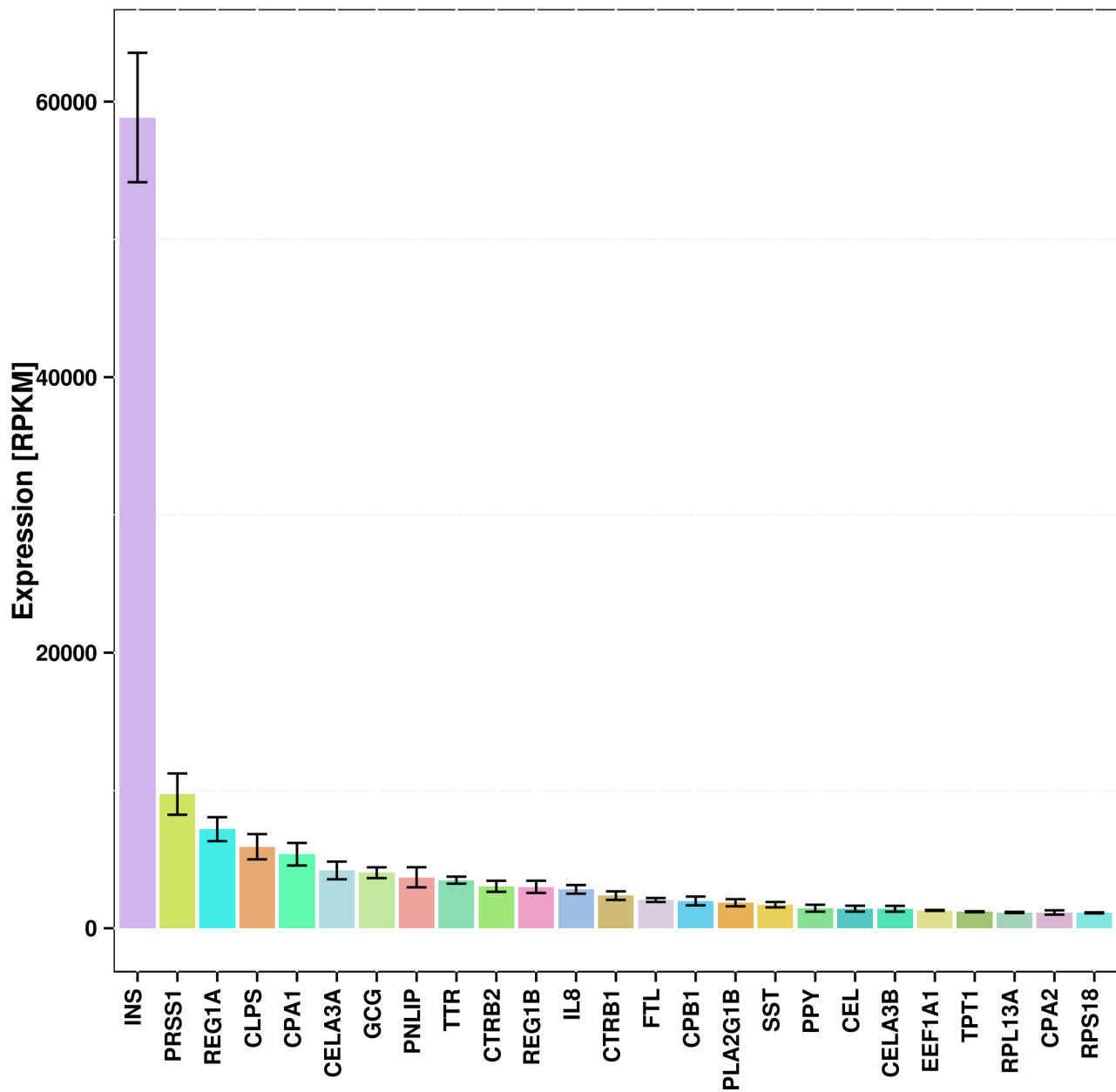

Supplement: S1 Fig — Expression was quantified as reads per million mapped reads per kilobase of transcript (RPKM). Error bars denote standard error of the mean. (PDF) [file pgen.1005694.s004.pdf]

**a**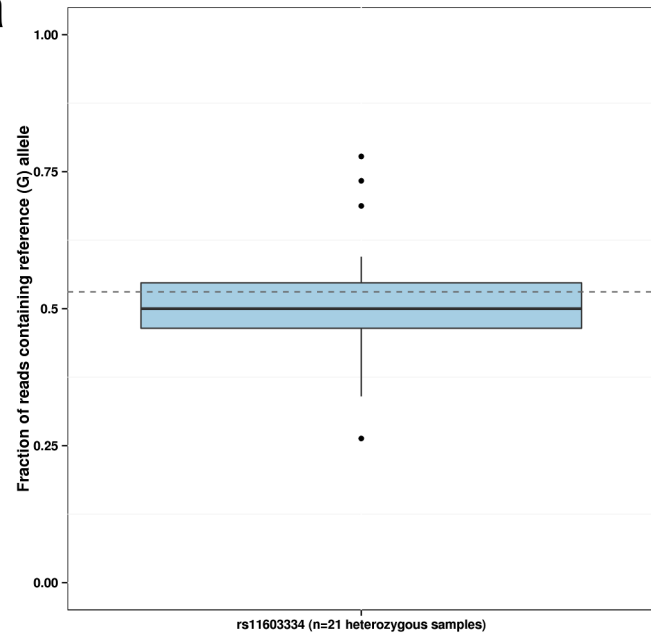**b**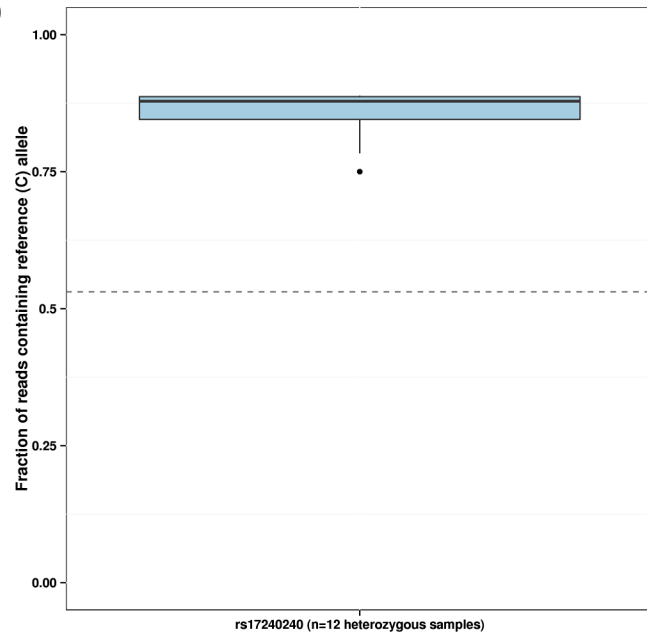**c**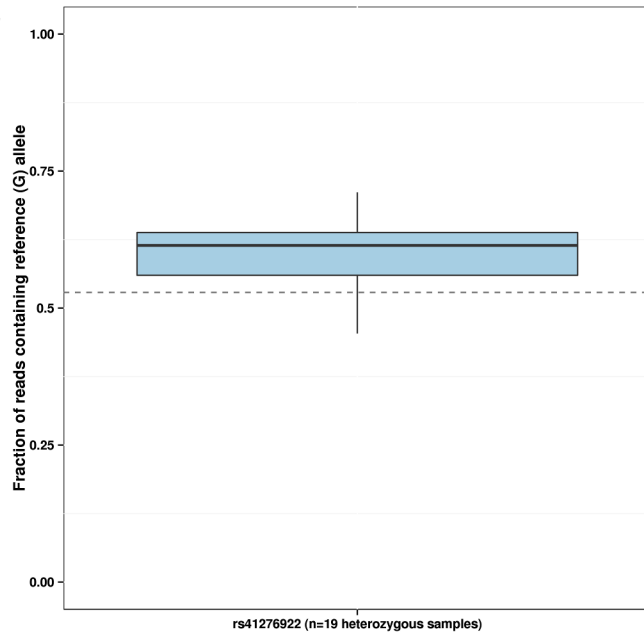

Supplement: S2 Fig — (a) Previously reported ASE variant in ARAP1 (rs11603334) associated with T2D and glycemic traits showed no significant (p>0.1) allelic imbalance in the human islet data. (b,c) Both previously reported ASE variants in ANPEP (rs17240240 and rs41276922), which are in very weak LD with the T2D signal at the AP3S2 locus, also show significant (p<0.01) ASE in this study. (PDF) [file pgen.1005694.s005.pdf]

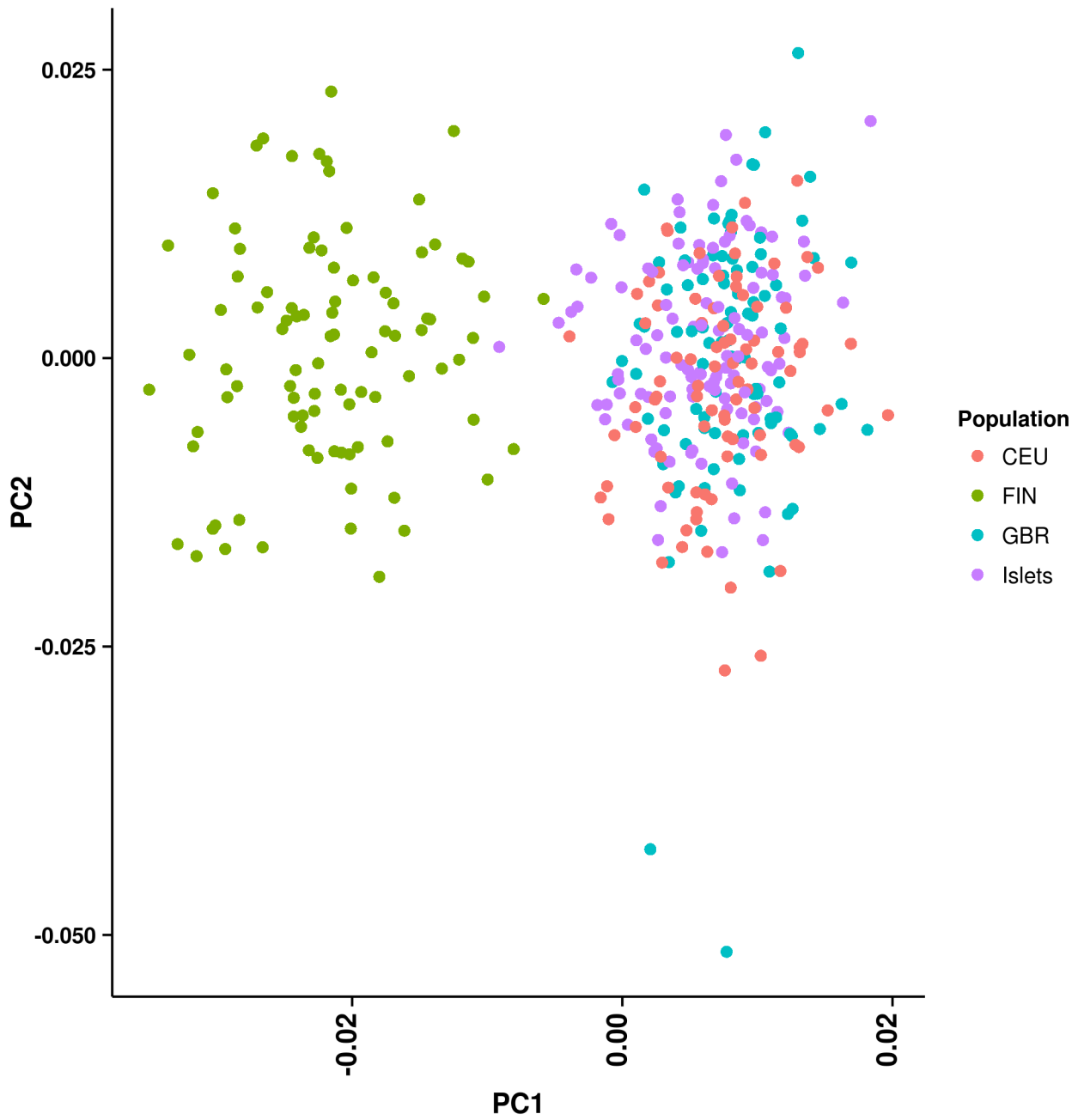

Supplement: S3 Fig — Principal component analysis of the 118 islet samples with the 1000 Genomes Northern European ancestry populations, computed using independent common (MAF > 1%) variants on chromosome 1. (PDF) [file pgen.1005694.s006.pdf]
